# Thermally programmed disassembly and reconstruction of 2D polymer platelets via melting-governed crystallization-driven self-assembly

**DOI:** 10.1038/s41467-026-77973-4

**Published:** 2026-10-03

**Authors:** Emre Turan, Maria C. Arno

**Affiliations:** 1 School of Chemistry, University of Birmingham, Edgbaston, Birmingham, UK; 2 Institute of Cancer and Genomic Sciences, University of Birmingham, Edgbaston, Birmingham, UK

**Keywords:** Supramolecular polymers, Nanoparticles, Self-assembly

## Abstract

Crystallization-driven self-assembly (CDSA) has emerged as a powerful methodology to precisely control the morphology and size of polymer nanostructures, enabling the formation of 1D and 2D nanomaterials. Yet, the melting behavior of polymer crystals grown in solution using this technique has remained largely unexplored. Here, we show that melting temperature is a structurally sensitive descriptor and an active design parameter for 2D poly(ε-caprolactone) platelets formed by seeded living CDSA. Using nano differential scanning calorimetry, we establish quantitative thickness-melting correlations consistent with Gibbs-Thomson theory for finite, solution-grown lamellae. Leveraging these insights, we engineer multilayer platelets with programmed thermal stability gradients that undergo rapid, selective disassembly under mild conditions (37 °C), yielding uniform hollow architectures within minutes. Upon cooling, released polymer chains undergo spatially confined recrystallization, allowing reversible reconstruction and self-healing of the platelet framework. This work establishes melting as a powerful thermodynamic handle for probing and reconfiguring CDSA-derived nanostructures in solution.

## Abstract (web summary)

‘Crystallization-driven self-assembly is a useful tool for the synthesis of polymer nanostructures with precise dimensions, but the melting behavior of polymer crystals grown in solution has remained largely unexplored. Here, the authors show that melting temperature is a structurally sensitive descriptor and an active design parameter for 2D platelets.’

## Introduction

Crystallization-driven self-assembly (CDSA) has been widely used to generate uniform, anisotropic nanostructures with precise control over size, shape, and dispersity^1–4^. Extensive studies have shown that 1D fibers and 2D platelets can be accessed in solution by tuning parameters such as polymer composition, block ratio, solvent selectivity, unimer-to-seed ratio, and growth temperature^5–9^. Moreover, the development of seeded ‘living’ CDSA methodologies has enabled predictable control over lateral dimensions, aspect ratio, and multilayer growth in 2D platelets^10–16^, leading to the formation of nanomaterials with applications in catalysis, energy, and healthcare among others^17–22^.

Despite considerable advances in the field, the thermal behavior of solution-grown polymer crystals remains surprisingly underexplored. In bulk semicrystalline polymers, melting temperature, lamellar thickness, and crystallinity are well-defined and routinely quantified^23,24^; by contrast, the melting behavior of nanoscale, solution-grown lamellae is often presumed to resemble bulk values or treated as a secondary characterization parameter^25^. This assumption overlooks a key distinction: CDSA-derived platelets are finite, defect-rich crystals formed under non-equilibrium conditions, where lamellar thickness, chain folding, and interfacial energetics differ fundamentally from bulk materials^26^. Hence, their melting behavior should not be expected to directly mirror that of bulk polymers.

Recent work has begun to highlight the importance of thermal history in CDSA systems, demonstrating that annealing protocols can improve size uniformity, suppress secondary nucleation, and enhance seed stability^27^. In particular, thermal annealing of 1D seeds under flow has been shown to increase crystallinity and improve subsequent ‘living’ CDSA efficiency^28^. Another report describes melting-induced morphological transitions, where heating above the melting temperature induced micellization of large crystalline aggregates^29^. Nevertheless, melting temperature has largely remained a passive descriptor in CDSA studies, and the extent to which it encodes structural information about solution-grown lamellae remains unclear. Moreover, thermal treatment has so far been used to refine existing structures^27,28^ rather than as an active handle to reconfigure two-dimensional crystalline architectures. This represents a notable knowledge gap, especially since traditional polymer crystallization theory establishes a strong relationship between lamellar thickness, crystallization temperature, and melting temperature through the Gibbs-Thomson relationship^30^ and Hoffman-Weeks method^31^. Extending these thermodynamic concepts from bulk polymers to finite, solution-grown 2D platelets offers a direct bridge between polymer crystallization physics and nanoscale self-assembly. This will enable predictive control over platelet stability, thickness, and hierarchical organization in solution, opening a framework in which melting can be deliberately harnessed to program structural evolution, selective disassembly, and reversible architectural transformation.

Herein, we investigate the melting behavior of 2D poly(ε-caprolactone) (PCL) platelets formed by seeded living CDSA and establish melting temperature as a quantitative structural probe. Using nano differential scanning calorimetry (nanoDSC), we decouple polymer chain length from lamellar thickness, revealing that platelet melting is governed jointly by molar mass, nanoscale dimensions, and crystallization conditions. Quantitative correlations between lamellar thickness and melting temperature are established, providing direct validation of Gibbs-Thomson behavior in finite, solution-grown 2D crystals.

Beyond serving as a structural descriptor, we show that melting can be harnessed as an active handle for morphological control. By quantitatively resolving the interplay between lamellar thickness and chain length, we define accessible thermal windows that allow stability to be programmed in advance and triggered on demand. By engineering bilayer platelets with deliberately mismatched thermal stabilities, we achieve selective thermal disassembly of targeted lamellae within minutes while preserving the surrounding crystalline framework, yielding well-defined hollow 2D platelets. Notably, this transformation proceeds within minutes under mild conditions (37 °C) and does not require chemical degradation^32^, extreme pH^33^, or subzero temperatures^34^, distinguishing it from previously reported routes to hollow platelet formation. Moreover, the released polymer chains remain intact and functional, undergoing spatially confined recrystallization upon cooling to enable temperature-directed reconstruction and self-healing, rather than irreversible fragmentation. This demonstrates that reversible structural reconfiguration of CDSA-derived 2D platelets in solution is possible without the need for chemical modification or external templating. Extending this concept, trilayer platelets with thermally labile intermediate layers were shown to undergo selective internal melting and spontaneous architectural decoupling into two discrete crystalline entities, demonstrating that programmed thermal stability can direct rapid, spatially resolved disassembly pathways beyond conventional CDSA strategies.

## Results

We employed seeded living CDSA of PCL homopolymer to generate uniform 2D platelets, with surface area controlled by the ratio of unimers added to a solution of 1D seeds. PCL served as the core-forming semicrystalline block, chosen for its biocompatibility, biodegradability, and broad potential in biological applications^35–37^. To isolate the intrinsic effects of crystallization parameters on the resulting melting temperatures, we examined different factors, including PCL chain length and lamellar thickness, using crystalline platelets prepared without a corona-forming block.

### Effect of PCL chain length on melting temperatures

**Fig. 1:**
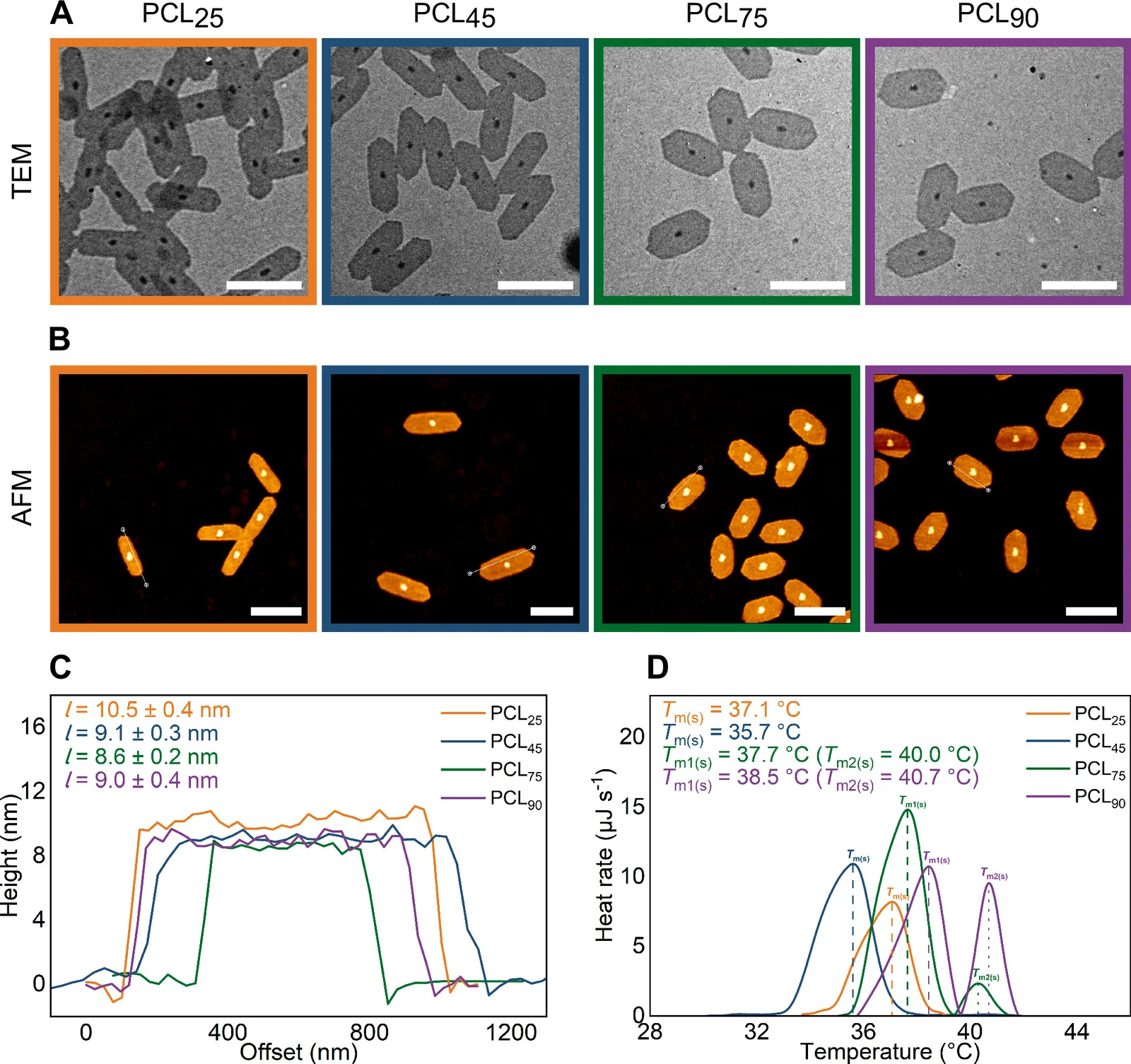
Formation, melting, and crystallinity of 2D PCL platelets with varying PCL lengths. **A** TEM micrographs, and **B** AFM images of 2D platelets formed from PCL with different degrees of polymerization: 25 (orange), 45 (blue), 75 (green), and 90 (purple) (left to right). All scale bars are equivalent to 1 µm. **C** Stacked AFM height profiles, and **D** plot of heat rate against temperature extracted from nanoDSC, depicting the melting temperatures of pure PCL platelets formed from varying degrees of polymerization; a linear baseline correction was applied prior to analysis (Supplementary Fig. 10).

Initially, we investigated the seeded growth of single-layer platelets from PCL chains of varying degrees of polymerization. To this end, differing lengths of PCL homopolymers (PCL_15_, PCL_25_, PCL_45_, PCL_75_, and PCL_90_) were synthesized via ring-opening polymerization of ε-caprolactone and fully characterized by ^1^H NMR spectroscopy and size exclusion chromatography (SEC) analysis (Supplementary Figs. 1–6). In all cases, the homopolymer, dissolved in chloroform, was added to a solution containing dispersed PCL_50_*-b-*poly(dimethylacrylamide)_200_ (PCL_50_*-b-*PDMA_200_) seeds in ethanol, which served as well-defined nucleation sites. A unimer-to-seed ratio of 10:1 was used to maintain consistent growth conditions across all experiments. All PCL lengths, except PCL_15_, were able to successfully grow epitaxially from PCL_50_*-b-*PDMA_200_ seeds, yielding uniform 2D platelets (Fig. 1A, B, corresponding frequency plots in Supplementary Fig. 7). In contrast, the self-assembly of PCL_15_ homopolymer yielded a mixture of high aspect ratio platelets and irregular platelets that seemed to crystallize around several slender platelets acting as seeds (Supplementary Fig. 8). We hypothesized that the short chain length of PCL_15_ prevented stable attachment and crystallization at the seed interface, resulting instead in spontaneous nucleation and uncontrolled growth in solution rather than epitaxial growth from 1D seeds. This limitation persisted even when crystallization was attempted at lower temperatures (4 °C and −20 °C, Supplementary Fig. 8), and therefore PCL_15_ was not selected for further studies.

Interestingly, platelets formed from various PCL homopolymers showed a systematic decrease in aspect ratios as chain length increased (ranging from 2.8 ± 0.4 for PCL_25_ to 1.8 ± 0.1 for PCL_90_) (Supplementary Fig. 9). This was ascribed to the different crystallization kinetics, with shorter PCL chains showing slower crystallization rates and leading to higher aspect ratio platelets, in agreement with a previous study by Xia et al. ^25^. Despite these variations in platelet dimensions, the lamellar thickness remained similar for PCL chain lengths above PCL_25_ (Fig. 1C). Conversely, PCL_25_ exhibited noticeably thicker crystalline lamellae (10.5 ± 0.4 nm). We ascribed this behavior to the shorter chain, which folds once when crystallizing, contributing to a relatively longer crystalline stem and yielding a thicker lamellar crystal compared to PCL_45_, PCL_75_, and PCL_90_, whose chains fold 3, 5, and 7 times, respectively, during crystallization^38^. These observations suggest that for PCL chain lengths above 25, lamellar thickness is mainly directed by crystallization temperature rather than polymer chain length.

NanoDSC is well-suited for probing the melting temperatures of solution-grown crystalline platelets, as it provides highly sensitive thermal analysis capable of detecting subtle transitions in dilute nanostructured assemblies. To this end, the solution melting temperatures (*T*_m(s)_) of platelets crystallized from PCL at 20 °C were measured by nanoDSC. A scan rate of 1 °C min^−1^ was found to be optimal, as slower rates (0.5 °C min^−1^) produced less distinct melting peaks, while faster rates (2 °C min^−1^) resulted in broader melting peaks (Supplementary Fig. 10). Our results showed an increase in *T*_m(s)_ with increasing PCL chain length, ranging from 35.7 °C for PCL_45_ to 38.5 °C for PCL_90_ (Fig. 1D). Integration of the nanoDSC melting peaks enabled us to determine the solution melting enthalpy (Δ*H*_m(s)_) and the corresponding degree of crystallinity (*X*_c(s)_) of the self-assembled platelets, which increased with increasing PCL chain length, from 48% for PCL_25_ to 94% for PCL_90_ (Supplementary Table 1). To compare this behavior with bulk polymers, differential scanning calorimetry (DSC) was also used to determine Δ*H*_m(DSC)_ and *X*_c(DSC)_ (Supplementary Fig. 11). Interestingly, the opposite trend was observed in this case, with crystallinity decreasing with increasing PCL chain length, ranging from 63% for PCL_25_ to 51% for PCL_90_ (Supplementary Table 1, Supplementary Fig. 12). This difference between bulk and self-assembled materials reflects that bulk crystallization is primarily limited by chain mobility, whereas crystallization within platelets occurs under confined conditions that promote more ordered packing of longer PCL chains. To further confirm these observations, wide-angle X-ray scattering (WAXS) measurements were performed on both the bulk polymers and self-assembled platelets (Supplementary Fig. 13), again showing decreasing crystallinity with increasing chain length in the bulk polymers and increasing crystallinity in the self-assembled materials (Supplementary Table 2 and Supplementary Fig. 14). Finally, selected area electron diffraction (SAED) radial profile analysis of platelets provided further confirmation of the increasing crystallinity trend observed by nanoDSC and WAXS (Supplementary Fig. 15 and Supplementary Table 3). Accordingly, the increase in melting temperature of solution-grown platelets is attributed to higher crystallinity and improved crystalline order of the self-assembled platelets at longer PCL chain lengths, together with reduced chain-end interactions, which may disrupt crystal packing as PCL chain length increases^39^. Notably, platelets made from PCL_25_ deviate from the general trend, with a melting temperature of 37.1 °C. This was slightly higher than platelets made from PCL_45_ (35.7 °C), but still lower than PCL_75_ (37.7 °C) and PCL_90_ (38.5 °C). These results can be rationalized by considering that platelets formed from PCL_25_ possess comparatively thicker lamellae, which translates into an increased melting temperature; however, this physical contribution is ultimately less significant than the molecular confinement increase in thermal stability observed for lamellae containing longer polymer chains (PCL_75_ and PCL_90_). Furthermore, platelets made from PCL_75_ and PCL_90_ exhibit two distinct melting peaks, a behavior that we attribute to a melting-recrystallization-remelting sequence during the nanoDSC heating scan. At the standard employed scan rate (1 °C min^−1^), chain mobility is sufficient for partially melted, metastable lamellae to reorganize by chain diffusion and anneal into more thermodynamically stable, thicker lamellae. Consequently, the lower-temperature melting peak (37.7 °C for PCL_75_ and 38.5 °C for PCL_90_) corresponds to the melting of the original, less stable lamellar population, while the second, higher-temperature peak (*T*_m2(s)_), 40.0 °C for PCL_75_ and 40.7 °C for PCL_90_) reflects remelting of the annealed, thickened lamellae formed in situ (Fig. 1D). This phenomenon is more prominent for higher molar-mass samples, as the increased chain length and entanglement promote enhanced reorganization and recrystallization during heating. This interpretation is consistent with fractional melting and recrystallization observed in crystalline polymers driven by kinetics and thermodynamic stabilization^40^.

### Effect of lamellar thickness on melting temperature

After elucidating the influence of PCL chain length on the melting temperature of the 2D PCL platelets, we sought to investigate a second key parameter, lamellar thickness, which plays a central role in determining melting behavior. According to the Gibbs-Thomson equation, the melting temperature of a single polymer crystal decreases as its lamellar thickness decreases, reflecting the higher surface-to-volume ratio and resulting increase in surface free energy (σₑ) associated with a thinner lamella (Supplementary Equation (1)). Furthermore, because lamellar thickness is largely established during crystallization, the Hoffman-Weeks interpretation provides the thermodynamic link between crystallization temperature, lamellar thickness, and the corresponding melting temperature (Supplementary Equation (2)). From this, it follows that lower crystallization temperatures promote the formation of thinner lamellae.

**Fig. 2:**
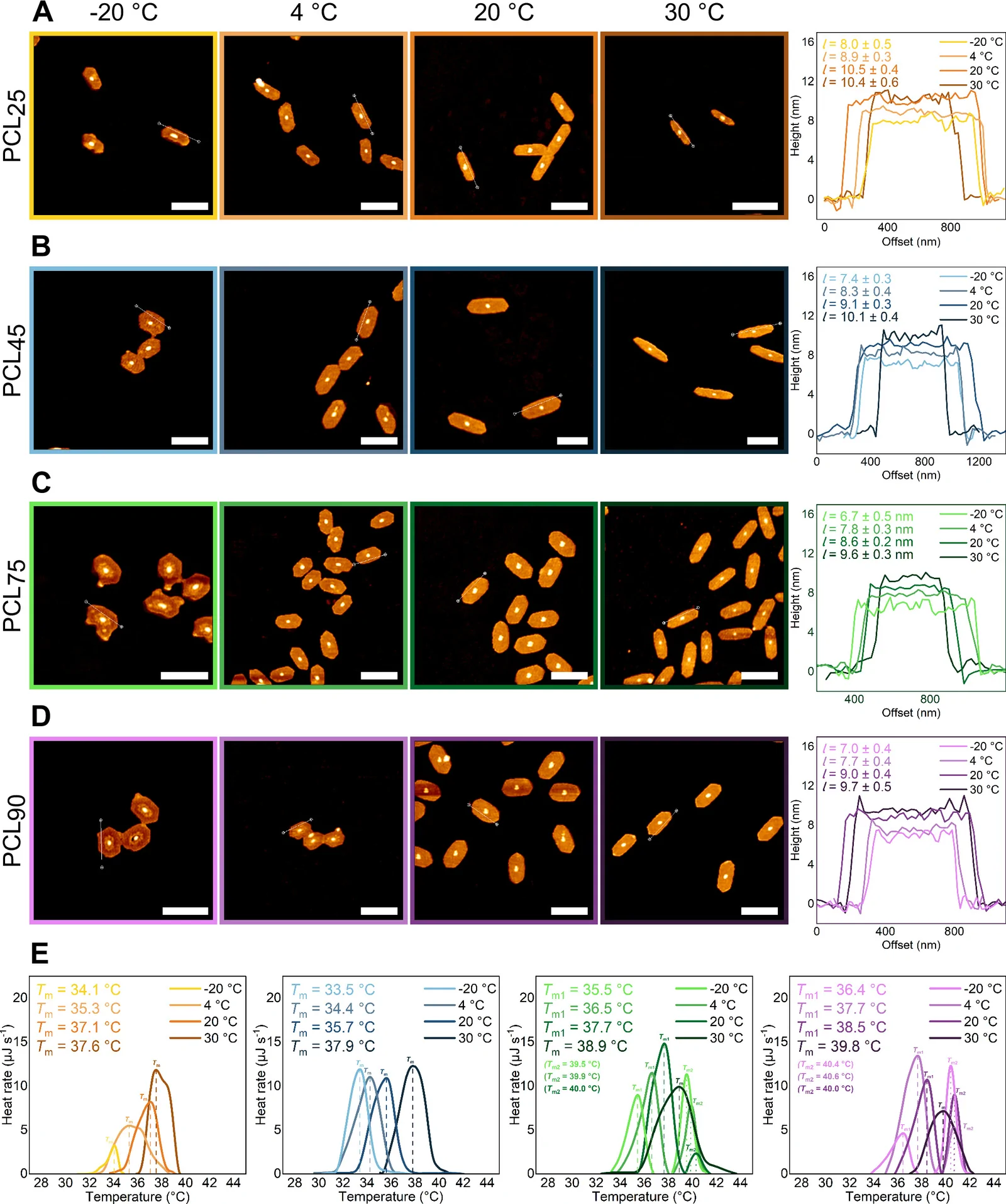
Formation and melting of 2D PCL platelets with varying PCL lengths at different crystallization temperatures. AFM images and corresponding stacked AFM height profiles of 2D platelets made from **A** PCL_25_, **B** PCL_45_, **C** PCL_75_, and **D** PCL_90_ formed at varying crystallization temperatures: −20, 4, 20, and 30 °C (left to right). All scale bars are equivalent to 1 µm. **E** Plot of heat rate against temperature extracted from nanoDSC depicting the melting temperatures of 2D platelets comprising PCL_25_, PCL_45_, PCL_75_, and PCL_90_ (left to right) formed at varying crystallization temperatures.

Solutions of PCL homopolymers of differing chain lengths were added to separate seed solutions maintained at four crystallization temperatures (*T*_c_*, −20 °C, 4 °C, 20 °C, and 30 °C). The resulting 2D platelets were subsequently analyzed by TEM and AFM to evaluate their lateral dimensions and height profiles (Fig. 2A–D and Supplementary Fig. 16). Across all PCL chain lengths, a systematic decrease in crystallization temperature produced a distinctive and consistent reduction in lamellar thickness (Supplementary Table 4). Platelets formed at different crystallization temperatures were analyzed by nanoDSC to determine their melting temperatures (Fig. 2E). As expected, platelets with thinner lamellae exhibited lower melting temperatures than those with thicker lamellae, consistent with the Gibbs-Thomson relation. This ranged from 33.5 °C (lowest) for platelets made from PCL_45_ at −20 °C to 39.8 °C (highest, excluding all *T*_m2(s)_ values) for platelets made from PCL_90_ at 30 °C. Solution equilibrium melting temperatures (*T*_m_^0^_(s)_) were obtained using the Hoffman-Weeks analysis, where melting temperatures (*T*_m(s)_) are plotted against crystallization temperatures (*T*_c_^*^) and extrapolated to the condition *T*_m(s)_ = *T*_c_^*^ (Supplementary Fig. 17 and Supplementary Table 4). Notably, the resulting *T*_m_^0^_(s)_ values are systematically lower than bulk PCL values (Supplementary Fig. 18), reflecting both finite lamellar thickness of the solution-grown platelets and the presence of a surrounding ethanol medium. While the Gibbs-Thomson analysis accounts for lamellar thickness, the experimentally determined values implicitly include additional contributions from the solvent-polymer interactions, thereby modifying the interfacial free energy contribution and leading to a lower equilibrium melting temperature compared to bulk PCL^41^. These *T*_m_^0^_(s)_ values were subsequently used to calculate effective fold surface free energies via the Gibbs-Thomson analysis (Supplementary Fig. 19, Supplementary Table 4). Gibbs-Thomson analysis reveals a clear molecular weight dependence of the fold surface free energy. Shorter PCL chains (PCL_25_ and PCL_45_) exhibit higher σₑ values (~28–30 mJ m^-2^), whereas longer chains (PCL_75_ and PCL_90_) show significantly lower σₑ values (~18 mJ m^-2^). This inverse correlation indicates that shorter chains incur a greater energetic penalty at the fold surface, likely owing to their higher chain-end repulsion and reduced conformational regularity. In contrast, increasing chain length reduces the relative contribution of fold surface energetics, resulting in more thermodynamically stable lamellae. Consequently, the experimentally determined *T*_m_^0^_(s)_ and σₑ values should be regarded as apparent parameters that account for the thermodynamic influence of the solvent environment. Integration of the nanoDSC melting peaks and determination of the degree of crystallinity showed that crystallinity increased with increasing crystallization temperature for all PCL chain lengths except PCL_90_ (Supplementary Table 5 and Supplementary Fig. 20). This increase can be attributed to greater chain mobility at higher crystallization temperatures, allowing chains to reorganize and pack into more ordered crystalline structures. In contrast, PCL_90_ showed a slight reduction in crystallinity from 4 to 30 °C. As the PCL chain length exceeds the entanglement molar mass, this behavior may be attributed to increased chain entanglements, which can hinder crystal growth^39^. This interpretation is consistent with the higher melting temperature observed for PCL_90_ crystallized at 30 °C compared with PCL_75_, despite its lower degree of crystallinity, suggesting that the crystals formed are thermally more stable, potentially as a result of confinement effects.

Interestingly, reducing the crystallization temperature yielded platelets with a reduced aspect ratio (Supplementary Fig. 21). This behavior arises from increased supercooling at lower crystallization temperatures (${T}_{m({{{\rm{s}}}})}^{0}$ - *T*_c_^*^), which accelerates primary and secondary nucleation rates while restricting chain mobility, thereby suppressing anisotropic lateral growth. As a result, crystal growth becomes more isotropic, yielding smaller, less elongated platelets^42^.

Finally, to investigate whether the presence of a corona block affects platelet melting temperatures, we synthesized a series of PCL_90_*-b-*PDMA_x_ diblock copolymers (where x = 42, 150, and 308) (Supplementary Figs. 22–25 and Supplementary Table 6). PDMA was chosen as a well-established, biocompatible corona block known to support efficient CDSA^35–37^. NanoDSC analysis of platelets assembled from these diblock copolymers showed no measurable change in melting temperature with increasing corona block length (Supplementary Fig. 26), indicating that the melting behavior of solution-grown 2D platelets is governed solely by the core-forming PCL block.

### Selective thermal disassembly

After identifying the critical parameters that impact the melting temperature of 2D PCL platelets, we sought a structural way to demonstrate thermally driven manipulations in situ. We hypothesized that by constructing bilayer platelets with different polymer chain lengths and heterogeneous lamellar thicknesses, we could exploit the contrasting thermal stabilities to selectively melt the thinner inner layer without disturbing the more stable, thicker perimeter lamellae.

**Fig. 3:**
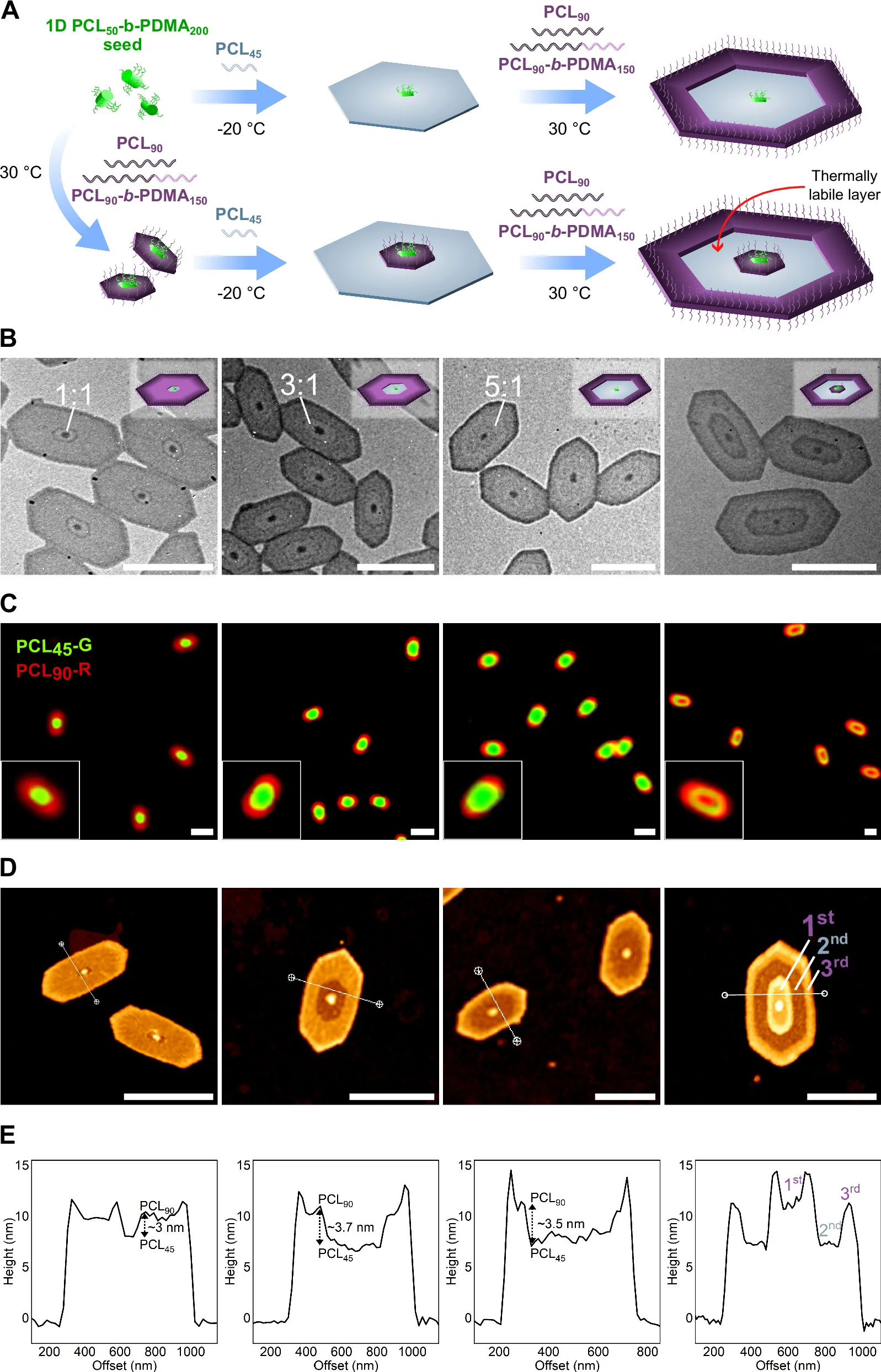
Formation of bilayer and trilayer platelets with contrasting thermal stabilities across layers. **A** Schematic of the route taken to form bilayer and trilayer platelets with contrasting thermal stabilities and heterogeneous lamellar thicknesses; to a solution of 1D PCL_50_*-b-*PDMA_200_ seed, either PCL_45_ at −20 °C (bilayer) or PCL_90_/PCL_90_*-b-*PDMA_150_ at 30 °C (trilayer) was added, followed by the subsequent addition of either PCL_90_/PCL_90_*-b-*PDMA_150_ at 30 °C (bilayer) or PCL_45_ at −20 °C (trilayer), and finally the addition of a second PCL_90_/PCL_90_*-b-*PDMA_150_ layer at 30 °C (trilayer). **B** TEM micrographs, **C** confocal fluorescence microscopy images, and **D** AFM images of bilayer and trilayer platelets formed with increasing (from 1:1 to 5:1) unimer:seed ratios (left to right). All scale bars are equivalent to 1 µm. **E** Height profiles extracted from AFM images.

To test this concept, single-layer 2D platelets were first prepared under the most thermally labile conditions, using PCL_45_ homopolymer crystallized at *T*_c_^*^ = −20 °C with unimer-to-seed ratios of 1:1, 3:1, and 5:1. In all cases, epitaxial growth proceeded cleanly, yielding platelets with uniform morphology and no observable aggregation (Supplementary Fig. 27). These platelets were subsequently employed as seeds for epitaxial growth of a thermally stable outer layer composed of a PCL_90_/PCL_90_*-b-*PDMA_150_ unimer mixture (1:1 homopolymer-to-diblock ratio), crystallized at *T*_c_^*^ = 30 °C using a fixed unimer-to-seed ratio of 10:1 (Fig. 3A–E). Incorporation of the diblock copolymer was essential to maintain colloidal stability at elevated unimer-to-seed ratios, preventing aggregation during outer-layer growth, and resulting in bilayer platelets with uniform morphology (Supplementary Fig. 28). Across all three platelet populations, growth of the second layer resulted in a consistent lamellar thickness of 10–14 nm, with height increase localized at the platelet edges (Fig. 3E). This behavior was attributed to differences in crystallization kinetics between PCL_90_ homopolymer and the corresponding diblock copolymer, with the homopolymer crystallizing more rapidly^25^. While the unimer-to-seed ratio for the outer layer was held constant, that of the inner layer was systematically varied to modulate the surface area of the hollow region generated upon subsequent thermal treatment. The resulting bilayer platelets thus comprised a thin, thermally labile inner lamella surrounded by a thicker, thermally stable outer framework, with a difference between the lamellar thicknesses ranging from 3.0 to 3.7 nm (Fig. 3E). Importantly, this modular growth strategy establishes a general platform for programming lamellar thermal stability across multiple layers, providing a direct pathway toward more complex multilayer architectures in which selective melting can be spatially encoded.

To further demonstrate the versatility of this approach, ABA-type trilayer platelets were prepared in which the first and third layers were grown from PCL_90_/PCL_90_*-b-*PDMA_150_ unimers (5:1 unimer-to-seed ratio), while the intervening thermally labile layer was composed of PCL_45_ (5:1 unimer-to-seed ratio) (Fig. 3E). AFM analysis revealed that the inner PCL_90_/PCL_90_*-b-*PDMA_150_ layer exhibited a slightly greater height (12–15 nm) than the outer PCL_90_/PCL_90_*-b-*PDMA_150_ (11-12 nm). We attribute this difference to crystallization of the first layer directly onto the original seed facets, whereas growth of the third layer occurs on the thinner, reconstituted facets of the intermediate PCL_45_ lamella, resulting in a reduced effective lamellar thickness. To confirm that selective melting was confined to the PCL_45_ lamella, PCL_45_ and PCL_90_ homopolymers were tagged with spectrally distinct fluorescent probes, and platelets were prepared from these modified polymers. End-group functionalization was achieved via esterification with carboxylated BODIPY dyes, yielding PCL_45_-BODIPY-FL (green) and PCL_90_-BODIPY 630/650 (red), with SEC analysis confirming low dispersity, comparable to the parent materials (Supplementary Figs. 29, 30)^15^. Confocal fluorescence microscopy of the resulting platelets revealed spatially resolved emission from each polymer, directly visualizing the layered architecture and confirming clear segregation of the thermally labile and thermally stable lamellae within individual platelets (Fig. 3C and Supplementary Fig. 31).

Leveraging the thermal window between PCL_45_ and PCL_90_, we sought to selectively melt the inner lamella prior to the onset of melting of the outer lamella. NanoDSC measurements indicated that the temperature range of 35–38 °C covered the majority of the PCL_45_ melting transition while minimally overlapping with that of PCL_90_. Accordingly, bilayer platelets were subjected to controlled heating between 35 and 40 °C for 1 min (Supplementary Fig. 32), a heating time chosen to match the effective thermal exposure experienced during nanoDSC scans. The upper temperature limit of 40 °C was chosen to encompass both melting peaks and to observe the onset of melting-induced structural changes in the more thermally stable crystalline layer.

At 35 and 36 °C, hollow platelets were observed only in a minor fraction of the population (8% and 17%, respectively), with most platelets remaining unchanged. Heating at 38 °C increased the proportion of hollow platelets to 61%; however, the remaining platelets exhibited partial dissolution of the outer perimeter, indicating the onset of thermal damage to the thicker lamella (Supplementary Fig. 32). At 40 °C, complete structural failure was observed for all platelets. On this basis, 37 °C was identified as the optimal compromise, yielding 48% hollow platelets while preserving the remaining population without detectable damage.

To further increase the yield of hollow platelets while maintaining structural integrity, heating time was varied at 37 °C from 1 to 5 min (Supplementary Fig. 33). An optimal heating window of 3 min was selected, producing 89% of hollow platelets with only 2% exhibiting structural failure and the remaining 9% retaining their original double-layer morphology. Under these conditions, the inner lamella melted selectively while the outer lamella remained intact, enabling reproducible formation of hollow 2D platelets with fully preserved crystalline perimeters.

**Fig. 4:**
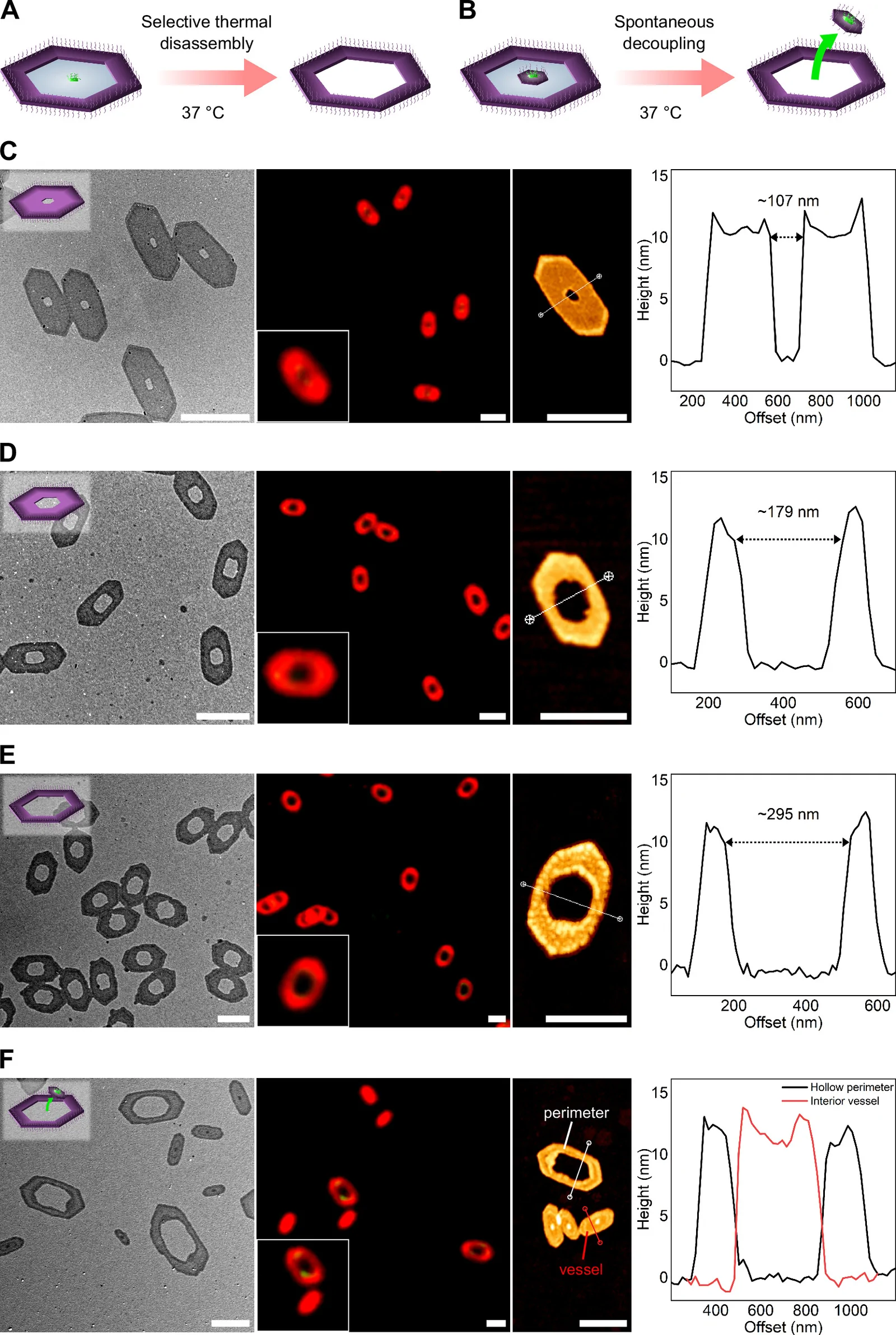
Formation of hollow and hollow/vessel decoupling platelets. **A** Schematic of the route taken to form hollow platelets of differing hollow core areas and **B** architecturally decoupling platelets. TEM micrographs, confocal fluorescence images, and AFM images of 2D platelets prepared using **C** 1:1, **D** 3:1, and **E** 5:1 unimer-to-seed ratio of the thermally labile layer. **F** TEM micrograph, confocal fluorescence image, and AFM image of triple-layer platelet solutions heated at 37 °C for 3 min. All scale bars are equivalent to 1 µm. Height profiles were extracted from AFM images.

Bilayer and trilayer platelets of varying dimensions were subsequently heated at 37 °C for 3 min to induce selective melting of the targeted lamellae and analyzed by TEM and AFM (Fig. 4A–F). The fluorescently labeled analogs were imaged by confocal fluorescence microscopy (Fig. 4C–F). For bilayer platelets, the width of the hollow interior systematically increased from 107 ± 14 nm at a 1:1 unimer-to-seed ratio to 179 ± 15 nm at 3:1, and 295 ± 24 nm at 5:1 (Supplementary Fig. 34). Surface area also reflected the same trend (Supplementary Fig. 34) and was consistent with the lateral dimensions of single-layer PCL_45_ platelet seeds. AFM height profiles showed the complete absence of features corresponding to a 7–8 nm lamella, indicating full melting of the PCL_45_ layer (Fig. 4C–E), which was confirmed by confocal microscopy showing retention of only the PCL_90_ (red) component within the platelet architecture (Fig. 4C–E).

For trilayer platelets, selective melting of the intermediate PCL_45_ layer resulted in spontaneous architectural decoupling into two discrete crystalline entities: a released inner vessel and a surrounding hollow perimeter, both composed of PCL_90_/PCL_90_*-b-*PDMA_150_ (Fig. 4F). Confocal microscopy revealed minimal residual PCL_45_ signal, attributed to limited recrystallization during sample preparation, which is consistent with the localized lamellar thickening observed by AFM at the inner perimeter of the hollow platelets and the elevated effective crystallization temperature (*T*_c_^*^ = 37 °C).

Notably, this thermally programmed process enables the rapid and reproducible generation of uniform hollow and decoupled 2D architectures under mild conditions and on minute timescales, in contrast to previously reported strategies that rely on chemical degradation (*e.g*., accelerated hydrolysis of PCL) and prolonged ageing at sub-zero temperatures^33,34^.

### Thermal reconstruction: from hollow to whole platelets

**Fig. 5:**
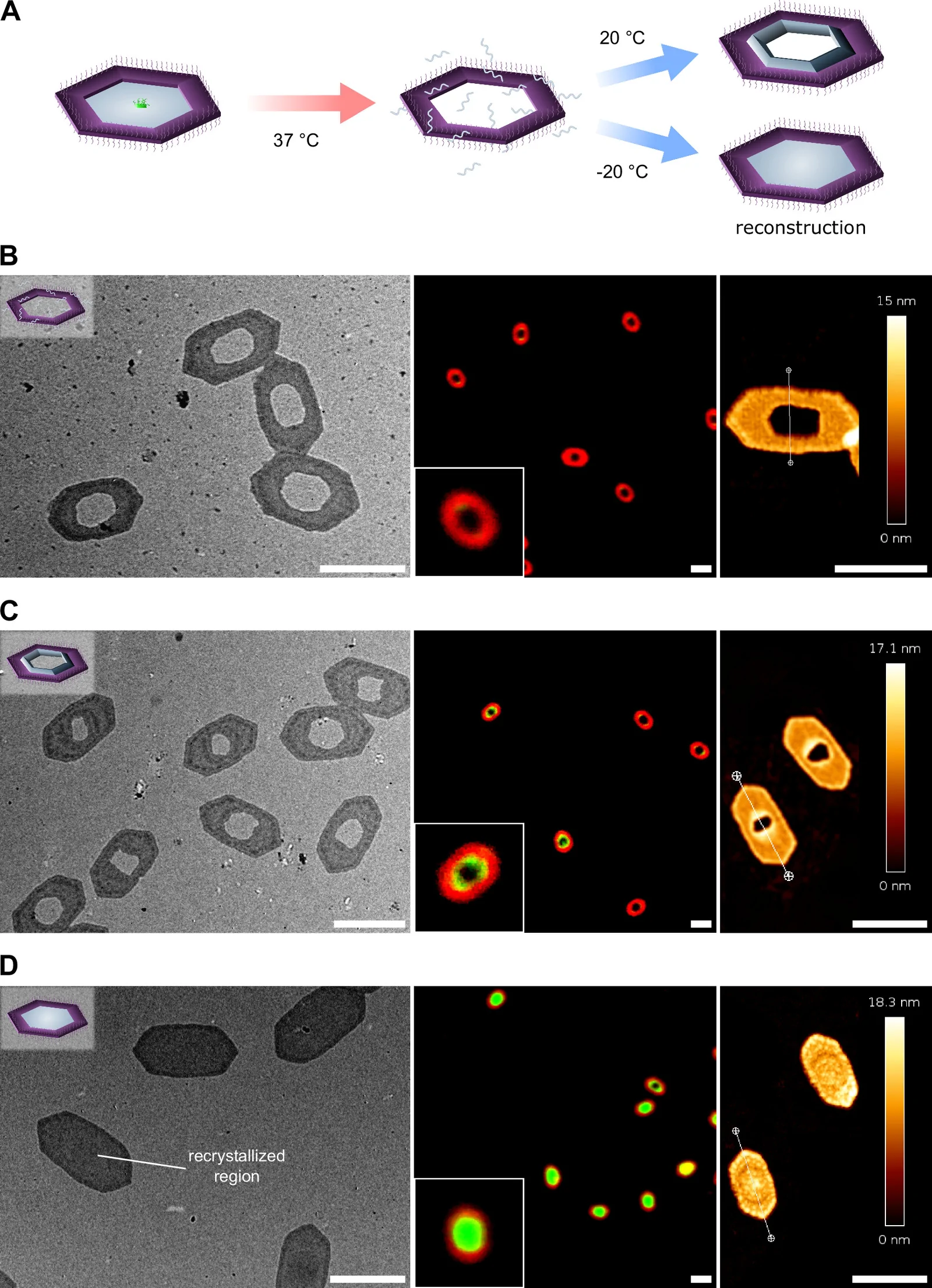
Temperature-controlled recrystallization of hollow platelets. **A** Schematic depicting the routes of recrystallization taken to form spatially confined recrystallized platelets and self-healed full platelets. **B** TEM micrograph, confocal fluorescence microscopy image, and AFM image of hollow platelets. **C** TEM micrograph, confocal fluorescence microscopy image, and AFM image of hollow platelets (shown in **B**) cooled to 20 °C to form patchy hollow platelets. **D** TEM micrograph, confocal fluorescence microscopy image, and AFM image of hollow platelets (shown in **B**) cooled to −20 °C to reform full platelets. All scale bars are equivalent to 1 µm.

To probe whether thermally induced disassembly could be reversible, we next examined the structural evolution of hollow platelets upon cooling. Hollow platelets were imaged immediately after selective melting at 37 °C while remaining dispersed in ethanol, a favorable solvent for PCL crystallization. Under these conditions, melting of the inner lamella disrupts crystalline order without chemically altering the polymer chains, which remain available for recrystallization upon cooling (Fig. 5A). When the system was cooled from 37 °C to 20 °C without stirring, PCL chains recrystallized preferentially within the hollow interior rather than along the outer perimeter, leading to rapid formation of a thickened lamellar domain confined to the hollow center (Supplementary Fig. 35). This behavior is consistent with the recrystallization process occurring at a higher temperature than the initial growth conditions, where reduced supercooling promotes slower chain folding and increased lamellar thickness. Rather than covering the entirety of the hollow center, the PCL chains originating from the thin inner layer crystallize in a localized, thickened patch, likely as a consequence of several chains stacking together rather than contributing to lateral growth (Fig. 5B). Notably, the recrystallizable chain pool is not derived purely from the thermally labile layer: the exposed seeds also melt at 37 °C (*T*_m(s)_ = 32.4 °C, Supplementary Fig. 36), while PCL released from platelets that are subjected to structural failure during the melting process provides an additional source. Interestingly, the double-layer platelets could be transferred into water, and selective melting of the inner layer could be achieved at 50 °C (Supplementary Fig. 37). However, the solvent change prevented subsequent recrystallization, and the released PCL chains reorganized into spherical amorphous particles instead of recrystallizing (Supplementary Fig. 37).

The spatially confined recrystallization was directly confirmed using BODIPY-labeled platelets, which revealed fluorescence exclusively within the hollow interior following cooling to 20 °C, with no detectable signal at the outer perimeter (Fig. 5B–D). Lowering the recrystallization temperature to −20 °C led to an increase in fluorescence intensity within the hollow region, indicating enhanced lateral stretch under conditions of increased supercooling (Fig. 5C). Under these conditions, the free chains preferentially reassembled along the interior facets of the hollow platelet, promoting lateral growth and effectively restoring the original platelet architecture. Following recrystallization at −20 °C, 74% of platelets underwent complete reconstruction, whereas 22% showed only partial reconstruction, resulting in patchy platelets (Supplementary Fig. 38). The remaining 4% of the platelet population showed no observable change post-melting and reconstruction, retaining their original double-layer morphology. Nevertheless, this process enabled self-healing of hollow platelets without the introduction of external polymer unimers, demonstrating that recrystallization pathways can be redirected solely through thermal control. This work demonstrates temperature-programmed, reversible structural reconfiguration of CDSA-derived 2D platelets in solution, establishing melting and recrystallization as direct mechanistic pathways for dynamically restructuring crystalline polymer architectures without chemical modification or external templating.

To investigate the phenomenon of preferential recrystallization within the hollow interior, a series of competition experiments was conducted. To emulate the distinct environments of the exterior and interior of hollow platelets, we synthesized PCL_90_ homopolymer platelets (hereafter HP platelet seeds), representing the crystalline interior, and PCL_90_/PCL_90_-*b*-PDMA_150_ platelets (hereafter BCP platelet seeds) representing the exterior incorporating a PDMA corona concentrated at the platelet perimeter (Supplementary Fig. 39). As a control experiment, 10 µL of 10 mg mL^-1^ PCL_45_ unimer solution was added to a 1:1 mixture of HP and BCP platelet seeds at crystallization temperatures of 30, 20, 4, and −20 °C. Interestingly, a subtle increase in preferential deposition on HP seeds was observed with decreasing temperature, increasing from 50% at 30 °C to 57% at -20 °C (Supplementary Fig. 39). This trend can be rationalized by differences in interfacial accessibility between the two platelet seed types, arising from the crystal-edge localized PDMA corona on BCP seeds, which modifies the effective interface available for chain deposition. The preference towards HP seeds at lower temperatures indicates that crystallization becomes increasingly sensitive to interfacial accessibility under conditions of greater kinetic constraint, leading PCL to crystallize at the more accessible interface.

To more directly mimic the recrystallization process happening in hollow platelets, a thermally labile outer layer was grown on HP platelet seeds by adding 10 µL of 10 mg mL^-1^ PCL_45_ at -20 °C, thereby creating inverted hollow platelet analogs (Supplementary Fig. 40). Recrystallization behavior was then investigated by heating the samples at 37 °C for 3 min to selectively dissolve the outer PCL_45_ layer, followed by cooling to -20 °C. This experiment was conducted under static and stirring conditions during the recrystallization process. Comparing these conditions allowed assessment of whether the local enrichment of polymer chains near the platelet seeds influences the subsequent recrystallization. Under static conditions, the model exhibited results resembling those of the hollow platelets, with 95% of the recrystallized PCL_45_ deposited on HP seeds. Interestingly, even under stirring, a preference for HP seeds remained, with 72% of chains crystallizing back on the original entity (Supplementary Fig. 40). This behavior may be ascribed, in part, to the spatial confinement of chains following selective melting, such that recrystallization preferentially occurs in the immediate vicinity of the HP seeds rather than on more distant BCP seeds. However, this preference remains even under stirring, suggesting that local concentration effects alone cannot fully account for this phenomenon. Instead, these results are also consistent with a solution-phase analog of the polymer melt-memory effect, in which partially disrupted crystalline domains and surviving subcritical nuclei can template subsequent recrystallization^43^. An analogous experiment was also performed to further consolidate these findings using double-layer BCP seeds, which yielded a similar preference for self-recrystallization, with 96% deposition under static conditions and 60% under stirring (Supplementary Fig. 41). The reduced bias relative to HP seeds under stirred conditions suggests that the PDMA corona partially suppresses templated recrystallization; however, this effect appears secondary to the templating influence arising from surviving subcritical nuclei. Together, these findings are consistent with the behavior observed in hollow and spontaneously decoupling platelets (Supplementary Fig. 42). More broadly, these observations demonstrate that hollow platelet architectures generated by selective melting are not end states but can be reversibly reconfigured in solution through simple thermal modulation. This reversibility suggests a platform in which polymer chains can be released into solution, reassigned spatially, and subsequently reincorporated into defined nanostructures without chemical modification or additional templating agents.

## Discussion

In this work, we demonstrate that the melting behavior of solution-grown 2D polymer crystals encodes rich structural information that is inaccessible from bulk measurements and can be exploited as an active design parameter in CDSA. Through systematic nanoDSC analysis of PCL 2D platelets, we disentangle the respective roles of polymer chain length and lamellar thickness in governing melting temperatures and establish quantitative thickness-melting relationships consistent with classical polymer crystallization thermodynamics, translated here to finite, solution-grown crystals.

By leveraging controlled differences in thermal stability across epitaxially grown lamellae, we show that selective melting enables spatially resolved disassembly of multilayer platelets under mild conditions, yielding uniform hollow architectures with preserved crystalline perimeters. Extension of this strategy to trilayer platelets further demonstrates that thermal stability gradients can be programmed to induce spontaneous architectural decoupling into discrete crystalline entities. Importantly, these hollow structures are not static end states; rather, polymer chains can be reversibly reincorporated into the platelet framework through temperature-controlled recrystallization, enabling confined regrowth or self-healing without chemical modification or external seeds.

Together, these findings position melting temperature as both a structurally sensitive descriptor and a functional control parameter in CDSA, bridging polymer crystallization thermodynamics with nanoscale self-assembly. More broadly, the thermally reversible and reconfigurable nature of these 2D platelet architectures provides a route towards polymer assemblies with switchable structural and functional states. For example, selective melting could expose encapsulated catalytic species, enabling on-demand functionality, followed by their recapture within the crystalline network upon cooling. Such behavior can enable catalyst recycling and separation from reaction products. More generally, these findings establish a framework for designing thermally reconfigurable crystalline polymer nanostructures in which function can be programmed through temperature alone.

## Methods

### Synthesis of 2-cyano-5-hydroxypentan-2-yl ethyl carbonotrithioate (CHPET)

Sodium ethanethiolate (5 g, 59.4 mmol, 1 equiv.) was dissolved in dry diethyl ether (300 mL) and cooled to 0 °C before carbon disulfide (3.87 mL, 65.5 mmol, 1.1 equiv.) was added dropwise. The reaction was stirred at room temperature for 2 h before the addition of iodine (7.55 g, 29.7 mmol, 0.5 equiv.), and then stirred for a further 2 h to yield bis-(ethylsulfanylthiocarbonyl) disulfide. Following aqueous work-up, the organic phase was dried over MgSO_4_, filtered, and concentrated. The intermediate was subsequently reacted with 4,4’-azobis(4-cyanovaleric acid) (ACVA, 11.95 g, 42.6 mmol) in ethyl acetate (250 mL) at 80 °C overnight under a N_2_ atmosphere. After removal of the volatile solvent *in vacuo*, purification by silica gel column chromatography (hexane: dichloromethane = 1:3) afforded 4-cyano-4-(((ethylthio) carbonothioyl)thio)pentanoic acid (CEPA) as a yellow solid.

CEPA (7.00 g, 26.7 mmol, 1 equiv.) was subsequently dissolved in dry THF (250 mL) and cooled to -78 °C under a N_2_ atmosphere. A 1 M solution of borane tetrahydrofuran complex (28.15 mL, 28.15 mmol, 1 equiv.) was added dropwise over 20 min, and the reaction mixture was left to stir for 1 h at -78 °C before warming to room temperature and stirring overnight under a N_2_ atmosphere. The reaction was quenched by the gradual addition of methanol (50 mL), concentrated *in vacuo*, and then dissolved in diethyl ether (150 mL). After aqueous work-up, the organic layer was dried over MgSO_4_, filtered, and concentrated. Purification was carried out using silica gel column chromatography (hexane: ethyl acetate = 1:1), affording the dual-head chain-transfer agent (CTA) 2-cyano-5-hydroxypentan-2-yl ethyl carbonotrithioate (CHPET) as an orange-red oil (4.25 g, 17.1 mmol, 64%).

### Synthesis of poly(ɛ-caprolactone) homopolymers

Inside a nitrogen-filled glovebox maintained at oxygen and moisture levels below 0.1 ppm, a solution of diphenyl phosphate (DPP, 100 mg, 0.460 mmol, 1 equiv.) in dry toluene (3 mL) and a solution of the dual-head CTA, 2-cyano-5-hydroxypentan-2-yl ethyl carbonotrithioate (CHPET, 100 mg, 0.460 mmol, 1 equiv.) in dry toluene (4 mL) were sequentially introduced to ɛ-caprolactone (1.05 g, 9.20 mmol, 20 equiv. to synthesize PCL_15_) dissolved in dry toluene (4 mL). The reaction mixture was stirred at room temperature for 5 h. Following polymerization, the reaction solution was removed from the glovebox and precipitated dropwise into cold diethyl ether three times. The resulting polymer was isolated by centrifugation and dried under vacuum. The purified material was subsequently characterized by ^1^H NMR spectroscopy and SEC analysis. ɛ-caprolactone amounts were varied depending on the targeted degree of polymerization (DP): (1.84 g, 16.1 mmol, 35 equiv. to synthesize PCL_25_), (3.21 g, 28.1 mmol, 61 equiv. to synthesize PCL_45_), (4.73 g, 41.4 mmol, 90 equiv. to synthesize PCL_75_), (6.30 g, 55.2 mmol, 120 equiv. to synthesize PCL_90_).

### Synthesis of PCL_90_-*b*-poly(*N,N*-dimethylacrylamide)_x_ diblock copolymers

PCL_90_ (100 mg, 0.00952 mmol, 1 equiv.), DMA (70.8 mg, 0.714 mmol, 75 equiv. for DP = 42; or 189 mg, 1.904 mmol, 200 equiv. for DP = 150; or 330 mg, 3.33 mmol, 350 equiv. for DP = 308), and AIBN (0.19 mg, 0.0011 mmol, 0.12 equiv.) were dissolved in 1,4-dioxane (1 mL) and transferred into an ampoule. The reaction mixture was subjected to three freeze-pump-thaw cycles to remove dissolved oxygen, then heated at 70 °C for 2 h. Polymerization was terminated by immersing the ampoule in liquid nitrogen. The crude polymer was precipitated dropwise into ice-cold diethyl ether three times, isolated, and dried under vacuum. The final product was characterized by ^1^H NMR spectroscopy and SEC analysis.

### Synthesis of fluorescently functionalized polymers

Two different carboxylic acid-functionalized BODIPY dyes (BODIPY 630/650 and BODIPY FL) were employed to functionalize two distinct PCL chain lengths. PCL_45_ (100 mg, 0.0185 mmol, 2 equiv.), BODIPY-FL carboxylic acid (2.70 mg, 0.00926 mmol, 1 equiv.), 4-dimethylaminopyridine (DMAP, 1.13 mg, 0.00926 mmol, 1 equiv.), and *N,N*′-dicyclohexylcarbodiimide (DCC, 19.1 mg, 0.0926 mmol, 10 equiv.) or PCL_90_ (100 mg, 0.00952 mmol, 2 equiv.), BODIPY-630/650 carboxylic acid (2.14 mg, 0.00476 mmol, 1 equiv.), 4-dimethylaminopyridine (DMAP, 0.582 mg, 0.00476 mmol, 1 equiv.), and *N,N*′-dicyclohexylcarbodiimide (DCC, 9.82 mg, 0.0476 mmol, 10 equiv.) were dissolved in dichloromethane (DCM, 2 mL) in an ampoule. The reaction mixture was stirred at room temperature for 3 days to allow coupling. After completion, the reaction mixture was filtered, and the filtrate was precipitated dropwise into diethyl ether three times. The resulting end-functionalized fluorescently labeled polymers were collected and dried under vacuum.

### Preparation of flash-frozen seeds in a flow reactor

Flash-frozen PCL_50_-*b*-PDMA_200_ seeds were prepared in a flow reactor setup as described in a previous literature report^28^. A high-performance liquid chromatography pump (Jasco PU-980) was coupled to a stainless-steel coil (inner diameter 0.75 mm, total volume 2 mL) positioned in an oil bath maintained at 75 °C. Downstream of this heated section, polytetrafluoroethylene (PTFE) tubing (inner diameter 0.5 mm, total volume 2 mL) was installed. Within this segment, a winding element (0.5 mL) and a cooling coil (0.5 mL) were held at -78 °C using a dry ice-acetone bath, while a subsequent equilibration coil (1 mL) was maintained at room temperature. A solution of the diblock copolymer (PCL_50_-*b*-PDMA_200_, 0.1 mg mL^-1^ in ethanol) was delivered using a high-performance liquid chromatography (HPLC) pump at flow rates of 1, 2, and 4 mL min^-1^. Steady-state samples were collected after a total of 12 mL of polymer solution (corresponding to three reactor volumes) had passed through the system.

### Seeded growth of 2D platelets at different crystallization temperatures

PCL homopolymers (or BODIPY dye-modified polymers, or PCL/PCL-*b*-PDMA 1:1 unimer mixtures) were dissolved in chloroform at a concentration of 10 mg mL^-1^. The resulting solution was maintained at the target crystallization temperature (-20, 4, 20, or 30 °C) and added to a dispersion of crystalline seed micelles (0.01 mg mL^-1^ in ethanol) in a screw-cap vial kept at the same target temperature. For a typical living CDSA experiment, when the unimer-to-seed ratio was fixed at 5, 5 μL of PCL solution (10 mg mL^-1^ in chloroform) was introduced into 1 mL of seed solution (0.01 mg mL^-1^ in ethanol). Immediately after addition, the mixture was vigorously shaken for 15 s and then allowed to age at the target temperature for an additional 2 min. Different unimer-to-seed ratios were achieved by varying the volume of unimer solution added to the seed dispersion.

### Sequential growth of multilayer platelets

Bilayer and trilayer platelets were prepared by successive unimer addition to preformed platelet seeds under otherwise identical CDSA conditions. Each growth step was followed by ageing for 2 min. at the targeted crystallization temperature before further addition or characterization.

### Selective thermal disassembly and recrystallization

Selective thermal disassembly experiments were carried out by heating platelet dispersions at prescribed temperatures (usually 37 °C) for controlled durations (usually 3 min), followed by immediate structural characterization (TEM, AFM, or confocal fluorescence microscopy). Post-heating, the platelet solutions were aged at targeted recrystallization temperatures (20 or -20 °C) for 2 min. followed by structural characterization (TEM, AFM, or confocal fluorescence microscopy).

## Supplementary information

Supplementary Information

Transparent Peer Review file

## Data Availability

The data supporting the findings of this study are available within this article and its Supplementary Information as well as from the corresponding author upon request.

## References

[1] Qiu, H., Hudson, Z. M., Winnik, M. A. & Manners, I. Micelle assembly. Multidimensional hierarchical self-assembly of amphiphilic cylindrical block comicelles. *Science* **347**, 1329–1332 (2015). 10.1126/science.1261816 25792323

[2] Hudson, Z. M. et al. Tailored hierarchical micelle architectures using living crystallization-driven self-assembly in two dimensions. *Nat. Chem.* **6**, 893–898 (2014). 10.1038/nchem.2038 25242484

[3] Pearce, A. K., Wilks, T. R., Arno, M. C. & O’Reilly, R. K. Synthesis and applications of anisotropic nanoparticles with precisely defined dimensions. *Nat. Rev. Chem.* **5**, 21–45 (2021). 10.1038/s41570-020-00232-7 37118104

[4] Xia, T., Xiao, L., Xie, Y., Dove, A. P. & O’Reilly, R. K. Deciphering evolution, function, and observation of crystallization-driven self-assembly. *Chem. Rev.* **125**, 10105–10178 (2025). 10.1021/acs.chemrev.5c00298 PMC12616703 41082283

[5] Qiu, H. et al. Branched micelles by living crystallization-driven block copolymer self-assembly under kinetic control. *J. Am. Chem. Soc.* **137**, 2375–2385 (2015). 10.1021/ja5126808 25585041

[6] Finnegan, J. R. et al. Gradient crystallization-driven self-assembly: cylindrical micelles with “patchy” segmented coronas via the coassembly of linear and brush block copolymers. *J. Am. Chem. Soc.* **136**, 13835–13844 (2014). 10.1021/ja507121h 25243847

[7] Inam, M. et al. 1D vs. 2D shape selectivity in the crystallization-driven self-assembly of polylactide block copolymers. *Chem. Sci.* **8**, 4223–4230 (2017). 10.1039/c7sc00641a PMC5635812 29081959

[8] Street, S. T. G. et al. Precision stealth nanofibers via PET-RAFT polymerisation: synthesis,crystallization-driven self-assembly and cellular uptake studies. *Chem. – Eur. J.* **31**, e202500108 (2025). 10.1002/chem.202500108 PMC12015387 39994427

[9] Mahapatra, S., Dey, P. & Ghosh, G. Controlled synthesis of cylindrical micelles via crystallization-driven self-assembly (CDSA) and applications. *Polym. J.* **56**, 949–975 (2024).

[10] Park, S., Kang, S. Y., Yang, S. & Choi, T. L. Independent control of the width and length of semiconducting 2D nanorectangles via accelerated living crystallization-driven self-assembly. *J. Am. Chem. Soc.* **146**, 19369–19376 (2024). 10.1021/jacs.4c05351 38965837

[11] Cai, J. et al. Tailored multifunctional micellar brushes via crystallization-driven growth from a surface. *Science* **366**, 1095–1098 (2019). 10.1126/science.aax9075 31780551

[12] Guo, Y. et al. Real-time label-free imaging of living crystallization-driven self-assembly. *Nat. Commun.* **16**, 2672 (2025). 10.1038/s41467-025-57776-9 PMC11920093 40102380

[13] Teng, F., Xiang, B., Liu, L., Varlas, S. & Tong, Z. Precise control of two-dimensional hexagonal platelets via scalable, one-pot assembly pathways using block copolymers with crystalline side chains. *J. Am. Chem. Soc.* **145**, 28049–28060 (2023). 10.1021/jacs.3c09370 38088129

[14] Tian, J. et al. High-resolution cryo-electron microscopy structure of block copolymer nanofibres with a crystalline core. *Nat. Mater.* **22**, 786–792 (2023). 10.1038/s41563-023-01559-4 37217702

[15] Xie, Y. et al. 2D Hierarchical microbarcodes with expanded storage capacity for optical multiplex and information encryption. *Adv. Mater.* **36**, 2308154 (2024). 10.1002/adma.202308154 38014933

[16] Sahu, A., Mahapatra, S., Dey, P. & Ghosh, G. Harnessing crystallization-driven self-assembly (CDSA) of semicrystalline block copolymers for functional 2D architectures and their applications. *Macromol. Chem. Phys.* **226**, 2400426 (2025).

[17] Lu, Y. et al. Water-soluble micelles with a polyferrocenylsilane core for reductive synthesis of nanomaterials. *J. Am. Chem. Soc.* **148**, 3158–3166 (2026). 10.1021/jacs.5c16479 41513245

[18] MacFarlane, L., Zhao, C., Cai, J., Qiu, H. & Manners, I. Emerging applications for living crystallization-driven self-assembly. *Chem. Sci.* **12**, 4661–4682 (2021). 10.1039/d0sc06878k PMC8179577 34163727

[19] Wu, Y. et al. NIR-actuated morphodynamic 2D nanopatches for interface-programmed immunoactivation and tumor regression. *J. Am. Chem. Soc.* **148**, 2902–2919 (2026). 10.1021/jacs.5c11219 41528161

[20] Wang, Z. et al. Thermoinduced crystallization-driven self-assembly of bioinspired block copolymers in aqueous solution. *Biomacromolecules* **21**, 3411–3419 (2020). 10.1021/acs.biomac.0c00844 32786675

[21] Tao, J. et al. Surface-initiated living crystallization-driven self-assembly: from precision nanofabrication to functional interfaces. *Chem. Soc. Rev.* **55**, 4031–4053 (2026). 10.1039/d6cs00030d 41815063

[22] Yang, K. & Arno, M. C. Synthesis and applications of 1D and 2D nanoparticles prepared through crystallisation-driven self-assembly. *Chem. Sci.* **17**, 13229–13245 (2026). 10.1039/d6sc01312k PMC13237948 42254481

[23] Rozanski, A., Safandowska, M. & Krajenta, A. DSC/SAXS analysis of the thickness of lamellae of semicrystalline polymers-restrictions in the case of materials with swollen amorphous phase. *Polym. Test.* **65**, 189–196 (2018).

[24] Schulz, M., Schäfer, M., Saalwächter, K. & Thurn-Albrecht, T. Competition between crystal growth and intracrystalline chain diffusion determines the lamellar thickness in semicrystalline polymers. *Nat. Commun.* **13**, 119 (2022). 10.1038/s41467-021-27752-0 PMC8748680 35013275

[25] Xia, T. et al. Control over aspect ratio and polymer spatial distribution of 2D platelets via living crystallization-driven self-assembly. *Macromolecules* **57**, 11210–11220 (2024).

[26] Guerin, G., Rupar, P. A. & Winnik, M. A. In-depth analysis of the effect of fragmentation on the crystallization-driven self-assembly growth kinetics of 1D micelles studied by seed trapping. *Polymers* **13**, 3122 (2021). 10.3390/polym13183122 PMC8472273 34578023

[27] Finnegan, J. R., Davis, T. P. & Kempe, K. Heat-induced living crystallization-driven self-assembly: the effect of temperature and polymer composition on the assembly and disassembly of poly(2-oxazoline) nanorods. *Macromolecules* **55**, 3650–3660 (2022).

[28] Xiao, L. et al. Direct preparation of two-dimensional platelets from polymers enabled by accelerated seed formation. *Nat. Synth.* **4**, 808–815 (2025).

[29] Zhu, R., Luo, X., Feng, Y. & Billon, L. CO2-Triggered and temperature-switchable crystallization-driven self-assembly of a semicrystalline block copolymer in aqueous medium. *Polym. Chem.* **10**, 6305–6314 (2019).

[30] Hoffman, J. D. & Miller, R. L. Kinetic of crystallization from the melt and chain folding in polyethylene fractions revisited: theory and experiment. *Polymer* **38**, 3151–3212 (1997).

[31] Hoffman, J. D., Weeks, J. J. & Murphey, W. M. Experimental and theoretical study of kinetics of bulk crystallization in poly(chlorotrifluoroethylene). *J. Res Natl. Bur. Stand A Phys. Chem.* **63A**, 67–98 (1959). 10.6028/jres.063A.005 PMC5287104 31216143

[32] Qiu, H. et al. Uniform patchy and hollow rectangular platelet micelles from crystallizable polymer blends. *Science* **352**, 697–701 (2016). 10.1126/science.aad9521 27151866

[33] Tong, Z. et al. Uniform segmented platelet micelles with compositionally distinct and selectively degradable cores. *Nat. Chem.* **15**, 824–831 (2023). 10.1038/s41557-023-01177-2 PMC10239731 37081206

[34] Liu, L. et al. Synthesis of hollow platelet polymer particles by spontaneous precision fragmentation. *Nat. Synth.* **3**, 903–912 (2024).

[35] Arno, M. C. et al. Precision epitaxy for aqueous 1D and 2D poly(ε-caprolactone) assemblies. *J. Am. Chem. Soc.* **139**, 16980–16985 (2017). 10.1021/jacs.7b10199 PMC5789388 29078700

[36] Tao, R. et al. Program the “light-flashable” 2D fluorescent lifetime microbarcodes for precision information storage. *Adv. Funct. Mater.* **35**, 2500737 (2025).

[37] Xia, T. et al. Tuning the functionality of self-assembled 2D platelets in the third dimension. *J. Am. Chem. Soc.* **145**, 25274–25282 (2023). 10.1021/jacs.3c08770 PMC10682995 37938914

[38] Zhou, T., Qi, H., Han, L., Barbash, D. & Li, C. Y. Towards controlled polymer brushes via a self-assembly-assisted-grafting-to approach. *Nat. Commun.* **7**, 11119 (2016). 10.1038/ncomms11119 PMC4820851 27009369

[39] Sangroniz, L., Alegría, A. & Müller, A. J. Impact of chain length on PCL dielectric β-relaxation: decoupling the roles of chain ends, main chains, entanglements, and crystallinity. *Macromolecules* **58**, 12552–12563 (2025).

[40] Toda, A. Melting kinetics of polymer crystals. *Polym. J.* **57**, 1049–1065 (2025).

[41] Kelly, C. A., Harrison, K. L., Leeke, G. A. & Jenkins, M. J. Detection of melting point depression and crystallization of polycaprolactone (PCL) in scCO2 by infrared spectroscopy. *Polym. J.* **45**, 188–192 (2013).

[42] Zhang, S., Wang, Z., Guo, B. & Xu, J. Secondary nucleation in polymer crystallization: a kinetic view. *Polym. Cryst.* **4**, e10173 (2021).

[43] Fernández-Tena, A. et al. Effect of molecular weight on the crystallization and melt memory of poly(ε-caprolactone) (PCL). *Macromolecules* **56**, 4602–4620 (2023).

