# Supplementary material for: Thermally programmed disassembly and reconstruction of 2D polymer platelets via melting-governed crystallization-driven self-assembly
Source: Nat Commun. 2026 Oct 3;17:11304. doi: 10.1038/s41467-026-77973-4 (PMC13724731; doi:10.1038/s41467-026-77973-4)
Supplement: Supplementary file 1 [file 41467_2026_77973_MOESM1_ESM.pdf]

Supplementary Information for

Thermally programmed disassembly and reconstruction of 2D polymer platelets *via* melting-governed crystallization-driven self-assembly

Emre Turan[1], Maria C. Arno*[1,2]

[1] School of Chemistry, University of Birmingham, Edgbaston, Birmingham, B15 2TT, United Kingdom

[2] Institute of Cancer and Genomic Sciences, University of Birmingham, Edgbaston, Birmingham, B15 2TT, United Kingdom

Materials

All reagents and solvents were used as received unless otherwise specified. Sodium ethanethiolate (90%), carbon disulfide (99%), solid iodine (99%), 4,4'-azobis(4-cyanovaleric acid) (ACVA, 98%), and borane tetrahydrofuran complex solution (1.0 M in THF) were purchased from Sigma-Aldrich. The dual-head chain transfer agent (CTA), 2-cyano-5-hydroxypentan-2-yl ethyl carbonotrithioate (CHPET), was synthesized according to previously reported procedures.[1] Before use, the CTA and diphenyl phosphate (DPP, 99%, Sigma-Aldrich) were dried over phosphorus pentoxide in a desiccator under static vacuum for one week. ε-Caprolactone (99%, ACROS Organics) was purified by vacuum distillation twice over calcium hydride, then transferred into a glovebox for storage and subsequent use. 2,2'-Azobis(2-methylpropionitrile) (AIBN, 98%, Sigma-Aldrich) was recrystallized twice from methanol and stored in the dark at 4 °C. 1,4-Dioxane (anhydrous, 99.8%, Fisher Scientific) and *N,N*-dimethylacrylamide (DMA, 99%, containing 500 ppm monomethyl ether hydroquinone inhibitor, Sigma-Aldrich) were purified by passing through a basic alumina column immediately prior to use to remove stabilizers. Anhydrous solvents were obtained directly from a drying and degassing inert solvent purification system. Carboxylic acid-functionalized BODIPY derivatives (FL and 630/650) were purchased from Lumiprobe and used as supplied without additional purification.

Characterization and Instrumentation

Nuclear Magnetic Resonance (NMR)

^1H NMR spectra were acquired using a Bruker 400 MHz (DPX-400) spectrometer. Chemical shifts (δ) are reported in parts per million (ppm) relative to tetramethylsilane (TMS) as the internal reference (δ = 0 ppm). Samples were dissolved in deuterated chloroform ($CDCl_3$), and spectra were referenced to the residual non-deuterated solvent signal ($CDCl_3$, δ = 7.26 ppm in ^1H NMR spectroscopy). Signal multiplicities are denoted as s (singlet), d (doublet), t (triplet), q (quartet), and m (multiplet).

Size Exclusion Chromatography (SEC)

SEC measurements were carried out on an Agilent 1260 Infinity II system equipped with refractive index (RI) and ultraviolet (UV) detectors. The UV-Vis detector wavelength was set to 309 nm for the unfunctionalized polymer and adjusted to 500 and 600 nm when required. The system incorporated a PLGel 3 µm guard column (50 × 7.5 mm) followed by two PLGel 5 µm mixed-D columns (300 × 7.5 mm). Chloroform ($CHCl_3$) containing 0.5% triethylamine (NEt_3) was used as the mobile phase. Number-average molecular weight (M_n), weight-average molecular weight (M_w), and dispersity ($Đ = M_w/M_n$) were determined by calibration against poly(methyl methacrylate) (PMMA) standards. Data processing was performed using Agilent SEC software.

Nano Differential Scanning Calorimetry (nanoDSC)

Thermal measurements of solution-grown platelets were conducted using a TA Instruments nanoDSC. Platelet dispersions in ethanol were analyzed directly without prior drying. Heating scans were performed at 1 °C min^{-1} unless otherwise specified.

Wide-angle X-ray Scattering (WAXS)

WAXS measurements were performed using a Bruker D8 diffractometer equipped with Cu Kα radiation (λ = 1.5418 Å) and a linear position-sensitive detector. Diffraction patterns were collected over a 2θ range of 5-45° with a step size of 0.02°.

Differential Scanning Calorimetry (DSC)

Thermal properties of the polymers were evaluated using a STARe system DSC3 with an auto-sampler (Mettler Toledo, Switzerland). Disc-shaped specimens (5 mm diameter, 0.4-0.6 mm thickness) weighing between 4-10 mg were prepared and placed in 40 µL aluminum pans. Measurements were conducted over a temperature range of -100 to 100 °C at a heating and cooling rate of 10 K min^{-1} for three consecutive cycles.

Transmission Electron Microscopy (TEM)

Dry-state TEM characterization was performed using a JEOL 1400 Bio (1720/GB06) microscope operating at an acceleration voltage of 80 kV. Sample solutions were deposited onto formvar-coated copper grids and allowed to dry. Imaging was generally carried out without staining unless otherwise indicated. For particle size analysis, a minimum of 100 nanostructures were measured in each sample.

Selected Area Electron Diffraction (SAED)

SAED characterization was performed using a JEOL 1400 Bio (1720/GB06) microscope operating at an acceleration voltage of 80 kV with a camera length of 100 cm. Sample solutions were deposited onto formvar-coated copper grids and allowed to dry.

Atomic Force Microscopy (AFM)

AFM samples were prepared by depositing small aliquots (10-20 μL) of platelet dispersions onto freshly cleaned glass coverslips, followed by spin-coating to ensure uniform film formation. Imaging was performed using a JPK NanoWizard 4 system in quantitative imaging (QI) mode. PPP-NCHAuD probes (NanoSensors) with nominal resonance frequencies between 200 and 500 kHz and spring constants in the range 10-130 N m^{-1} were employed. Height analysis and lamellar thickness measurements were obtained using JPK Data Processing software, with at least 100 platelets analyzed for each condition.

Confocal Fluorescence Microscopy

Fluorescence imaging was conducted on an Olympus FV3000 confocal microscope equipped with a 60× oil-immersion objective. Excitation wavelengths of 514 nm and 640 nm were used to address the corresponding fluorescent labels selectively. Emission was collected over appropriate spectral windows. Image processing and analysis were carried out using Olympus CellSens software and ImageJ.

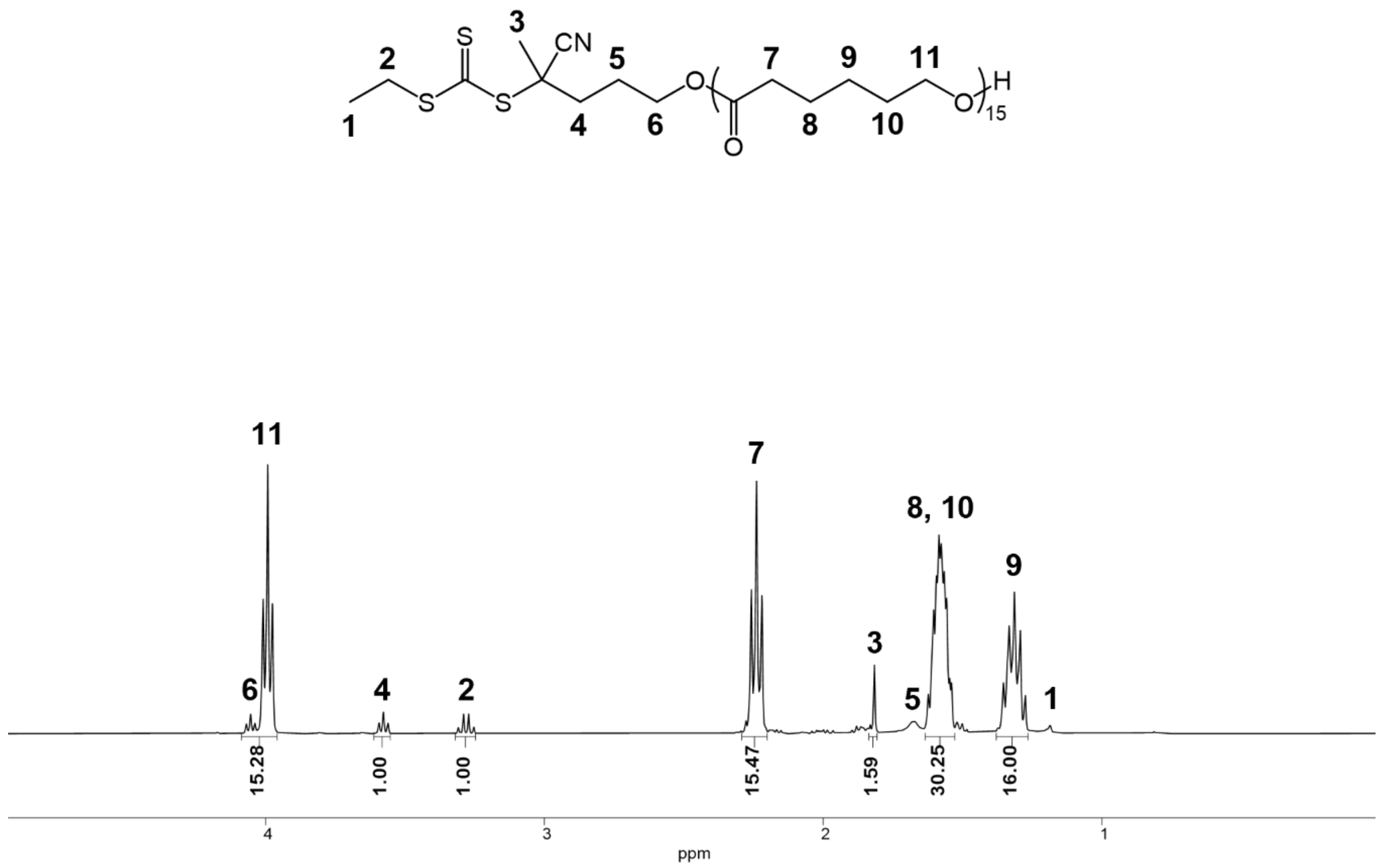

Supplementary Figure 1. ^{1}H NMR spectrum (400 MHz, $CDCl_3$) of PCL_{15}.

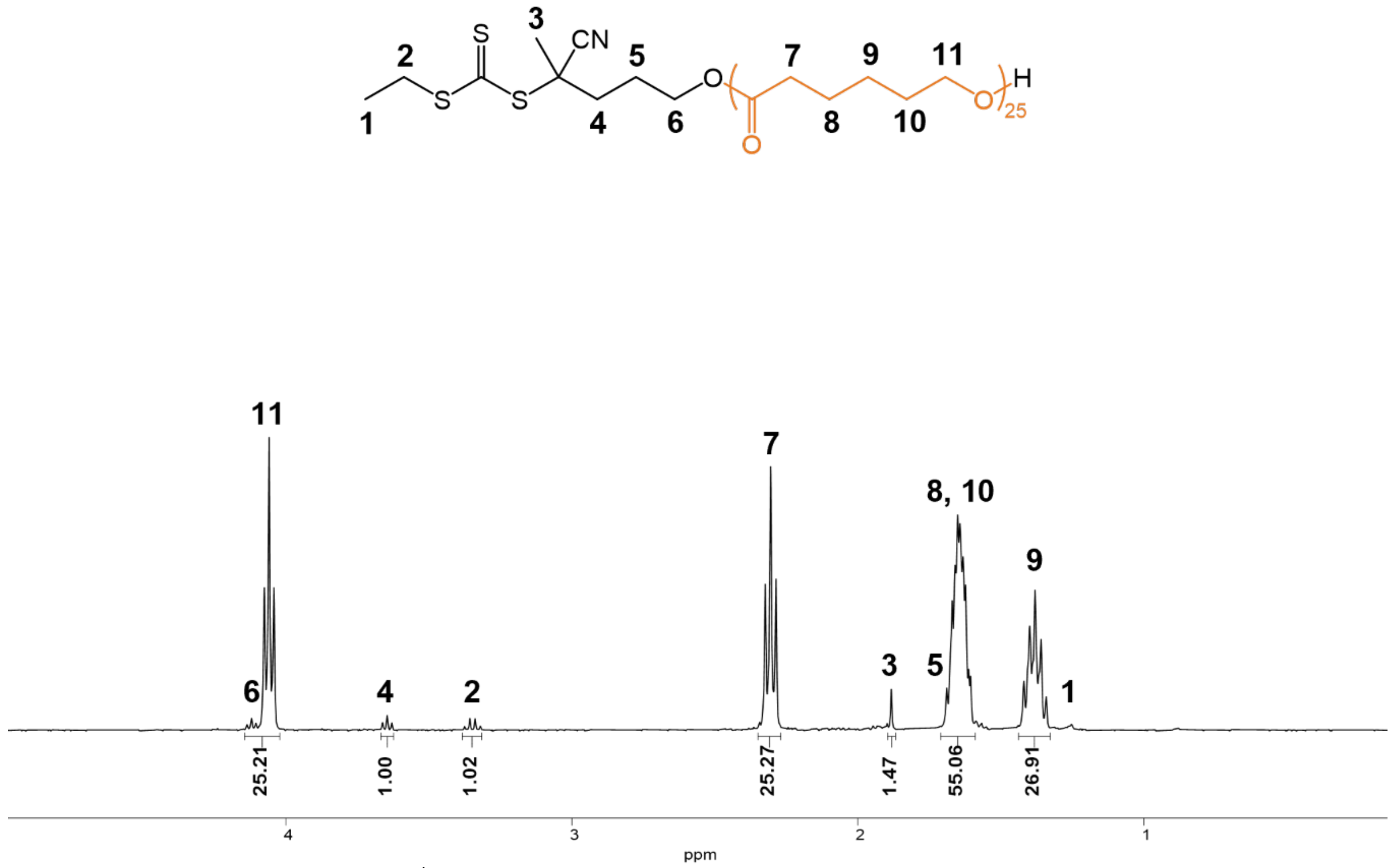

Supplementary Figure 2. ^{1}H NMR spectrum (400 MHz, $CDCl_3$) of PCL_{25}.

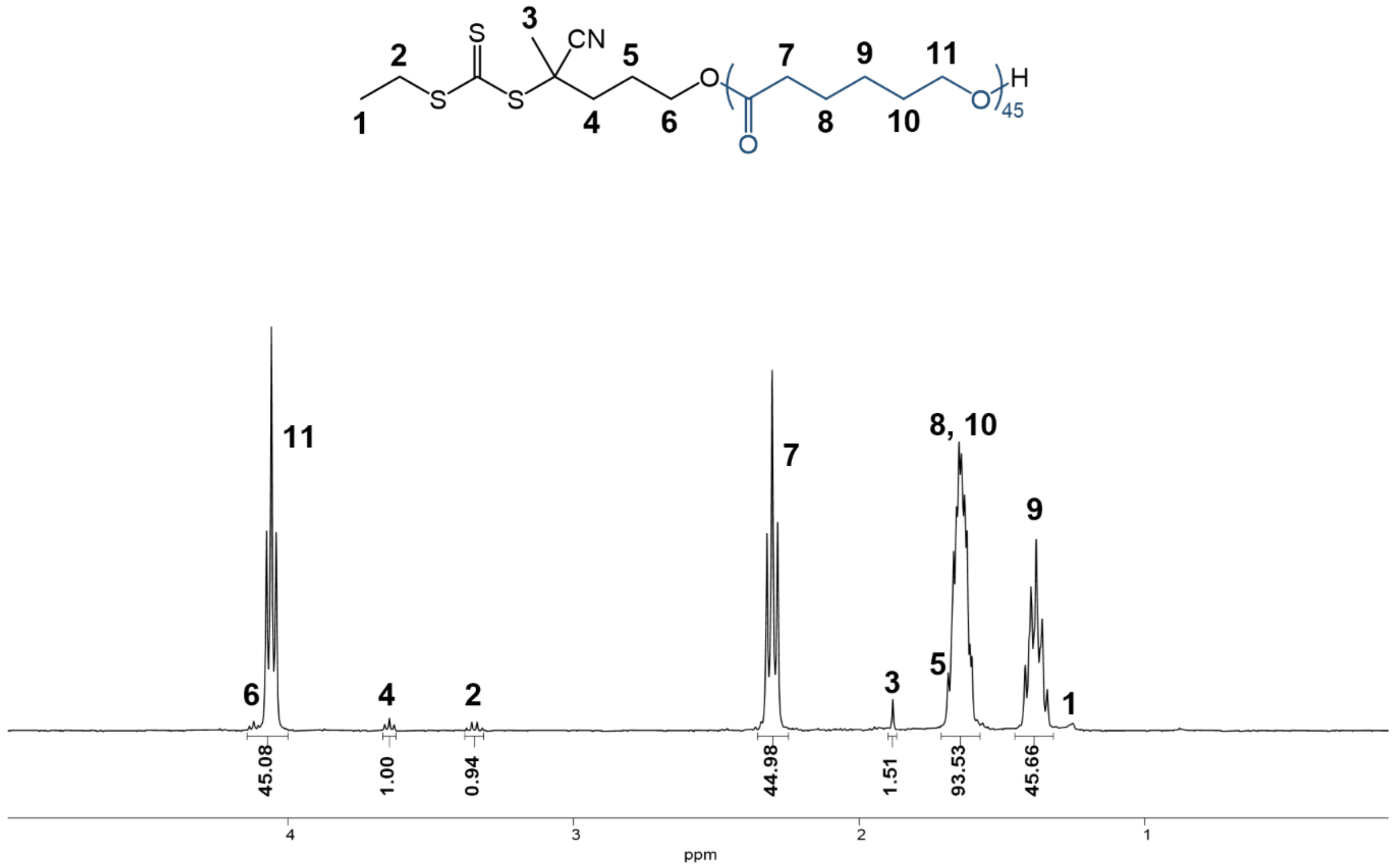

Supplementary Figure 3. ^{1}H NMR spectrum (400 MHz, $CDCl_3$) of PCL_{45}.

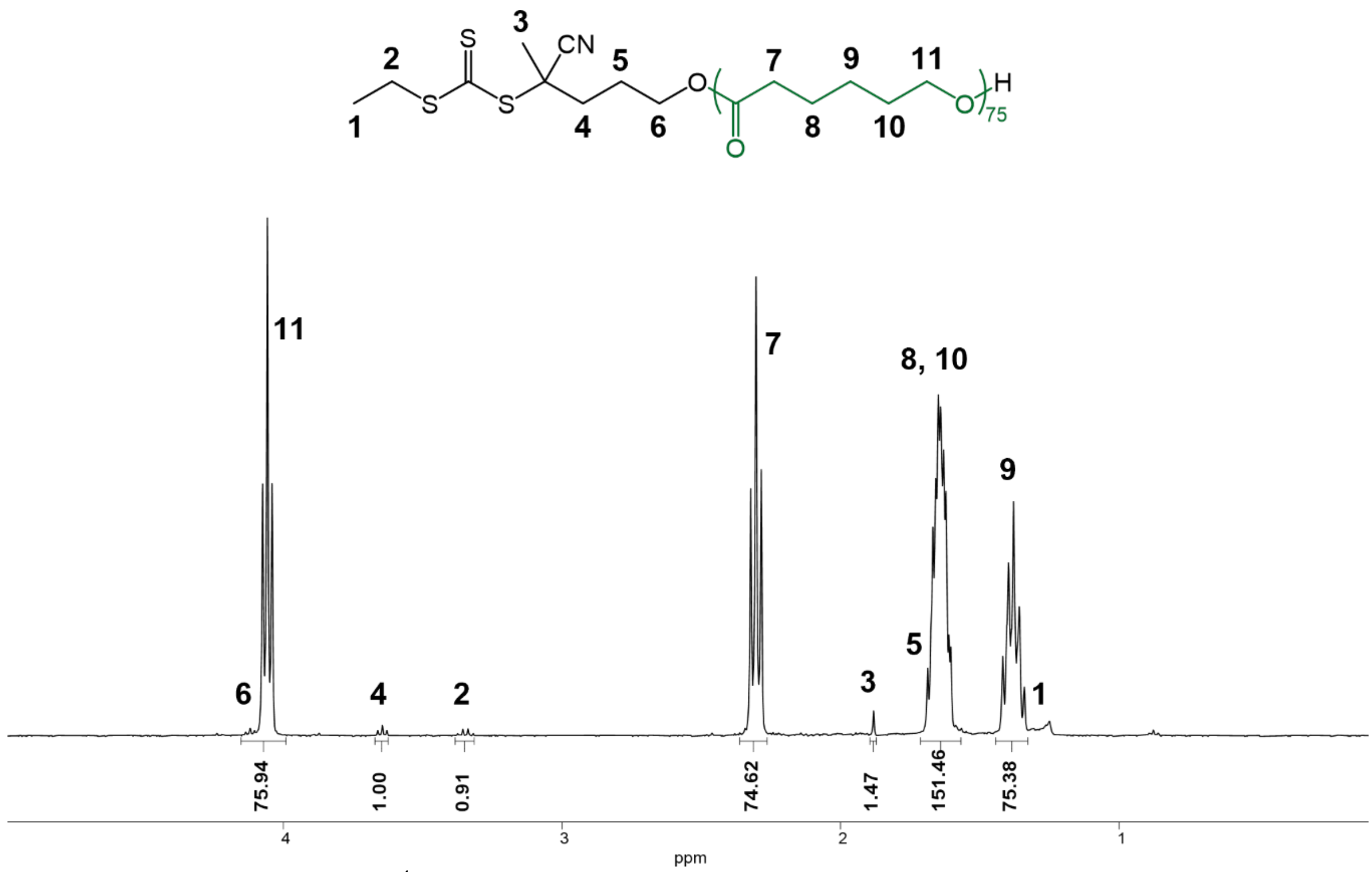

Supplementary Figure 4. ^{1}H NMR spectrum (400 MHz, $CDCl_3$) of PCL_{75}.

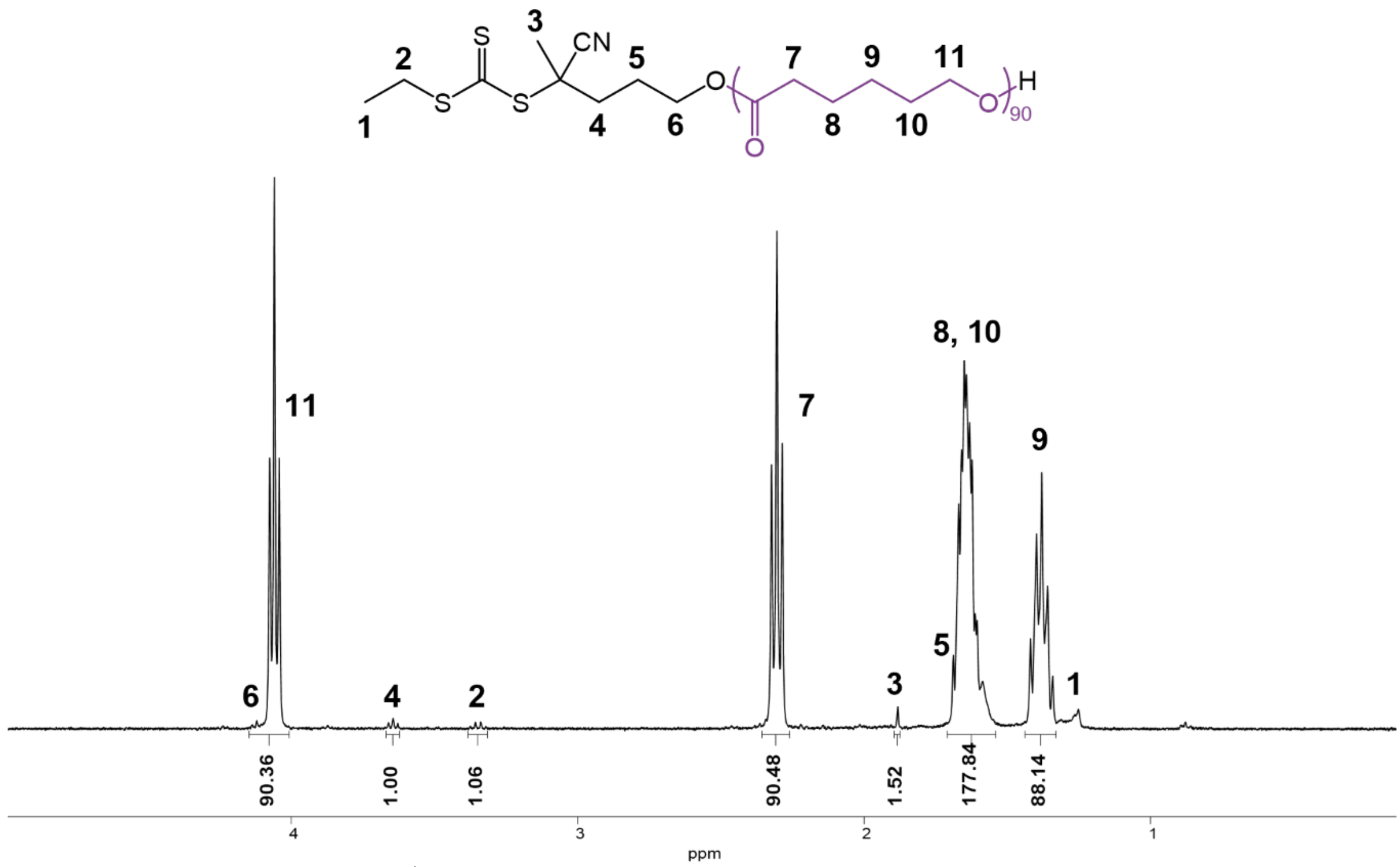

Supplementary Figure 5. ^1H NMR spectrum (400 MHz, $CDCl_3$) of PCL_{90}.

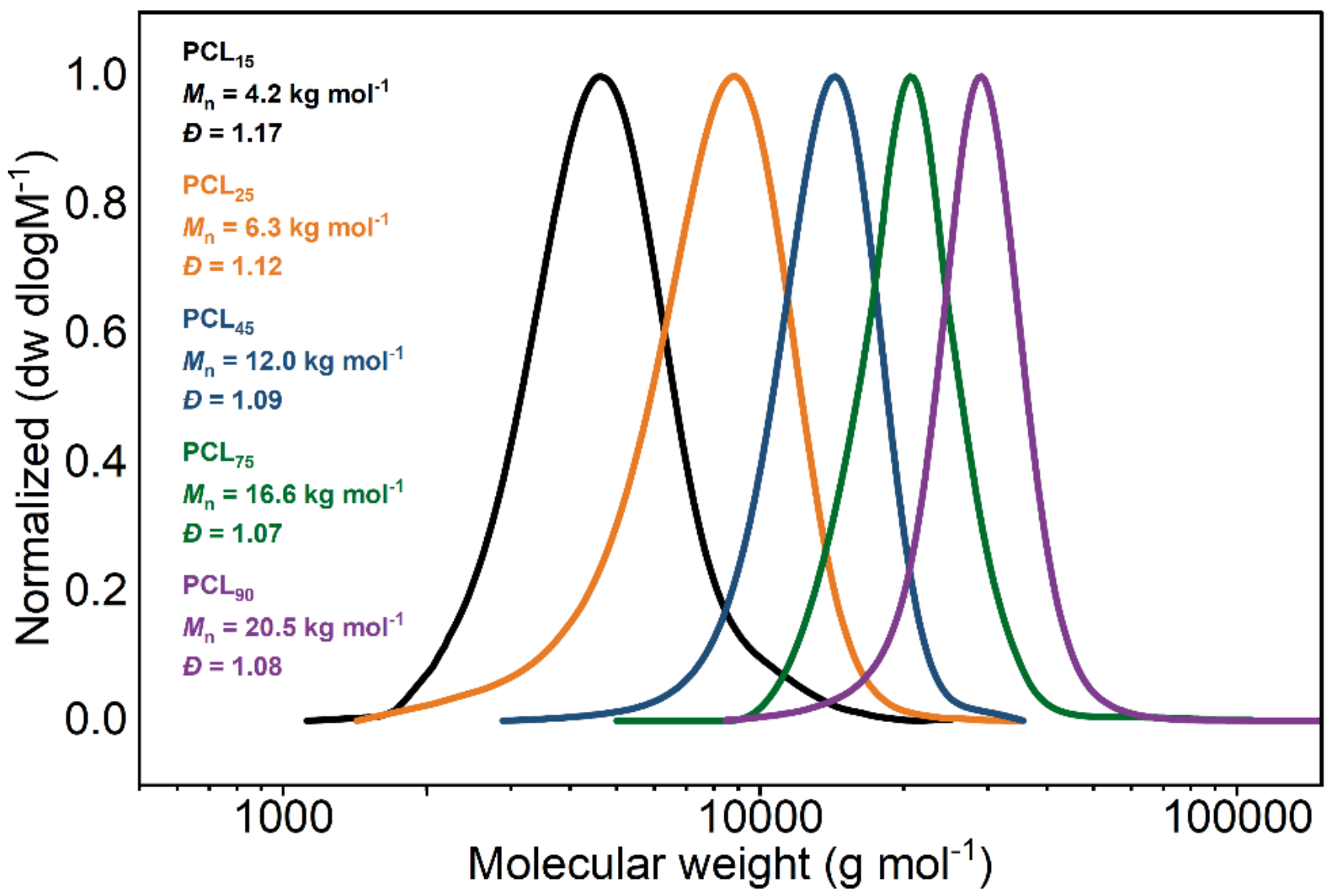

Supplementary Figure 6. Size exclusion chromatograms of PCL_{15}, PCL_{25}, PCL_{45}, PCL_{75}, and PCL_{90} (RI, $CHCl_3$ with 0.5% NEt_3 as an eluent against PMMA standard).

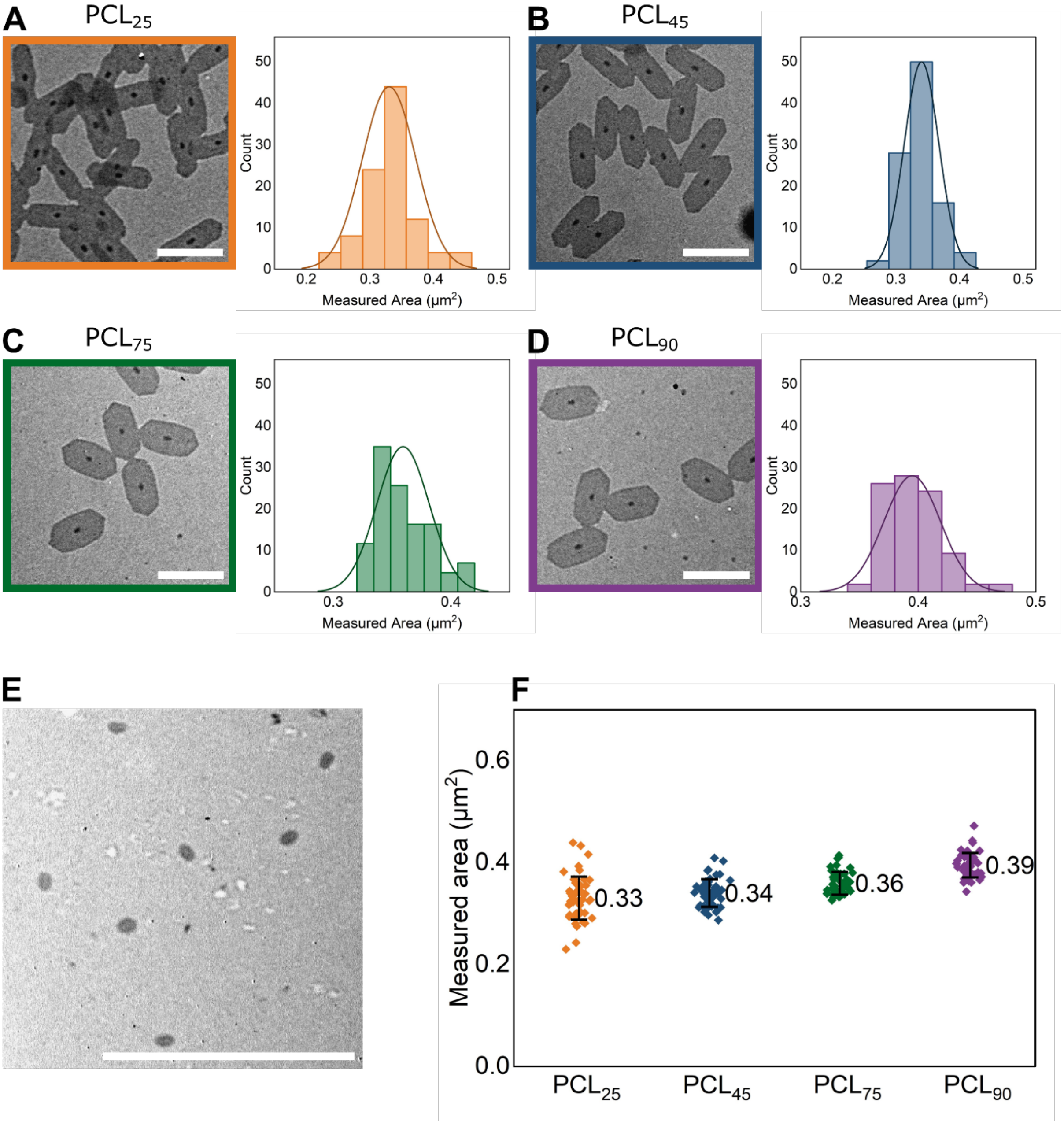

Supplementary Figure 7. Statistical analysis of the surface area of pure PCL platelets formed from different chain lengths at 20 °C. TEM micrographs of platelets prepared from **(a)** PCL_{25}, **(b)** PCL_{45}, **(c)** PCL_{75}, and **(d)** PCL_{90} were analyzed to determine the surface area using ImageJ, and the results are presented as distribution bar charts (at least 100 platelets were measured per polymer chain length). **(e)** PCL_{50}-*b*-$PDMA_{200}$ seeds were used to make said platelets. All scale bars are equivalent to 1 μm. **(f)** Scatter plot comparing the measured platelet areas against the different chain lengths. Data are presented as mean values ± SD (n = 100).

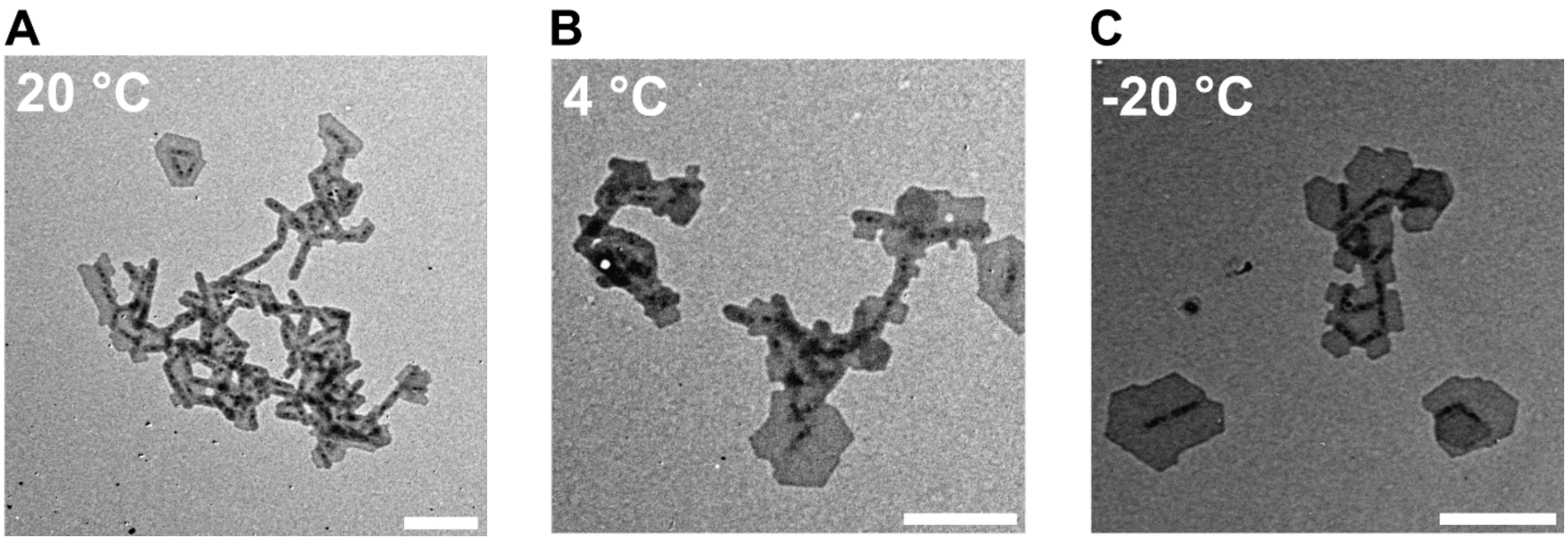

Supplementary Figure 8. TEM micrographs showing the formation of PCL_{15} platelets at **(a)** 20 °C, **(b)** 4 °C, and **(c)** -20 °C. All scale bars are equivalent to 1 μm. Crystallization of PCL_{15} did not provide sufficient enthalpic driving force to form uniform 2D platelets, instead yielding ribbon-like, non-uniform structures.

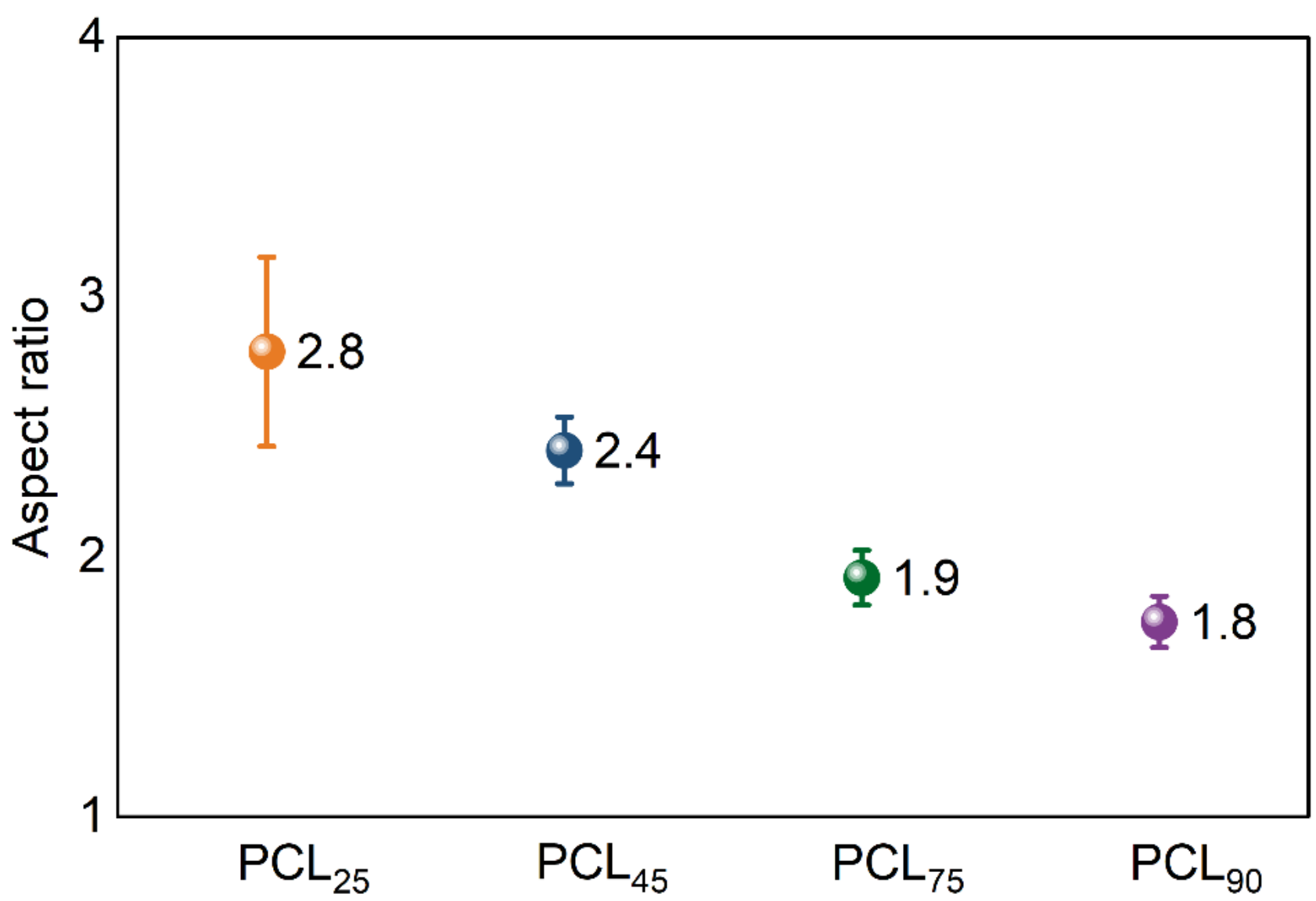

Supplementary Figure 9. Aspect ratio of PCL platelets formed from different chain lengths at 20 °C. The length and width of at least 100 platelets composed of PCL_{25}, PCL_{45}, PCL_{75}, and PCL_{90} were measured from TEM micrographs using ImageJ to calculate their corresponding aspect ratios. Data are presented as mean values ± SD (n = 100). A general trend was observed in which the aspect ratio increased with decreasing PCL chain length. This is hypothesized to result from slower crystallization rates at lower chain lengths, promoting preferential growth along the (110) crystalline plane (length) and leading to the formation of more slender, higher-aspect ratio platelets.

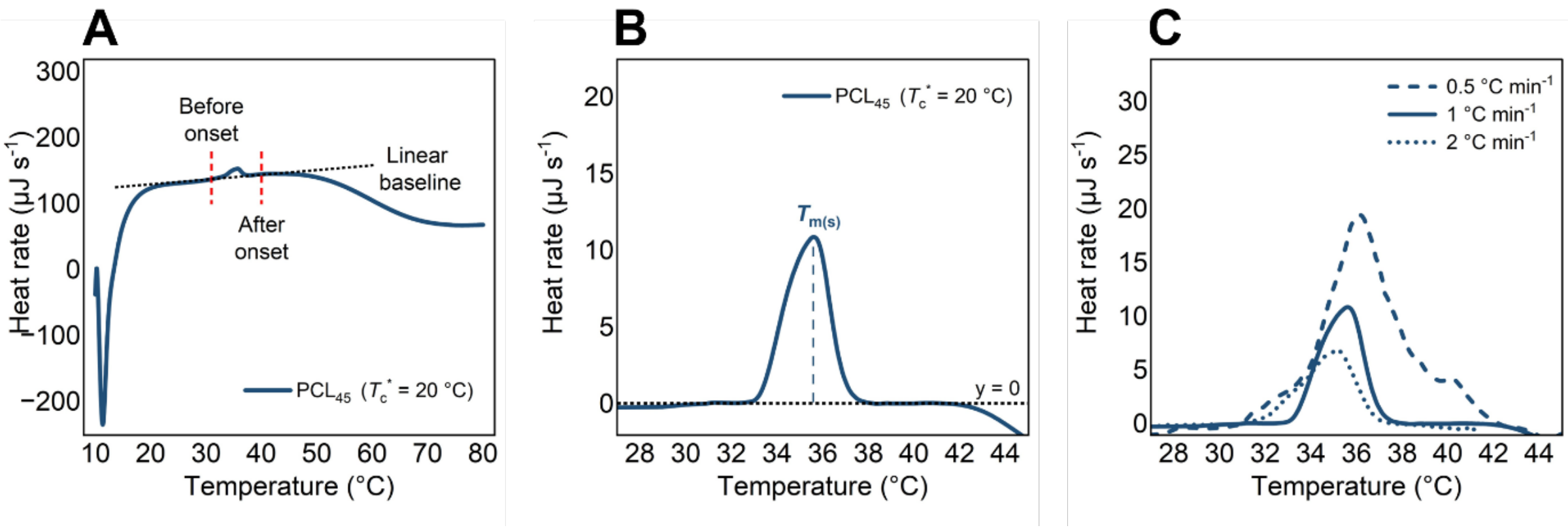

Supplementary Figure 10. (a) Raw nanoDSC heat flow trace of PCL_{45} platelets crystallized at T_c^* = 20 °C. **(b)** Corresponding melting peak after linear baseline subtraction performed using the TA Instruments nanoDSC analysis software, with the extracted peak used to determine $T_{m(s)}$. **(c)** NanoDSC heat flow traces of PCL_{45} platelets crystallized at T_c^* = 20 °C at three different scan rates: 0.5, 1, and 2 °C min^{-1}. 1 °C min^{-1} was found to be the most suitable scan rate for crystalline PCL platelets dispersed in ethanol.

Supplementary Table 1. Degree of crystallinity determination of PCL samples from DSC and nanoDSC melting curves.

**Polymer**	**Mass enthalpy $\Delta H_{m(DSC)}$ [a] (J g^{-1})**	**Crystallinity $X_{c(DSC)}$ [b] (%)**	**Mass enthalpy $\Delta H_{m(s)}$ [c] (J g^{-1})**	**Crystallinity $X_{c(s)}$ [d] (%)**
PCL_{25}	85	63	65	48
PCL_{45}	84	62	98	73
PCL_{75}	79	58	122	91
PCL_{90}	69	51	127	94

[a] Obtained by integrating DSC melting peaks.

[b] Determined by DSC.

[c] Calculated from nanoDSC melting peaks.

[d] Determined by nanoDSC.

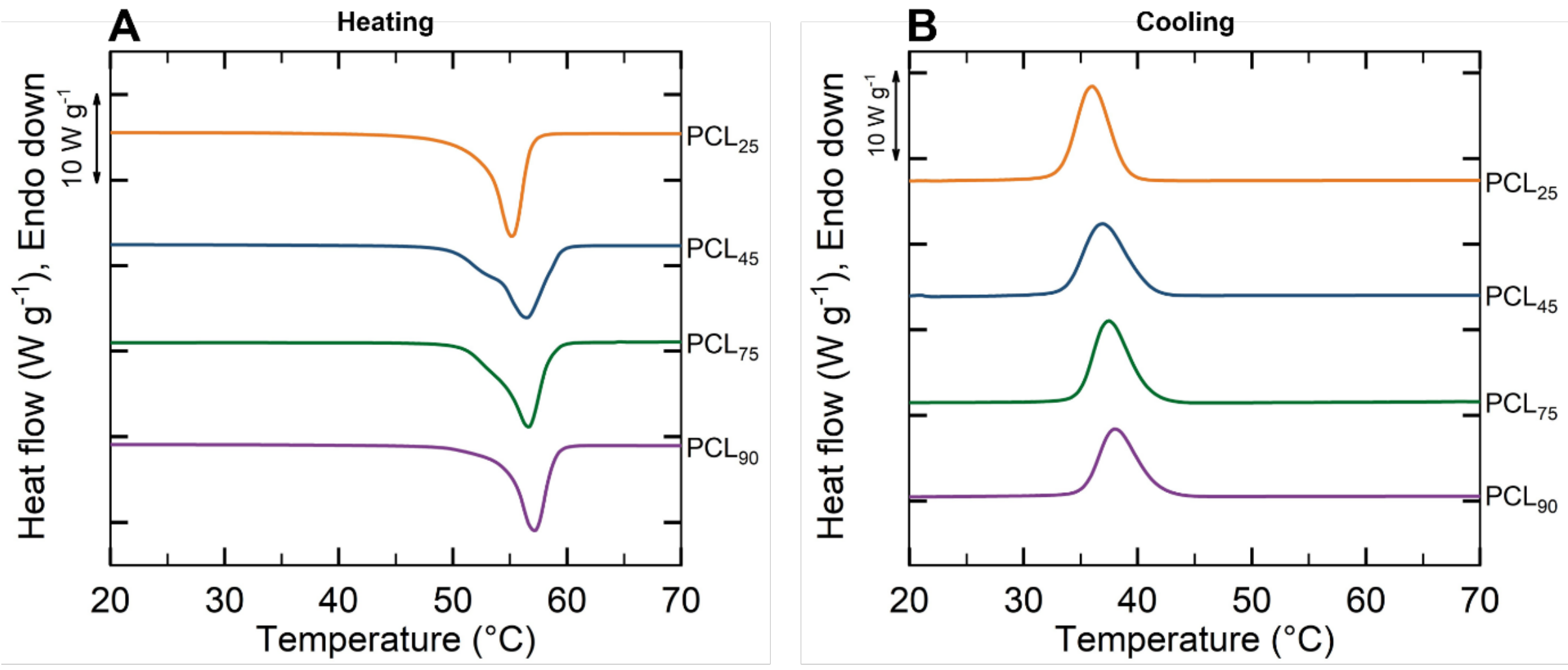

Supplementary Figure 11. Differential scanning calorimetry (DSC) of bulk PCL samples. **(a)** Heating and **(b)** cooling DSC curves of PCL_{25}, PCL_{45}, PCL_{75}, and PCL_{90}.

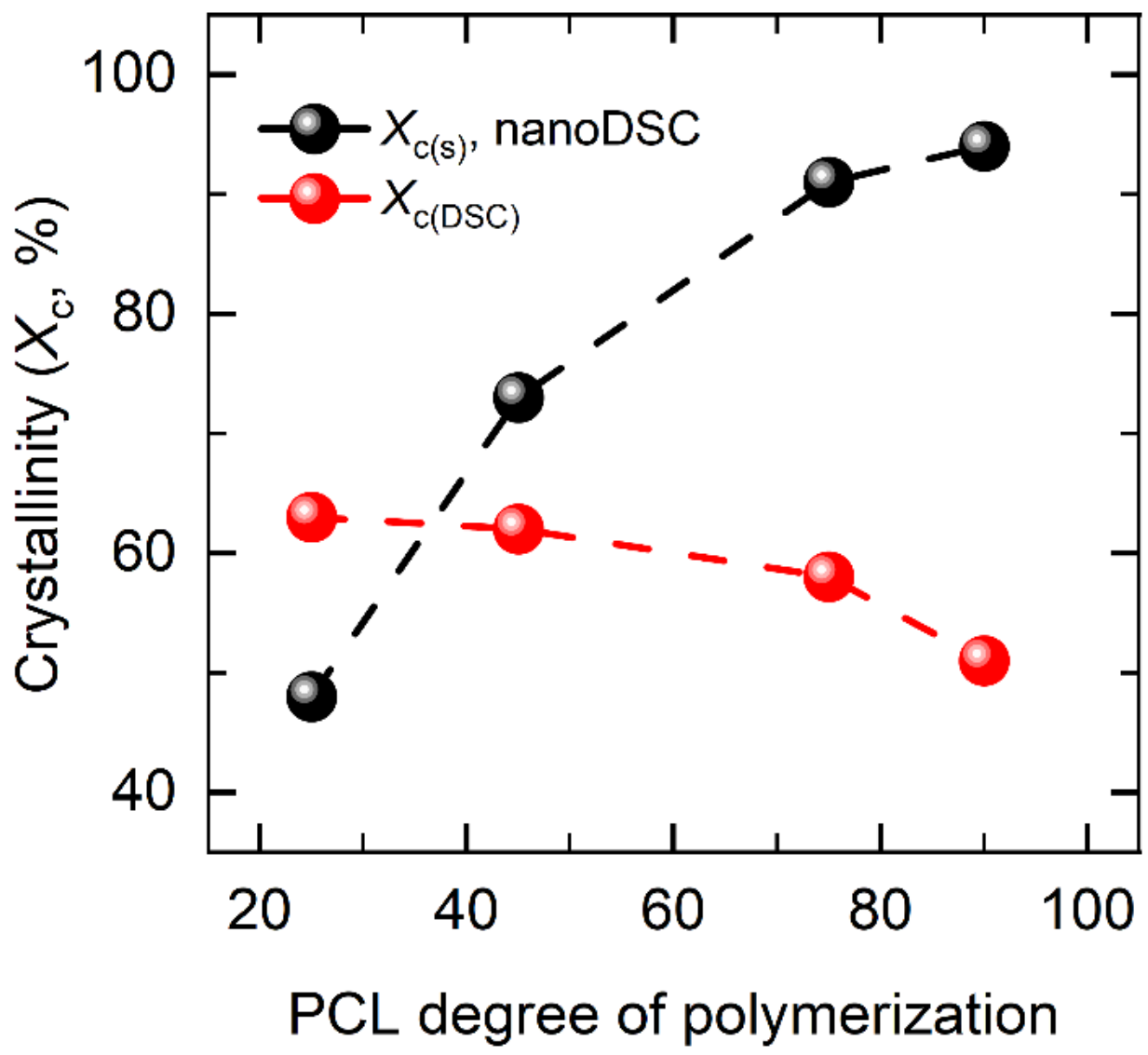

Supplementary Figure 12. Comparison of degree of crystallinity calculated from nanoDSC *vs* DSC as a function of PCL degree of polymerization.

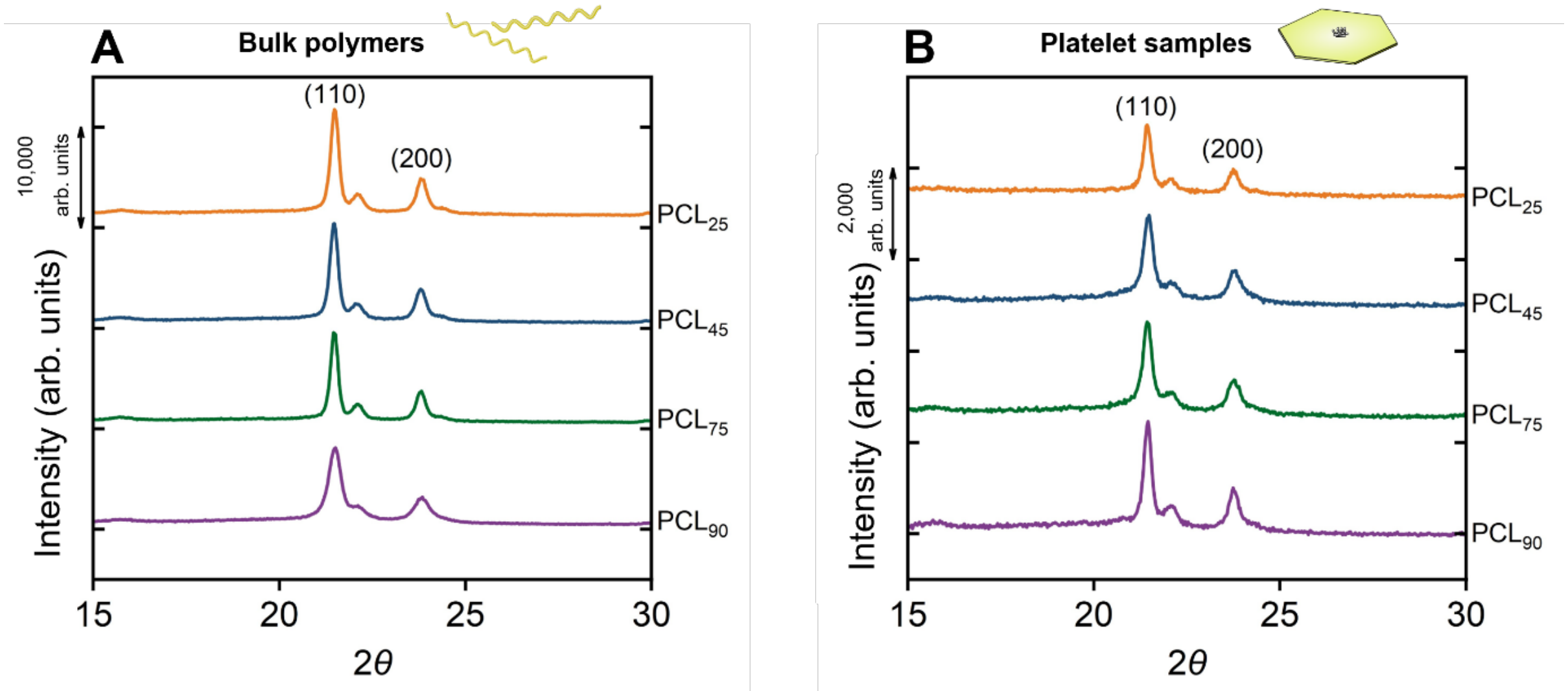

Supplementary Figure 13. Wide-angle X-ray scattering (WAXS) of bulk PCL and self-assembled PCL platelets. **(a)** WAXS patterns of bulk PCL_{25}, PCL_{45}, PCL_{75}, and PCL_{90} samples. **(b)** WAXS patterns of self-assembled PCL_{25}, PCL_{45}, PCL_{75}, and PCL_{90} platelet samples.

Supplementary Table 2. Degree of crystallinity determination from WAXS for bulk polymers and self-assembled platelets.

**Polymer**	**Crystallinity** $X_{c(WAXS, bulk)}$ [a] **(%)**	**Crystallinity** $X_{c(WAXS, platelet)}$ [a] **(%)**
PCL_{25}	64	51
PCL_{45}	59	69
PCL_{75}	56	75
PCL_{90}	52	87

[a] Determined by WAXS.

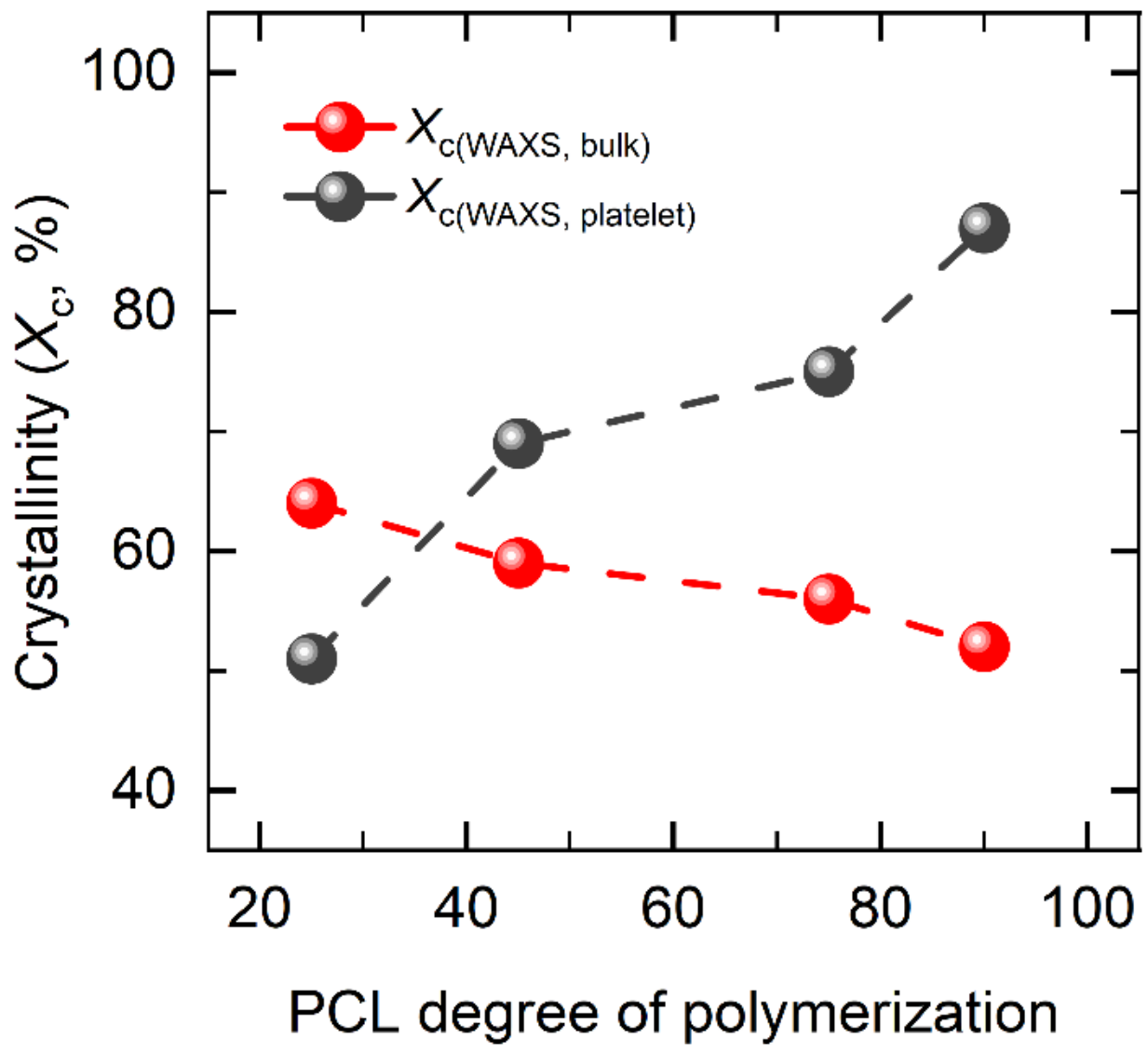

Supplementary Figure 14. Comparison of degree of crystallinity calculated from nanoDSC *vs* DSC as a function of PCL degree of polymerization.

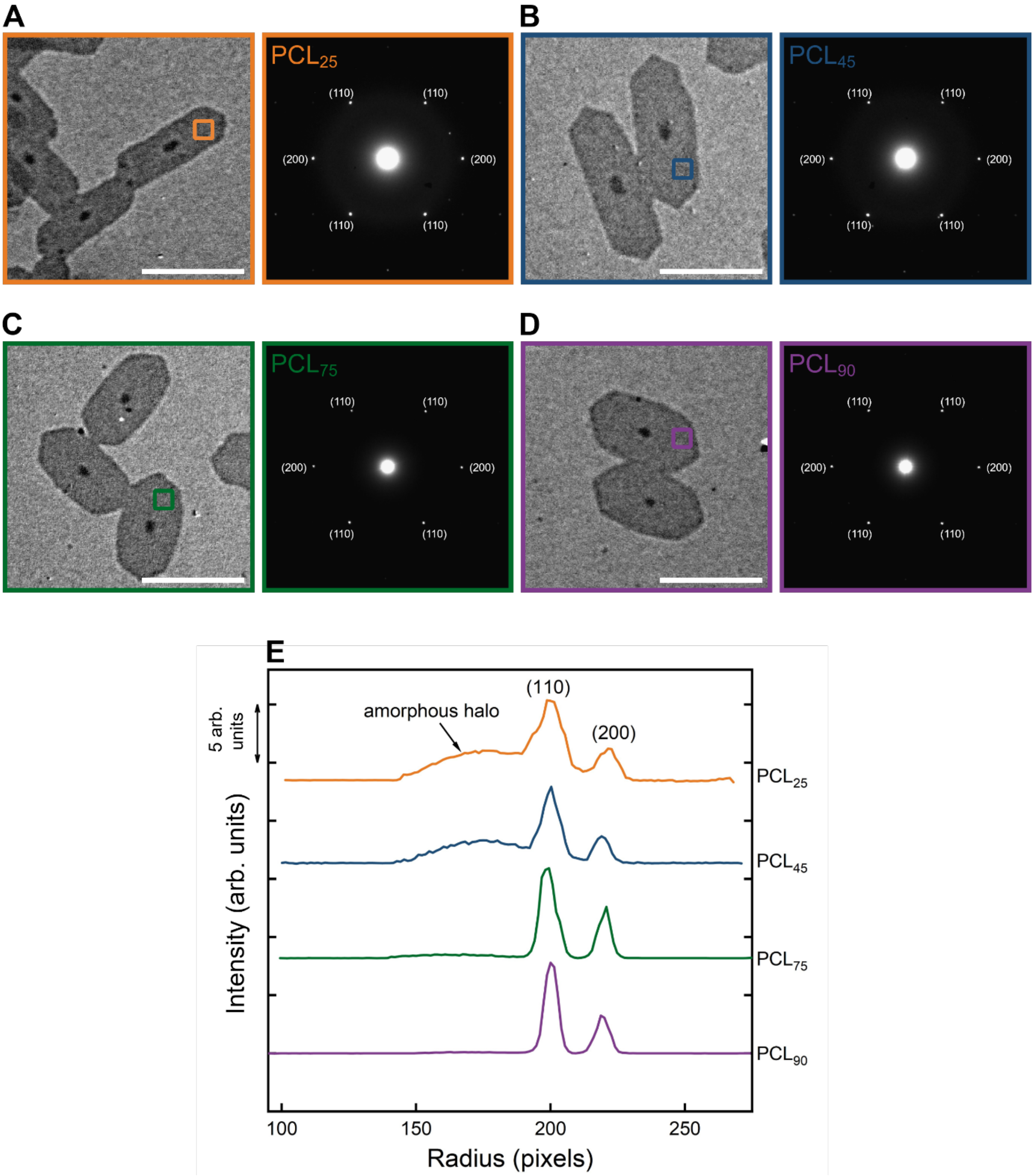

Supplementary Figure 15. Selected area electron diffraction (SAED) analysis and crystallinity determination of self-assembled PCL platelets. SAED patterns of **(a)** PCL_{25}, **(b)** PCL_{45}, **(c)** PCL_{75}, and **(d)** PCL_{90} platelets, showing the (110) and (200) crystalline reflections. All scale bars are equivalent to 1 µm. **(e)** Radial intensity profiles of the SAED patterns, showing the amorphous halo and the (110) and (200) crystalline reflections.

Supplementary Table 3. *D*-spacing and degree of crystallinity determination from SAED radial intensity profiles.

Polymer	Crystal Plane	*d*-spacing (nm) [a]	Crystallinity $X_{c(SAED)}$ [a] (%)
PCL_{25}	(110)	0.408	46
	(200)	0.366	
PCL_{45}	(110)	0.408	57
	(200)	0.371	
PCL_{75}	(110)	0.411	88
	(200)	0.373	
PCL_{90}	(110)	0.410	95
	(200)	0.371	

[a] Determined by SAED.

According to the Gibbs-Thomson relation, the melting temperature of a polymer crystal decreases as its lamellar thickness decreases, reflecting the higher surface-to-volume ratio and resulting increase in surface free energy (σ_e) associated with a thinner lamella:

$$T_m(l) = T_m^0\left(1 - \frac{2\sigma_e}{l\Delta H_f}\right)$$

Supplementary Equation (1). Gibbs-Thomson equation.

where $T_m(l)$ is the melting temperature of a crystal with l lamellar thickness, T_m^0 is the equilibrium melting temperature (melting temperature of an infinitely thick, defect-free crystal), σ_e is the surface free energy of the crystal fold surface, and ΔH_f is the enthalpy of fusion per unit volume.

Because lamellar thickness is largely established during crystallization, a second equation, the Hoffmann-Weeks interpretation for lamellar thickness, provides the thermodynamic link between crystallization temperature, resulting lamellar thickness, and the corresponding melting temperature:

$$l = \frac{2\sigma_e T_m^0}{\Delta H_f(T_m^0 - T_c^*)}$$

Supplementary Equation (2). Hoffmann-Weeks equation.

where T_c^* is the temperature of the solution during crystallization (crystallization temperature).

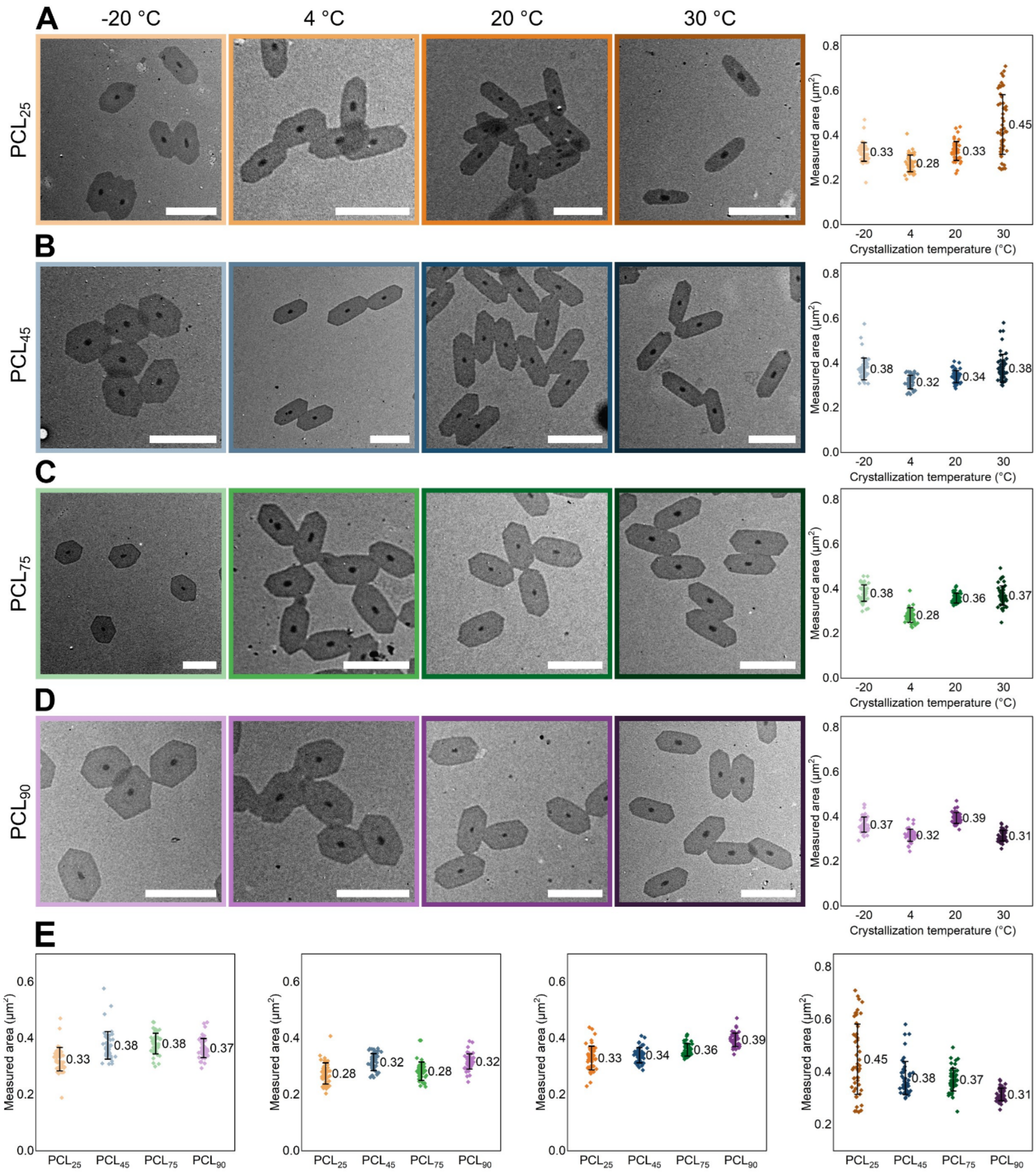

Supplementary Figure 16. Statistical analysis of the surface area of pure PCL platelets formed from different chain lengths at various crystallization temperatures. TEM micrographs of platelets composed of **(a)** PCL_{25}, **(b)** PCL_{45}, **(c)** PCL_{75}, and **(d)** PCL_{90}, crystallized at -20 °C, 4 °C, 20 °C, and 30 °C, were analyzed to determine the surface area of at least 100 platelets using ImageJ, and the results are presented as distribution bar charts. All scale bars are equivalent to 1 μm. **(e)** The measured platelet areas for each chain length at -20 °C, 4 °C, 20 °C, and 30 °C (left to right) are additionally compared in scatter plots. Data are presented as mean values ± SD (n = 100). Platelet uniformity was improved at moderate crystallization temperatures (4 and 20 °C) compared to more extreme conditions (-20 and 30 °C), with this effect becoming more pronounced as the PCL chain length decreased. In particular, PCL_{25} platelets formed at 30 °C exhibited a broad distribution of surface areas. This may be attributed to reduced supercooling, which lowers the crystallization rate while increasing chain mobility.

As a result, polymer chains remain in solution for longer, allowing seeds more time to aggregate prior to crystallization, ultimately leading to less uniform platelet formation.

Supplementary Table 4. Thermodynamic analysis of platelets made from differing PCL chain lengths.

**HPs**	**Crystallization temperature T_c* (°C)**	**Lamellar thickness l [a] (nm)**	**Melting temperature $T_{m(s), nanoDSC}$ [b] (°C)**	**Apparent equilibrium melting temperature $T_m^0{}_{(s)}$ [c] (°C)**	**Apparent surface free energy σ_e [d] (mJ m^{-2})**
PCL_{25}	-20	8.01 ± 0.46	34.1	38.7	28.3
	4	8.86 ± 0.33	35.3		
	20	10.45 ± 0.35	37.1		
	30	10.35 ± 0.63	37.6		
PCL_{45}	-20	7.43 ± 0.32	33.5	38.5	29.9
	4	8.29 ± 0.37	34.4		
	20	9.12 ± 0.27	35.7		
	30	10.12 ± 0.42	37.9		
PCL_{75}	-20	6.67 ± 0.52	35.5	40.2	18.3
	4	7.82 ± 0.27	36.5		
	20	8.58 ± 0.20	37.7		
	30	9.59 ± 0.27	38.9		
PCL_{90}	-20	7.03 ± 0.40	36.4	41.3	18.0
	4	7.66 ± 0.37	37.7		
	20	8.99±0.36	38.5		
	30	9.66 ± 0.53	39.8		

[a] Obtained from AFM height profiles.

[b] Obtained from nanoDSC.

[c] Calculated from Hoffmann-Weeks analysis (Supplementary Figure 17).

[d] Calculated from the Gibbs-Thomson analysis (Supplementary Figure 19).

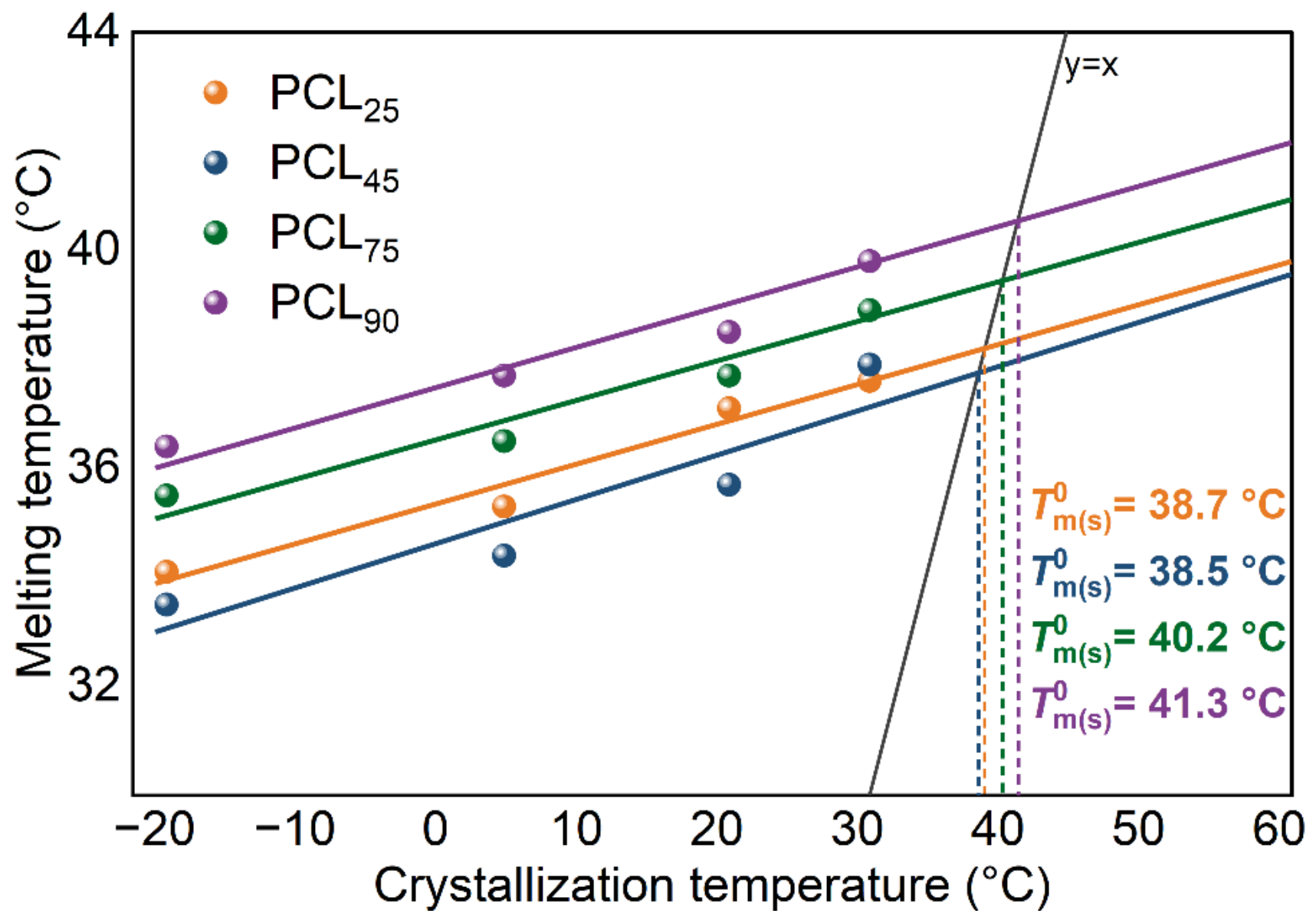

Supplementary Figure 17. Hoffman-Weeks analysis of melting temperatures for platelets formed from different PCL chain lengths. The melting temperatures of platelets composed of PCL_{25}, PCL_{45}, PCL_{75}, and PCL_{90}, measured by nanoDSC, are plotted against their respective crystallization temperatures. This provides a quantitative approach to extrapolate the equilibrium melting temperatures for each PCL chain length. The equilibrium melting point is determined at the intersection of the line of best fit with the y = x line. It is important to note that the melting temperatures observed for the platelets differ from their bulk melting points; consequently, the extrapolated equilibrium melting temperatures also differ from bulk values. This discrepancy arises because the platelets are finite, defect-containing crystals formed in ethanol. Therefore, these values represent the apparent equilibrium melting temperatures of PCL specifically within ethanol-formed platelets.

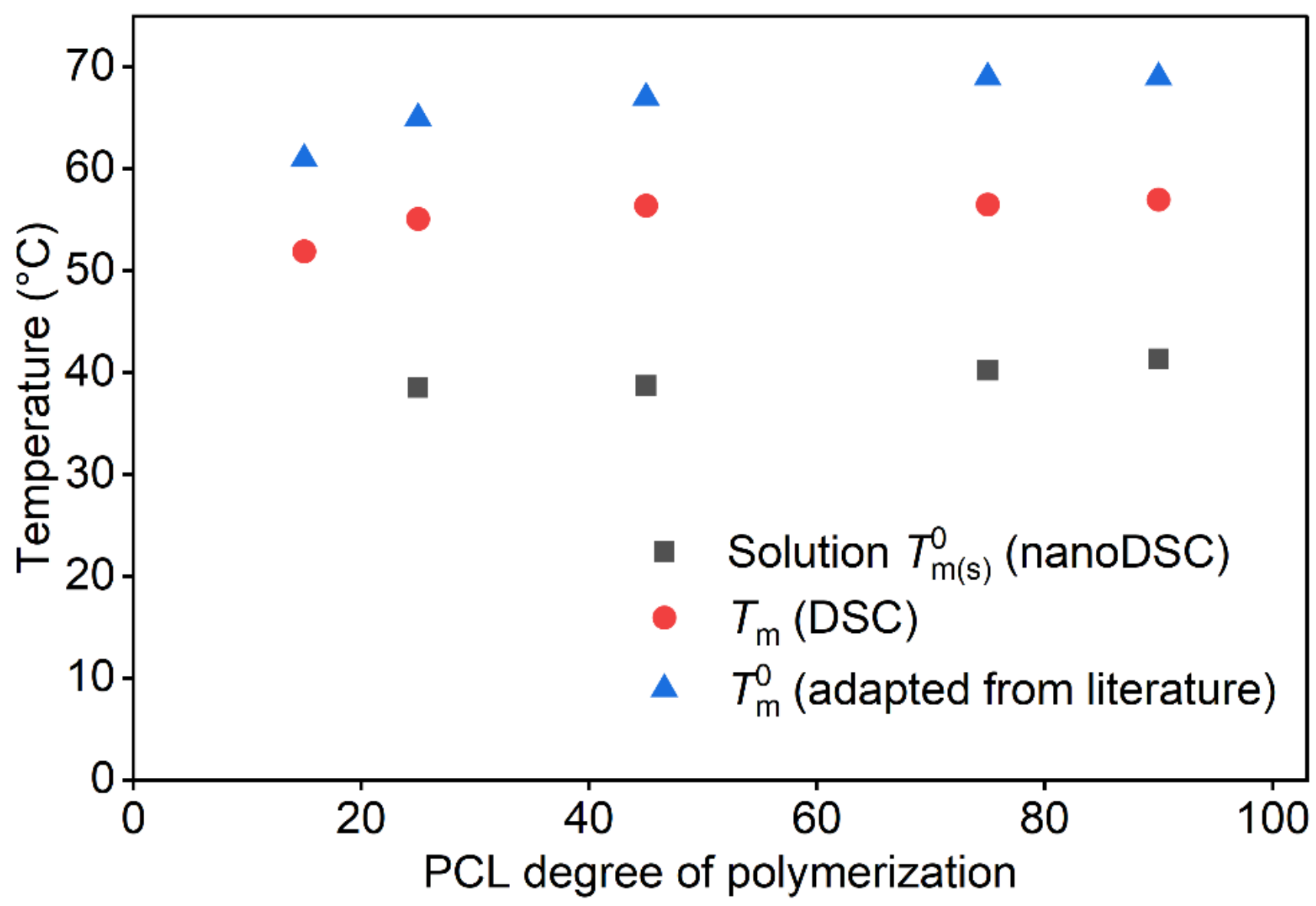

Supplementary Figure 18. Solution equilibrium melting temperatures ($T_m{}^0{}_{(s)}$) bulk melting temperatures ($T_{m,\ DSC}$), and equilibrium melting temperatures ($T_m{}^0$ adapted from literature fitting curve)[2] of different PCL chain lengths. The solution equilibrium melting temperatures were subsequently used for the Gibbs-Thomson analysis and calculations of σ_e.

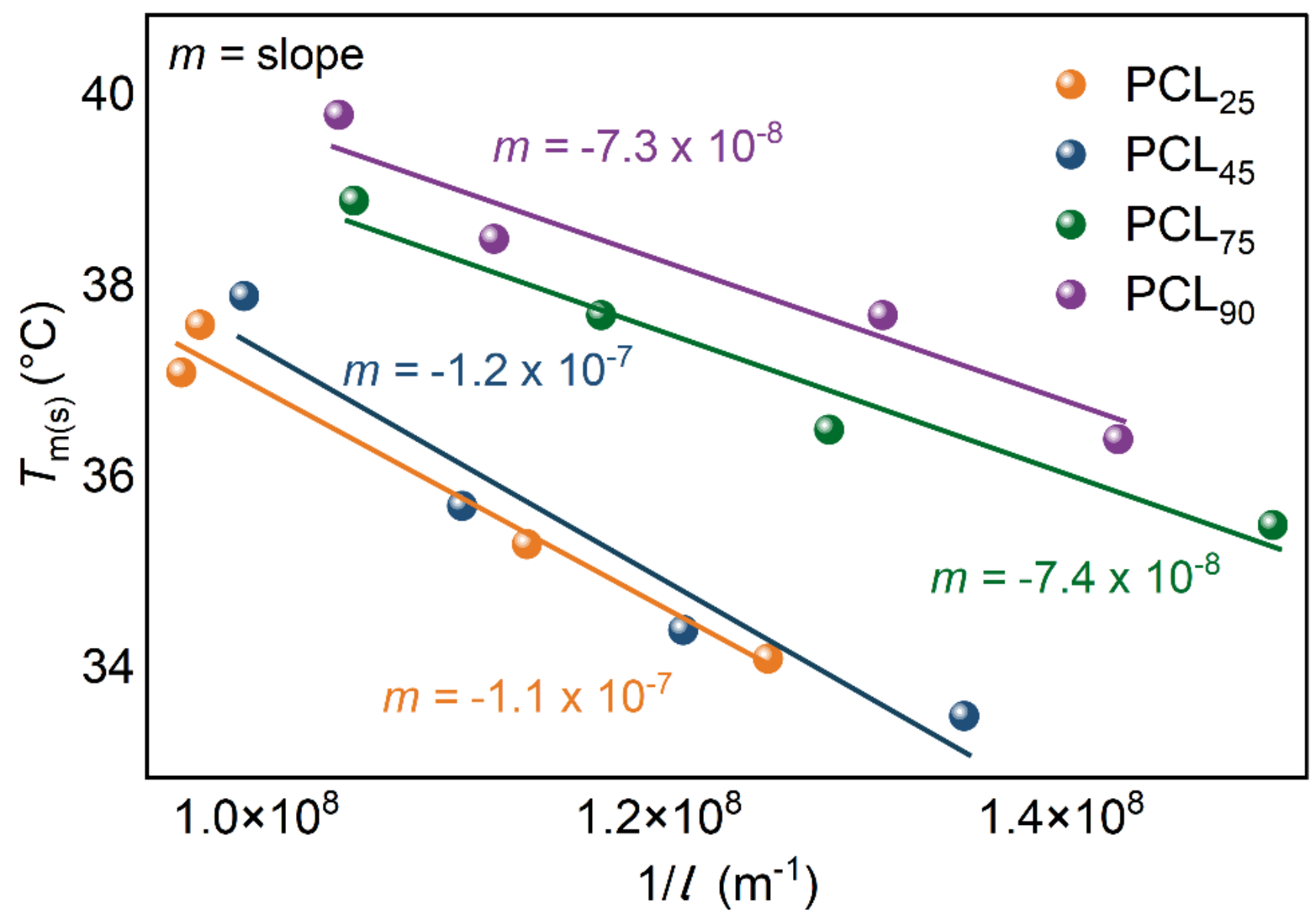

Supplementary Figure 19. Gibbs-Thomson analysis to determine surface free energies. The equilibrium melting temperature of a finite lamellar crystal depends on its thickness due to surface free energy contributions at the fold surfaces. This relationship is described by:

Supplementary Equation (1):

$$T_{m(s)} = T^0_{m(s)}\left(1 - \frac{2\sigma_e}{l\Delta H_f}\right)$$

Rearranging gives the linearized form:

$$T_{m(\mathrm{s})} = T^0_{m(\mathrm{s})} - \frac{2\sigma_e T^0_m}{l\Delta H_f} \times \frac{1}{l}$$

Thus, a plot of $T_{m(\mathrm{s})}$ versus $1/l$ yields a straight line:

$$T_{m(\mathrm{s})} = T^0_{m(\mathrm{s})} - m\left(\frac{1}{l}\right)$$

with slope:

$$m = \frac{2\sigma_e T^0_{m(\mathrm{s})}}{\Delta H_f}$$

and:

$$\sigma_e = \frac{\mathrm{m} \times \Delta H_f}{2T^0_{m(\mathrm{s})}}$$

where:

$$\Delta H_f = \Delta H^0_f \times p$$

where ΔH^0_f is the heat of fusion for 100% crystalline PCL, taken as 135 J g^{-1},[3] consistent with reported values used for crystallinity and thermodynamic studies of PCL, and p is the density of crystalline PCL, taken as 1.145 g cm^{-3}. The $T_m{}^0{}_{(s)}$ is the calculated apparent equilibrium temperatures listed in Supplementary Table 4.

Supplementary Table 5. Degree of crystallinity determination from nanoDSC melting peaks for platelets formed at differing crystallization temperatures.

Crystallization temperature T_c^* (°C)	Crystallinity $X_{c(s)}$ [a] (%)			
	PCL_{25}	PCL_{45}	PCL_{75}	PCL_{90}
-20	18	62	79	82
4	47	70	92	95
20	48	73	92	94
30	68	84	93	84

[a] Determined by nanoDSC.

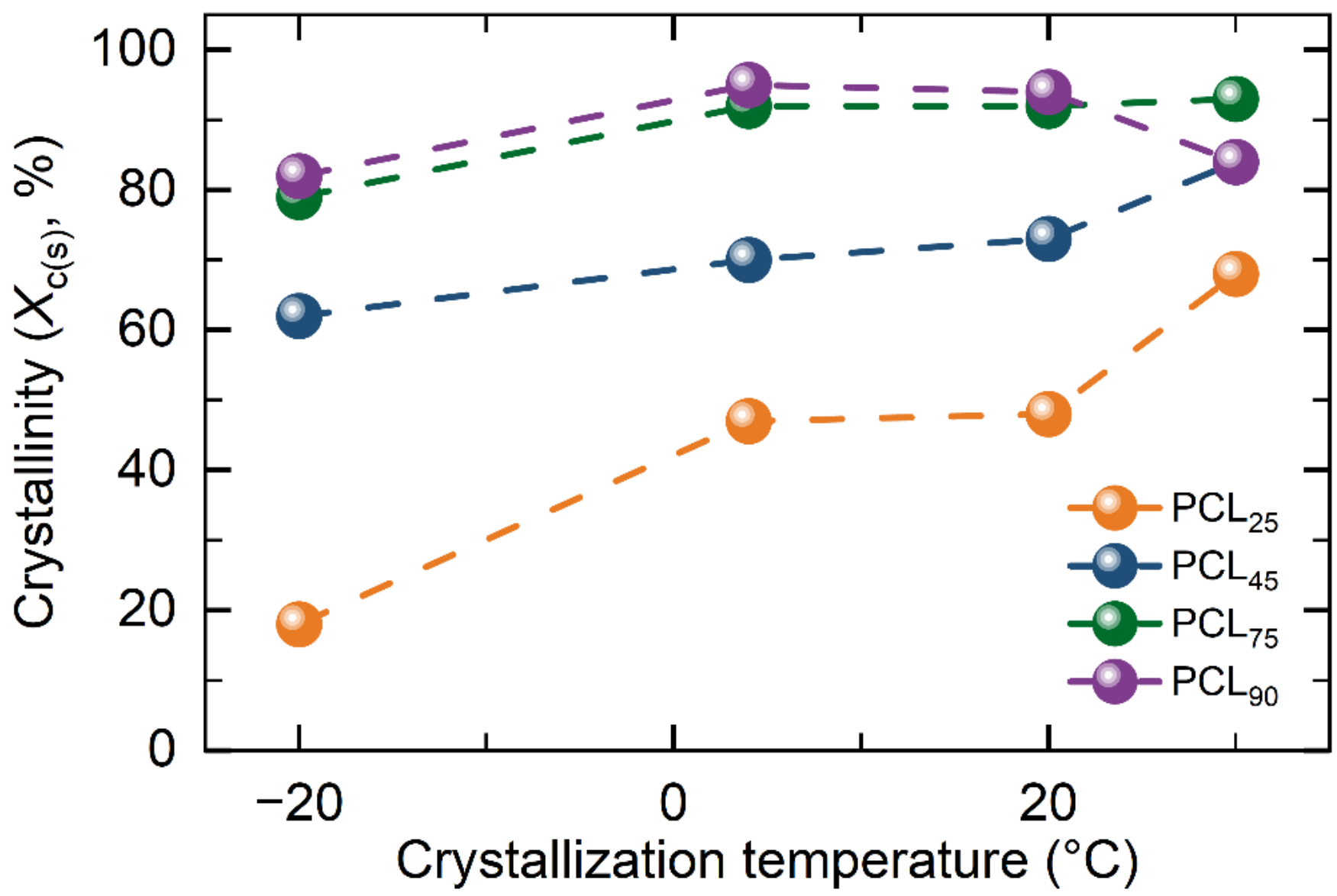

Supplementary Figure 20. Degree of crystallinity as a function of crystallization temperature.

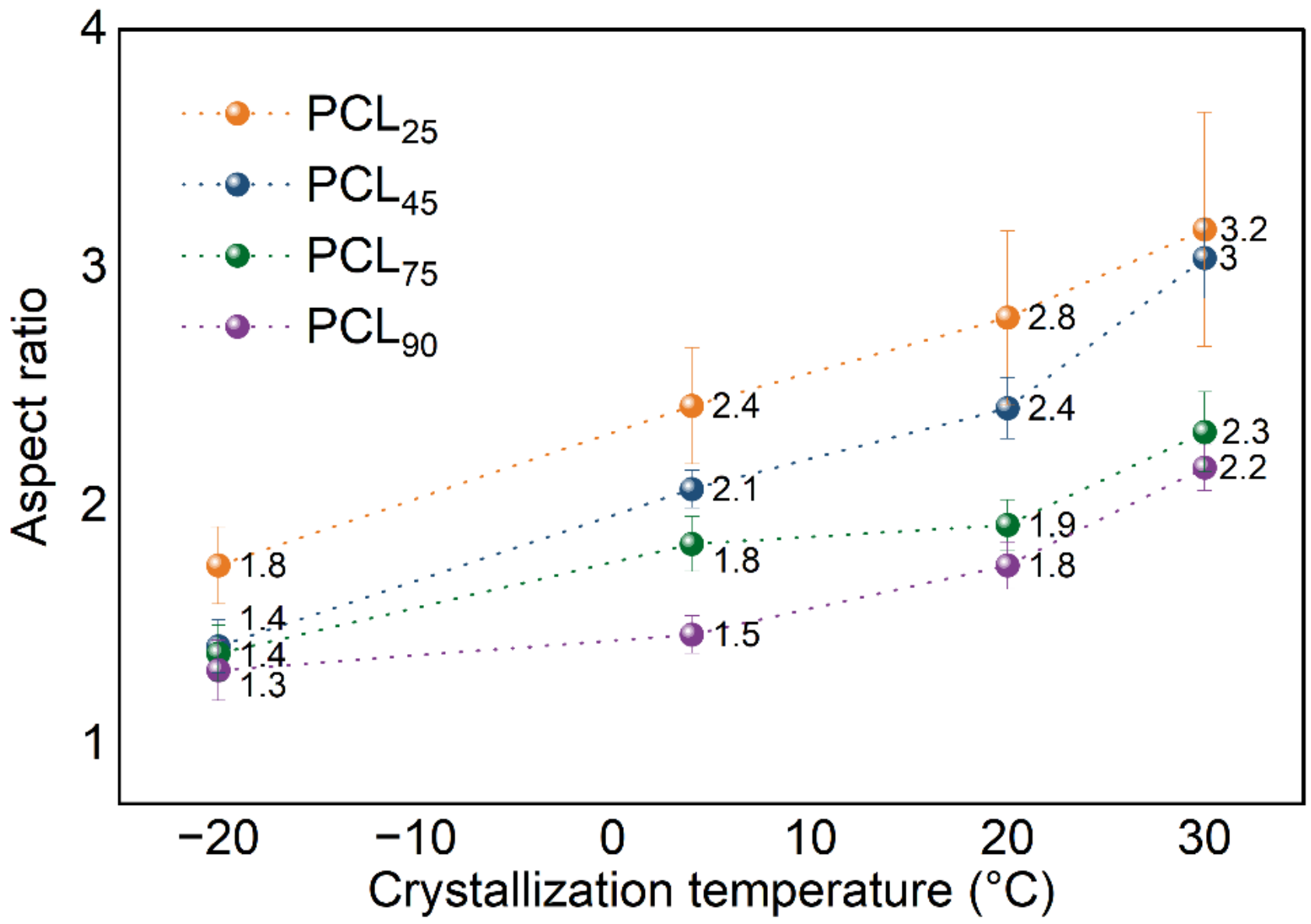

Supplementary Figure 21. Aspect ratio of pure PCL platelets formed from different chain lengths at various crystallization temperatures. The length and width of at least 100 platelets composed of PCL_{25}, PCL_{45}, PCL_{75}, and PCL_{90}, crystallized at -20, 4, 20, and 30 °C, were measured from TEM micrographs using ImageJ to calculate their corresponding aspect ratios. Data are presented as mean values ± SD (n = 100). Analysis revealed that, for a given chain length, decreasing the crystallization temperature led to a reduction in aspect ratio. This is attributed to increased supercooling, which accelerates crystallization and promotes more uniform growth along both the x and y directions. Notably, adjusting the crystallization temperature provided greater control over aspect ratio than varying the PCL chain length alone, enabling access to more extreme aspect ratios (1.3 and 3.2). This is because modifying chain length only alters the intrinsic bulk melting temperature of PCL, thereby limiting the achievable degree of supercooling. In contrast, lowering the crystallization temperature allows more direct and extensive control over supercooling. Nevertheless, the extent of aspect ratio tuning through temperature remains dependent on chain length.

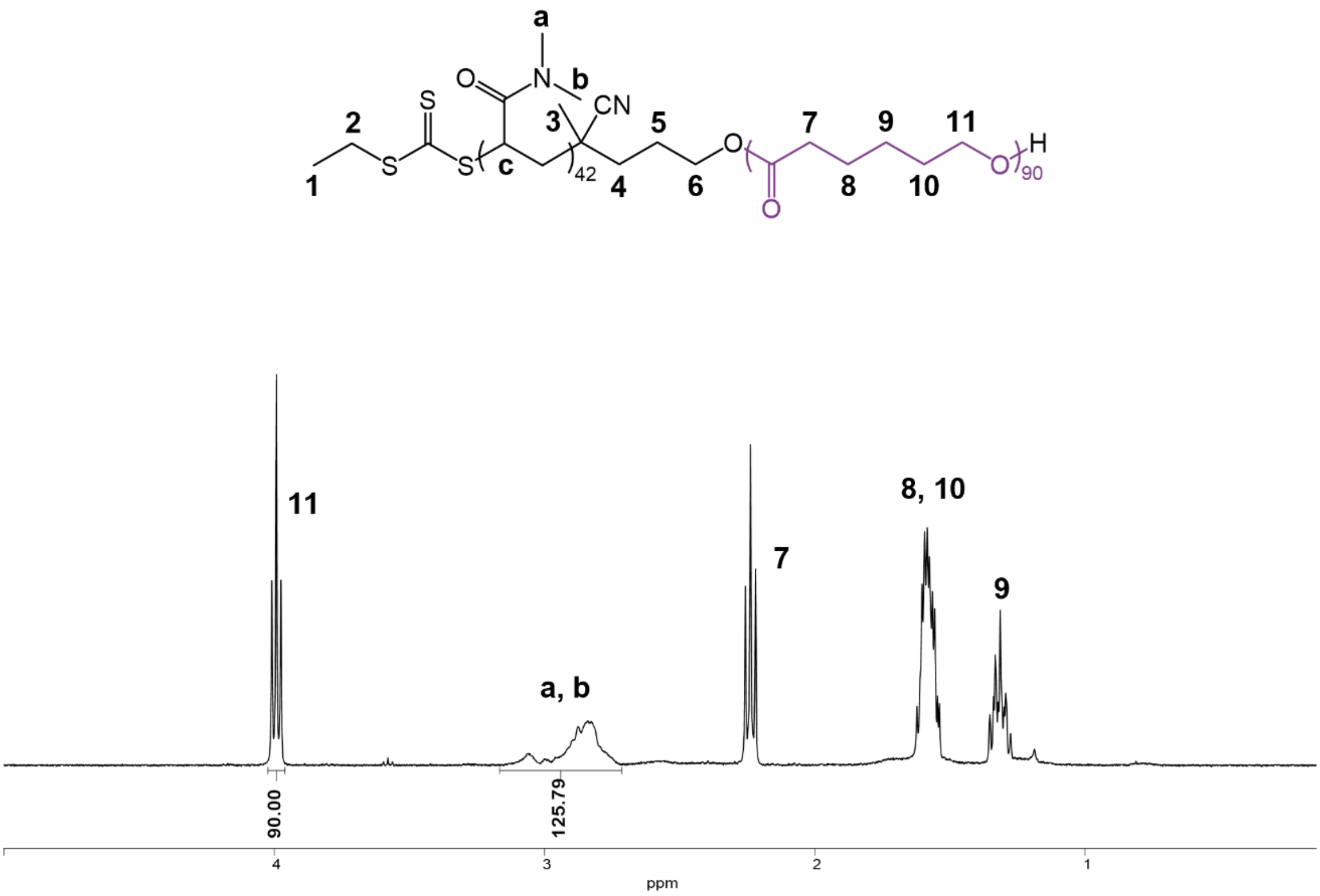

Supplementary Figure 22. ^{1}H NMR spectrum (400 MHz, $CDCl_3$) of PCL_{90}-*b*-$PDMA_{42}$.

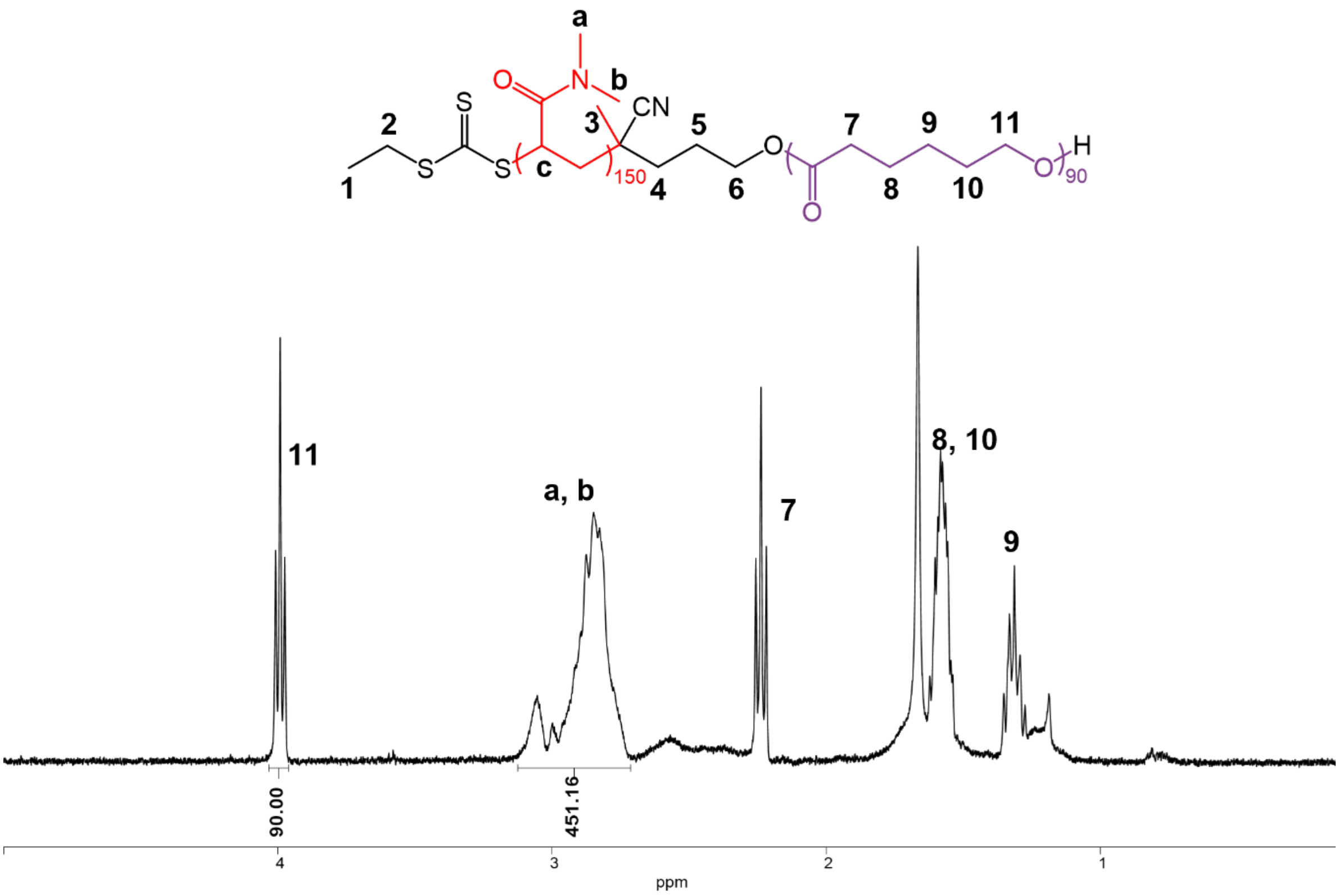

Supplementary Figure 23. ^{1}H NMR spectrum (400 MHz, $CDCl_3$) of PCL_{90}-*b*-$PDMA_{150}$.

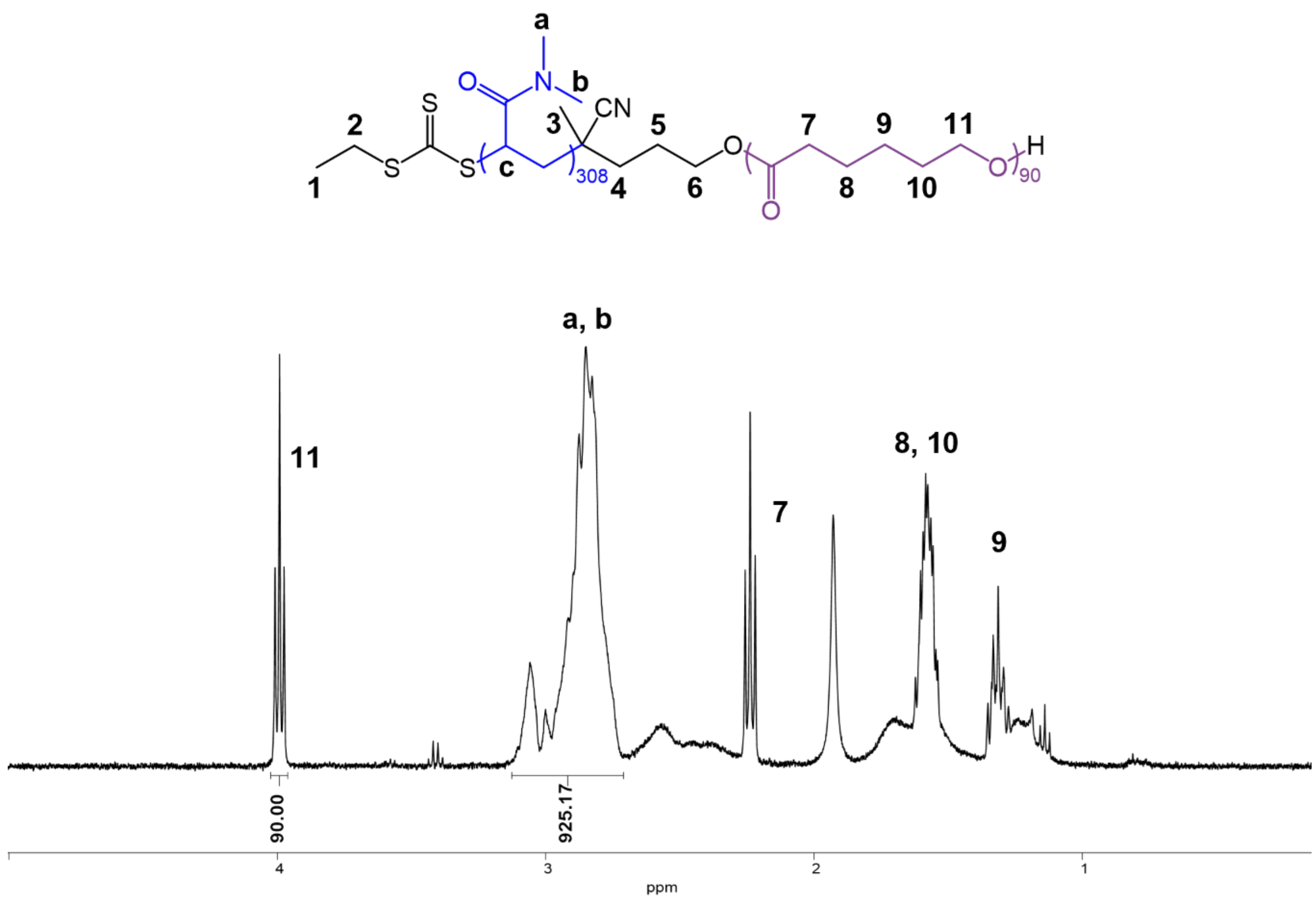

Supplementary Figure 24. ^{1}H NMR spectrum (400 MHz, $CDCl_3$) of PCL_{90}-*b*-$PDMA_{308}$.

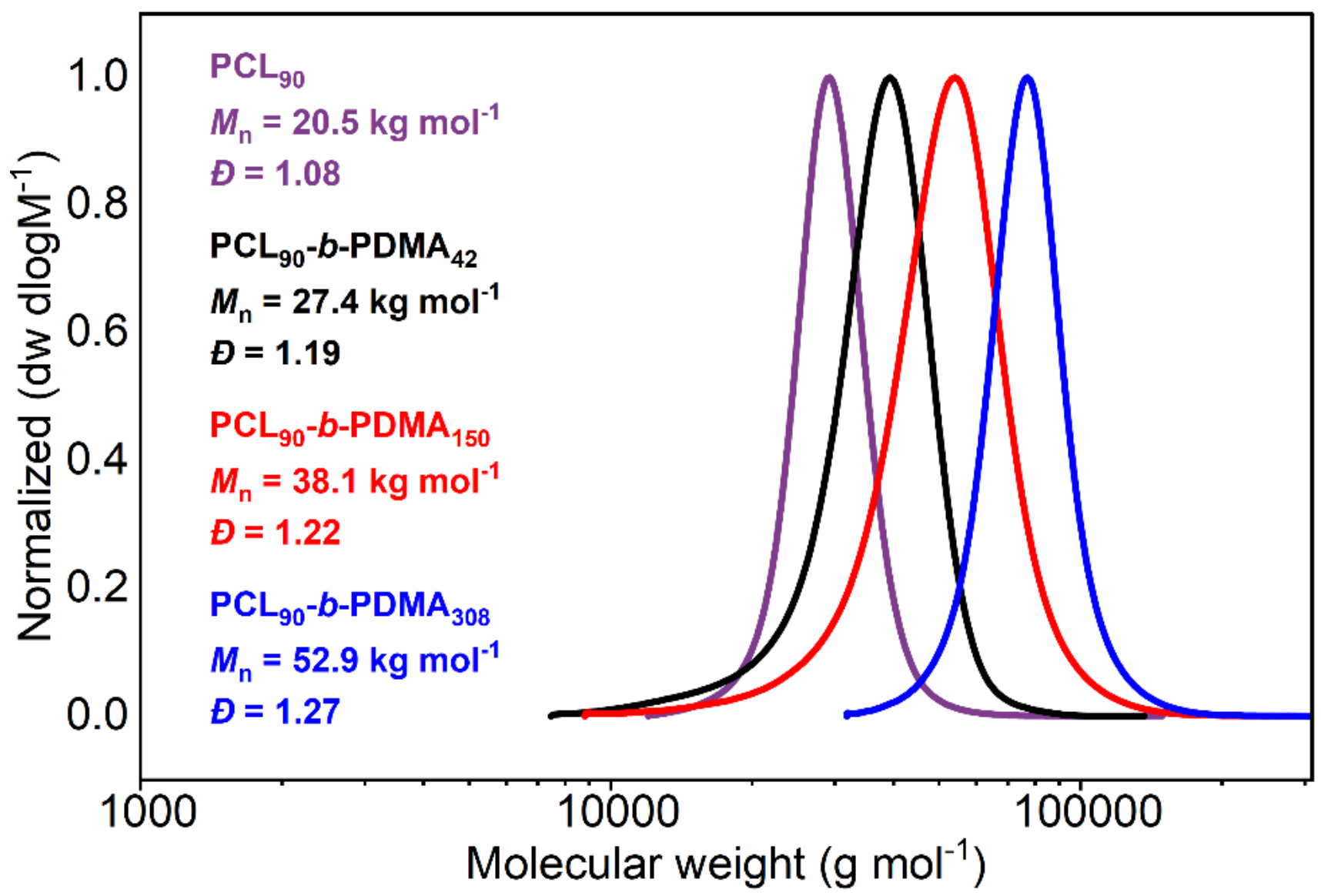

Supplementary Figure 25. Size exclusion chromatograms of PCL_{90}, PCL_{90}-*b*-$PDMA_{42}$, PCL_{90}-*b*-$PDMA_{150}$, and PCL_{90}-*b*-$PDMA_{308}$ (RI, $CHCl_3$ with 0.5% NEt_3 as an eluent against PMMA standard).

Supplementary Table 6. Molecular characterization of all the polymers used in this study.

Polymer	$M_{n, NMR}$[a] (kg mol^{-1})	$M_{n, SEC}$[b] (kg mol^{-1})	$Đ$, SEC[b]	$T_{m, DSC}$[c] (°C)	$T_{c, DSC}$[c] (°C)
PCL_{15}	2.1	4.2	1.17	-	-
PCL_{25}	3.1	6.3	1.12	55.1	36.1
PCL_{45}	5.4	12	1.09	56.4	36.8
PCL_{75}	8.8	16.6	1.07	56.5	37.4
PCL_{90}	10.5	20.5	1.08	57	37.9
PCL_{90}-*b*- $PDMA_{42}$	14.7	27.4	1.19	-	-
PCL_{90}-*b*-$PDMA_{150}$	25.4	38.1	1.22	-	-
PCL_{90}-*b*-$PDMA_{308}$	41.1	52.9	1.27	-	-

[a] Determined from ^{1}H NMR spectra.

[b] Obtained from SEC analysis.

[c] Obtained from DSC curves.

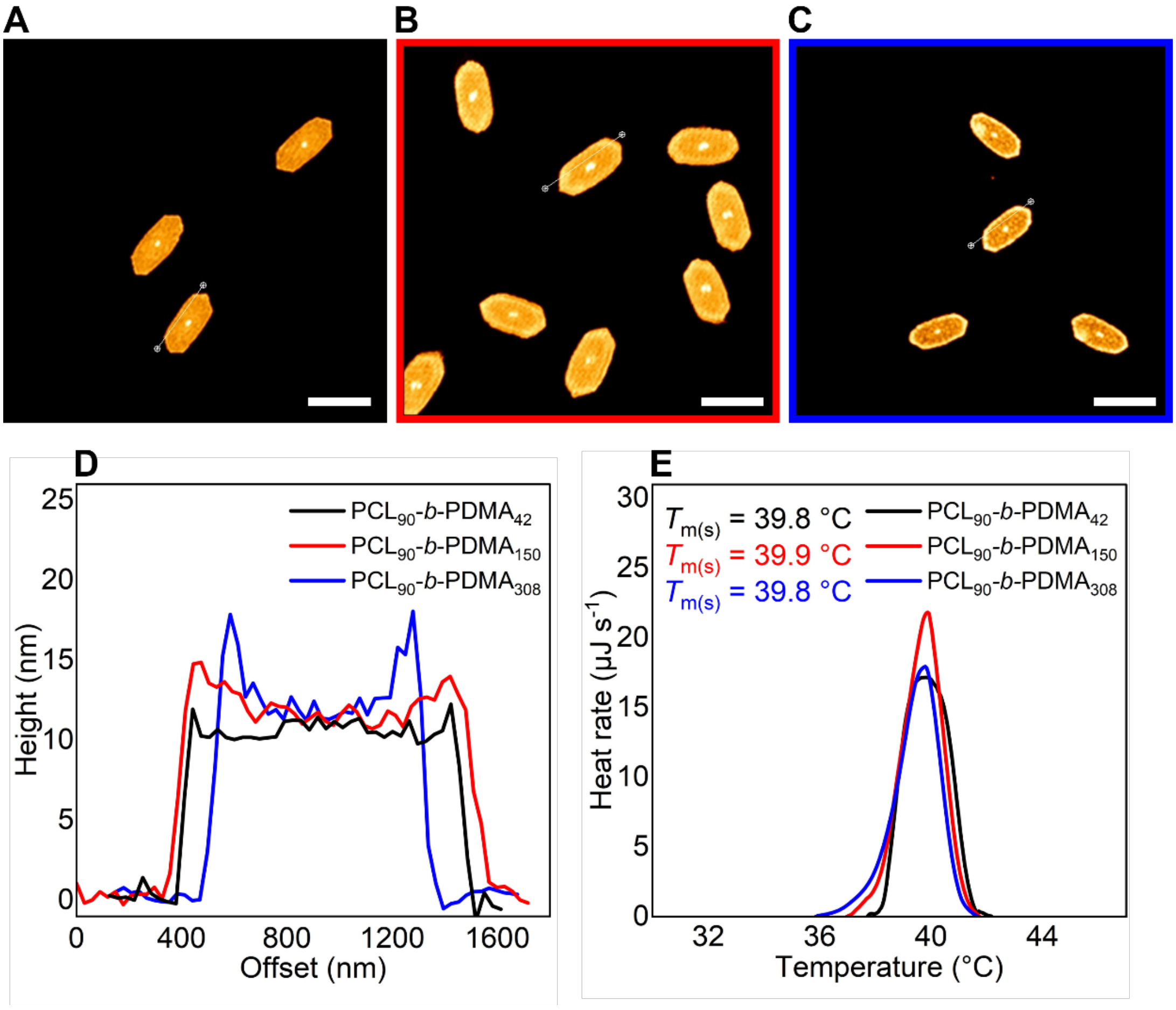

Supplementary Figure 26. Thermal analysis of PCL_{90}/PCL_{90}-*b*-PDMA platelets with varying PDMA chain lengths formed at 30 °C. AFM images of platelets prepared from PCL_{90}/PCL_{90}-*b*-PDMA with PDMA degrees of polymerization (DP) of **(a)** 42, **(b)** 150, and **(c)** 308 are shown. All scale bars are equivalent to 1 μm. **(d)** Stacked height profiles extracted from the AFM images highlight the corona contribution to platelet thicknesses across all PDMA lengths. **(e)** NanoDSC thermograms indicate no significant differences in melting temperatures among the samples.

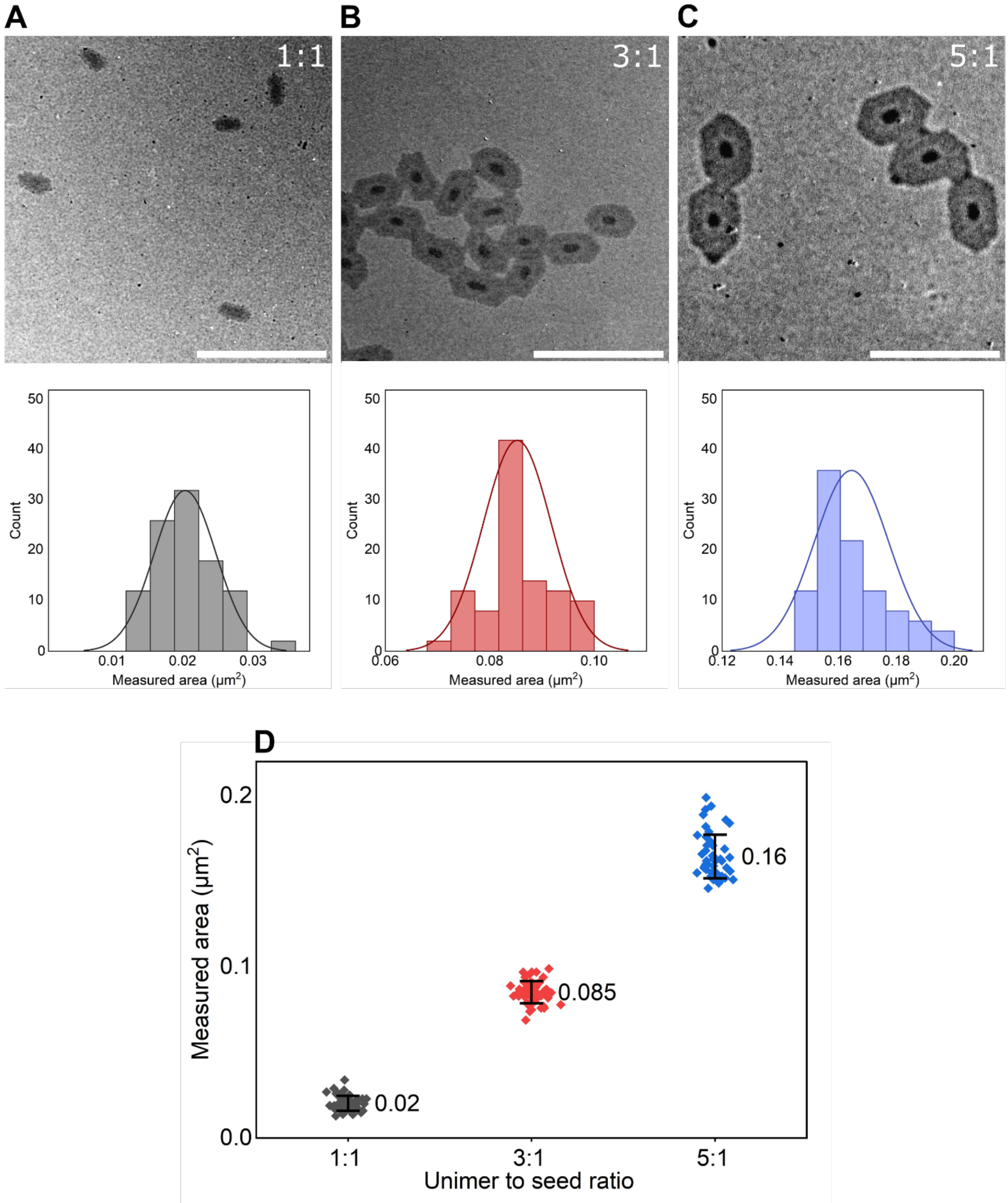

Supplementary Figure 27. Effect of unimer-to-seed ratio on the surface area of PCL_{45} platelets formed at -20 °C. TEM micrographs of PCL_{45} platelets prepared at -20 °C with unimer-to-seed ratios of **(a)** 1:1, **(b)** 3:1, and **(c)** 5:1. All scale bars are equivalent to 1 µm. The corresponding surface area distribution bar charts are displayed beneath each image. **(d)** Scatter plot comparing the measured platelet area as a function of unimer-to-seed ratio. Data are presented as mean values ± SD (n = 100).

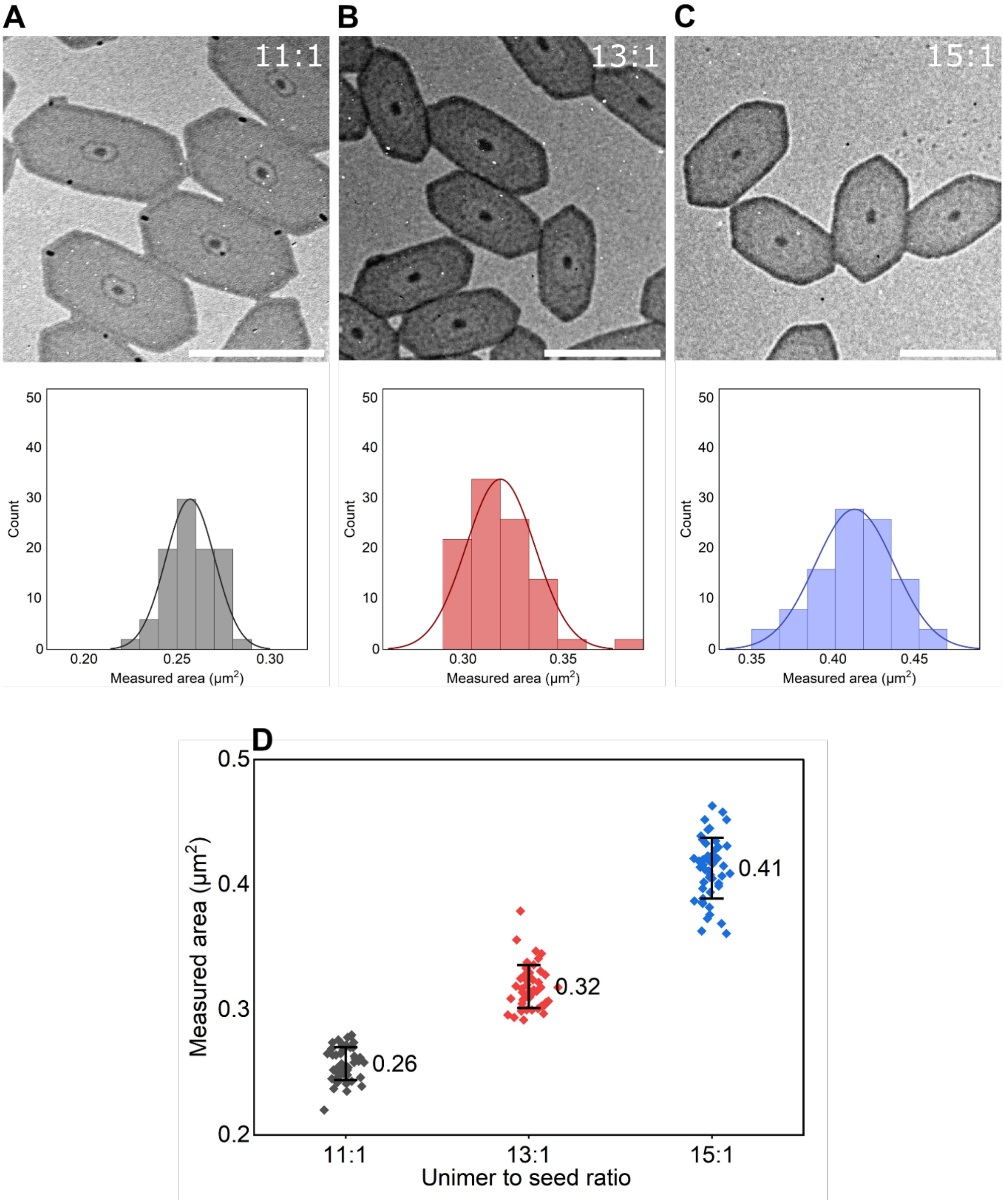

Supplementary Figure 28. Surface area analysis of epitaxial growth on PCL_{45} seed platelets using PCL_{90}/PCL_{90}-*b*-$PDMA_{150}$. TEM micrographs of PCL_{45} platelets from Supplementary Figure S23 after subsequent addition of PCL_{90}/PCL_{90}-*b*-$PDMA_{150}$ (1:1 HP:BCP 10 mg ml^{-1} unimer mixture) at a constant unimer-to-seed ratio of 10:1 and 30 °C are shown for **(a)** addition to 1:1, **(b)** 3:1, and **(c)** 5:1 PCL_{45} platelet seeds. All scale bars are equivalent to 1 μm. Surface area distribution bar charts are displayed below each TEM micrograph. **(d)** Scatter plot comparing the measured platelet areas of each sequential unimer addition. Data are presented as mean values ± SD (n = 100).

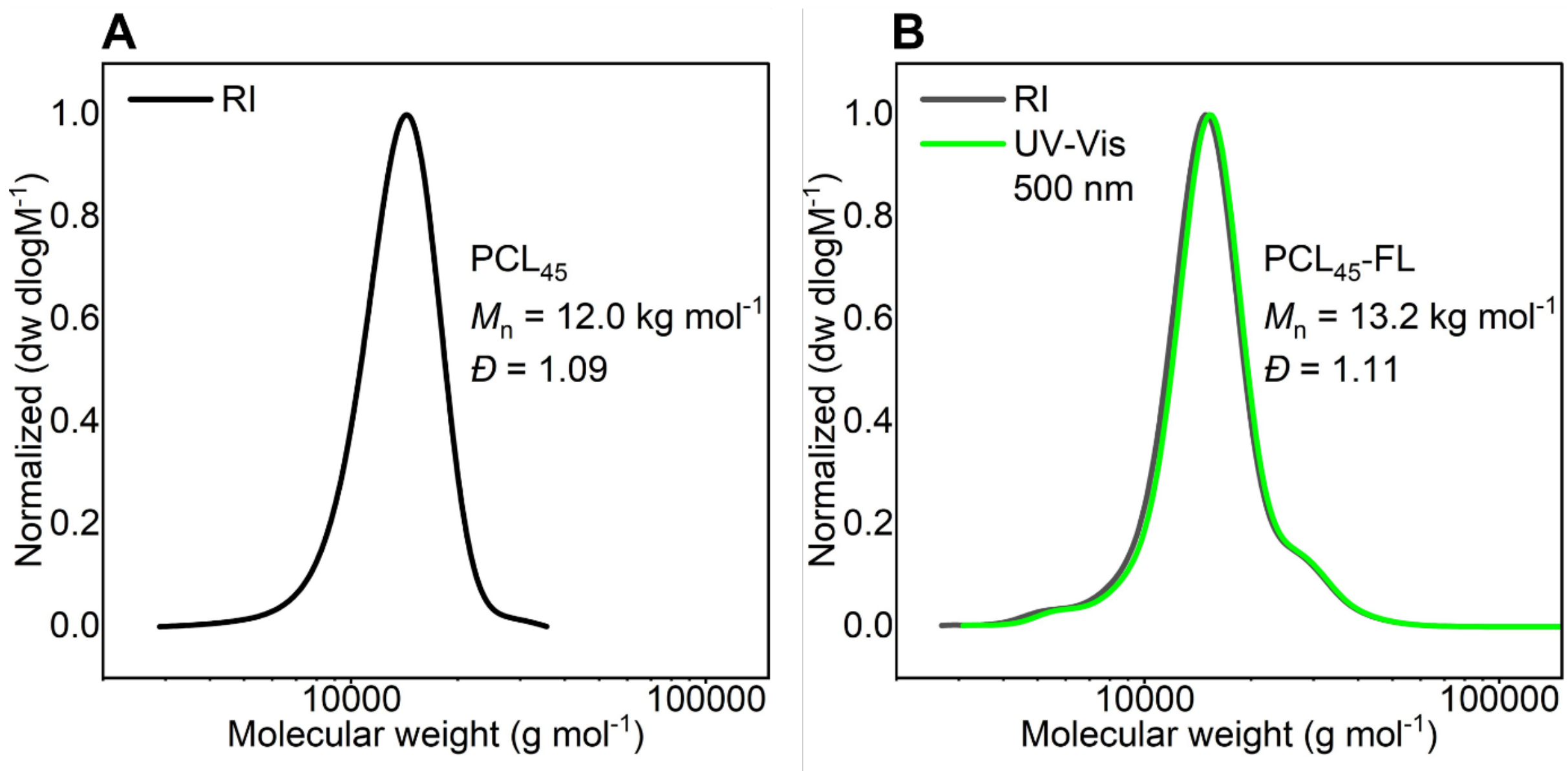

Supplementary Figure 29. Size exclusion chromatograms (RI and UV-Vis trace of 500 nm) of PCL_{45} homopolymer before **(a)** and after **(b)** coupling with fluorescent molecule BODIPY-FL ($CHCl_3$ with 0.5% NEt_3 as an eluent against PMMA standard).

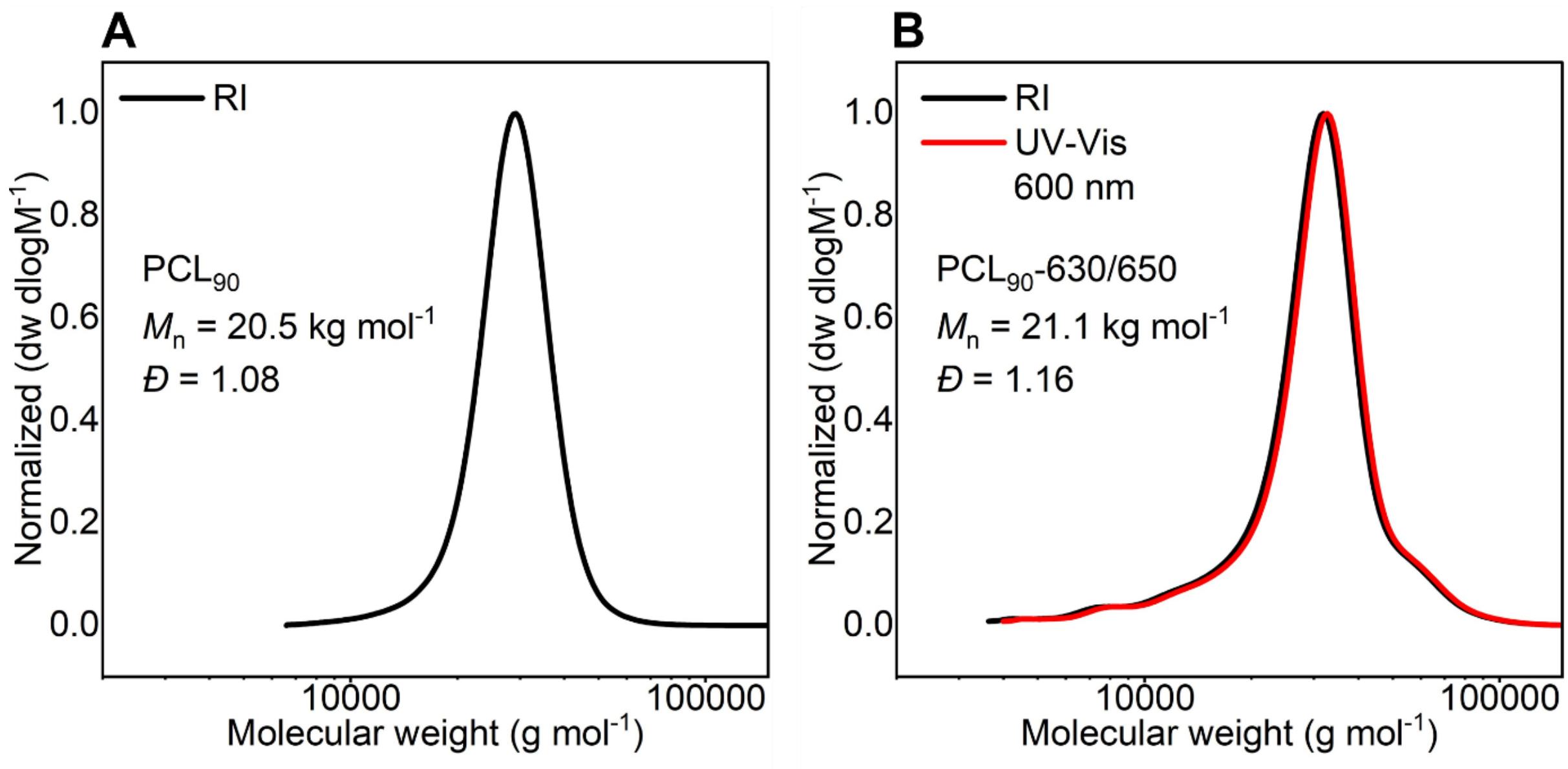

Supplementary Figure 30. Size exclusion chromatograms (RI and UV-Vis trace at 600 nm) of PCL_{90} homopolymer before **(a)** and after **(b)** coupling with fluorescent molecule BODIPY-630/650 ($CHCl_3$ with 0.5% NEt_3 as an eluent against PMMA standard).

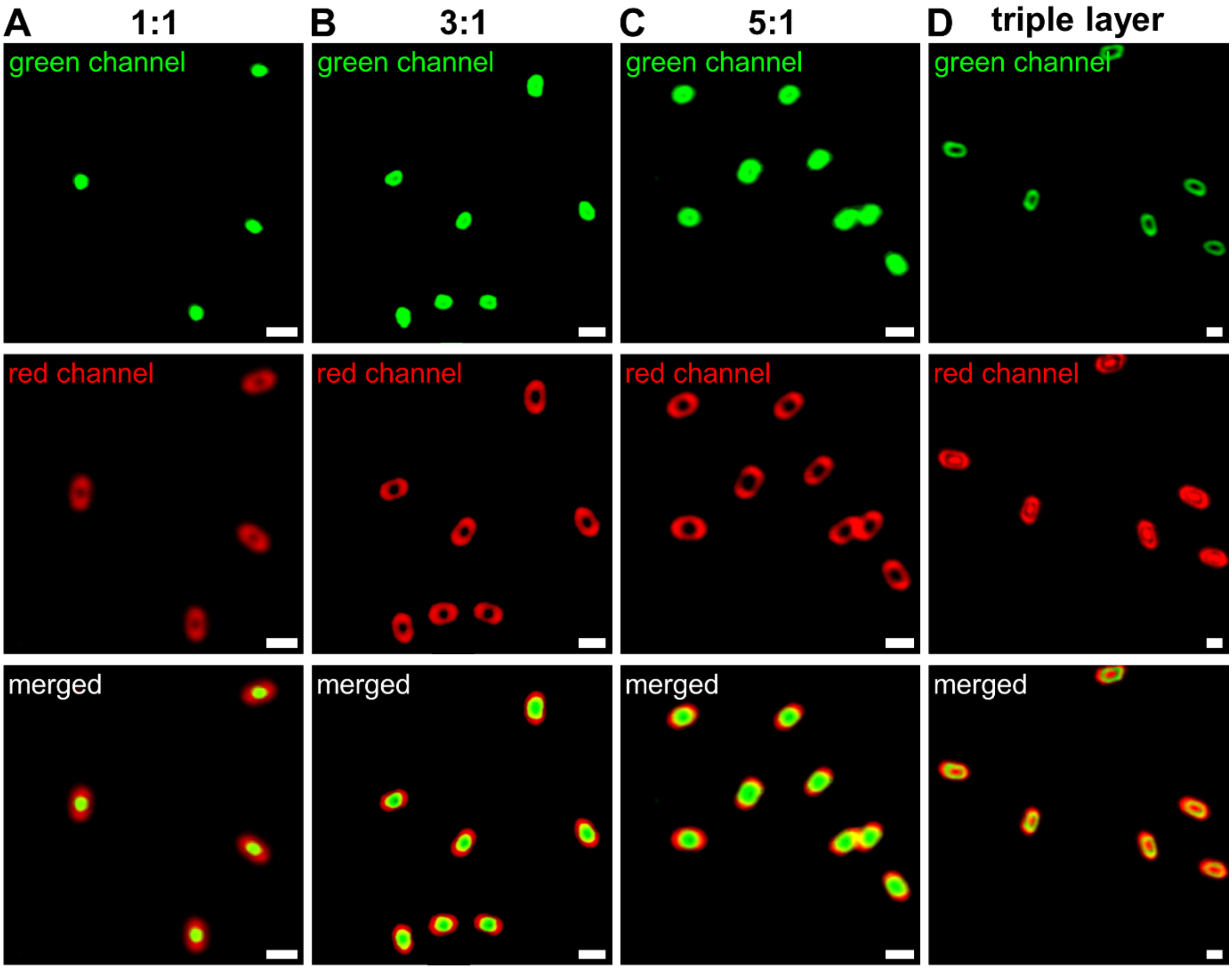

Supplementary Figure 31. Confocal fluorescence microscopy images of fluorescently labeled PCL platelets with varying layer compositions. Separate channel confocal images showing the green and red fluorescence, as well as the merged channels, are presented for **(a)** 1:1 double-layer platelets, **(b)** 3:1 double-layer platelets, **(c)** 5:1 double-layer platelets, and **(d)** triple-layer platelets. All scale bars are equivalent to 1 μm.

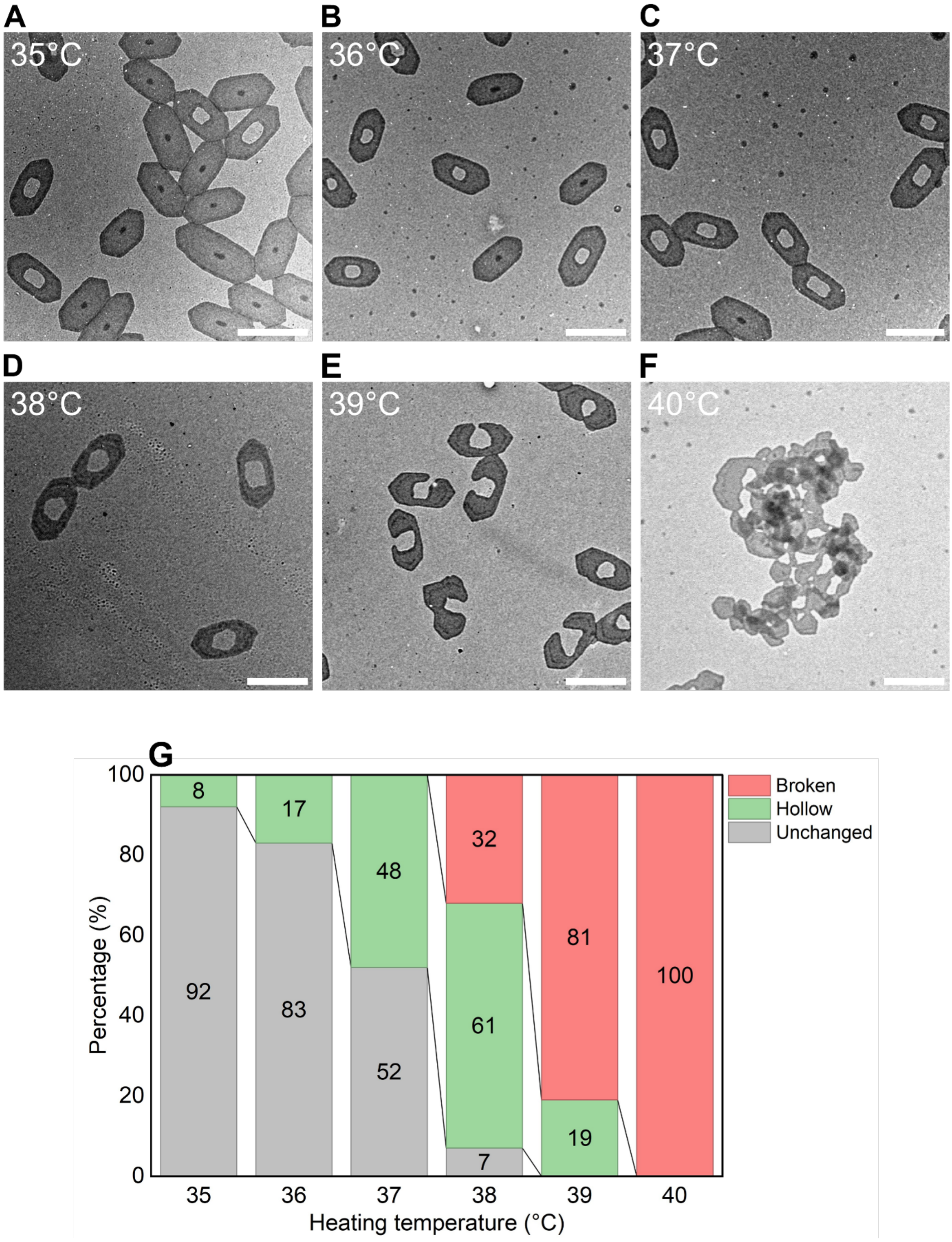

Supplementary Figure 32. Temperature-dependent structural evolution of double-layer platelets upon short-term heating. TEM images of double-layer platelets heated for 1 minute at **(a)** 35 °C, **(b)** 36 °C, **(c)** 37 °C, **(d)** 38 °C, **(e)** 39 °C, and **(f)** 40 °C are shown. All scale bars are equivalent to 1 μm. **(g)** A 100% stacked bar chart summarizes the structural outcomes following heating, categorized as broken platelets, hollow platelets, or unchanged platelets. Heating at 37 °C resulted in 48% hollow and 52% unchanged platelets, with no broken structures observed.

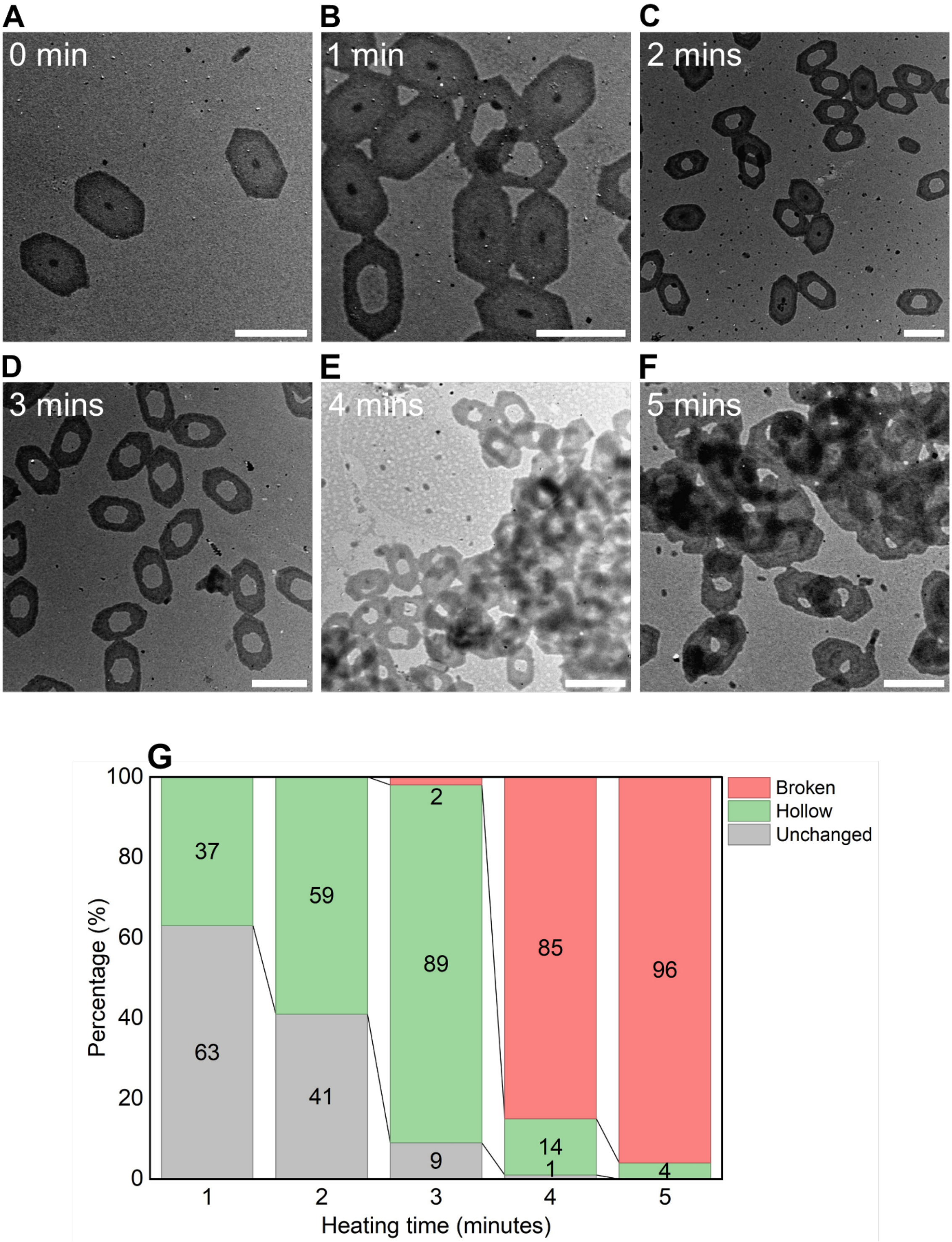

Supplementary Figure 33. Time-dependent structural evolution of double-layer platelets upon heating at 37 °C. TEM micrographs of double-layer platelets heated at 37 °C for **(a)** 0 min, **(b)** 1 min, **(c)** 2 min, **(d)** 3 min, **(e)** 4 min, and **(f)** 5 min are shown, with all scale bars equivalent to 1 μm. **(g)** A 100% stacked bar chart summarizes the corresponding structural outcomes, categorized as broken, hollow, or unchanged platelets. Progressive hollowing is observed with increasing heating time, with 3 min. yielding the highest proportion of hollow platelets (89%) and representing the optimal condition for selective hollow formation under these conditions.

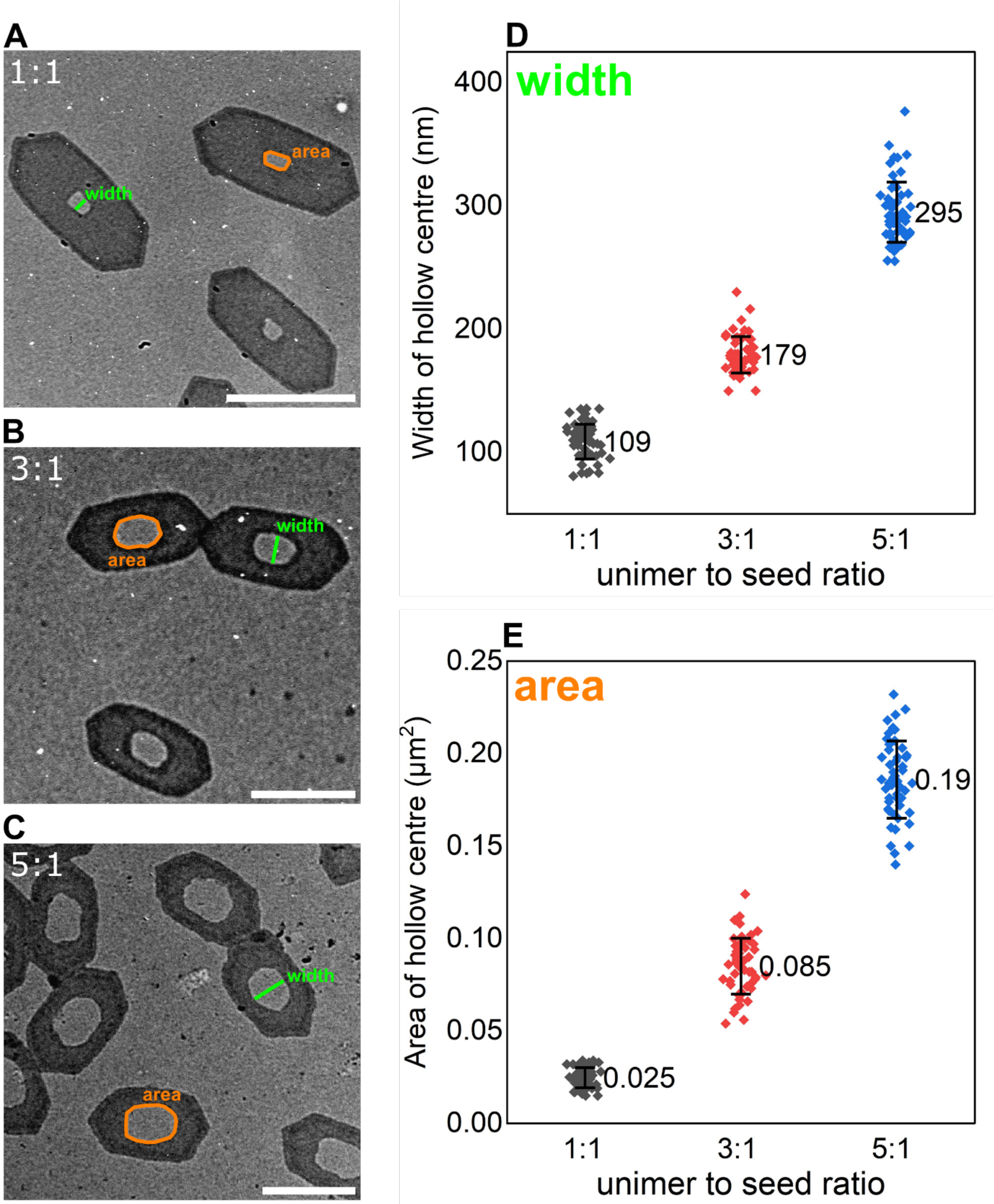

Supplementary Figure 34. Structural analysis of hollow centers in PCL platelets with varying layer ratios. TEM micrographs of platelets with hollow centers for **(a)** 1:1, **(b)** 3:1, and **(c)** 5:1 layer ratios. All scale bars are equivalent to 1 μm. **(d)** Scatter plot showing the measured width of the hollow center for each platelet type, and **(e)** scatter plot of the corresponding hollow center areas measured from TEM micrograph. Data are presented as mean values ± SD (n = 100).

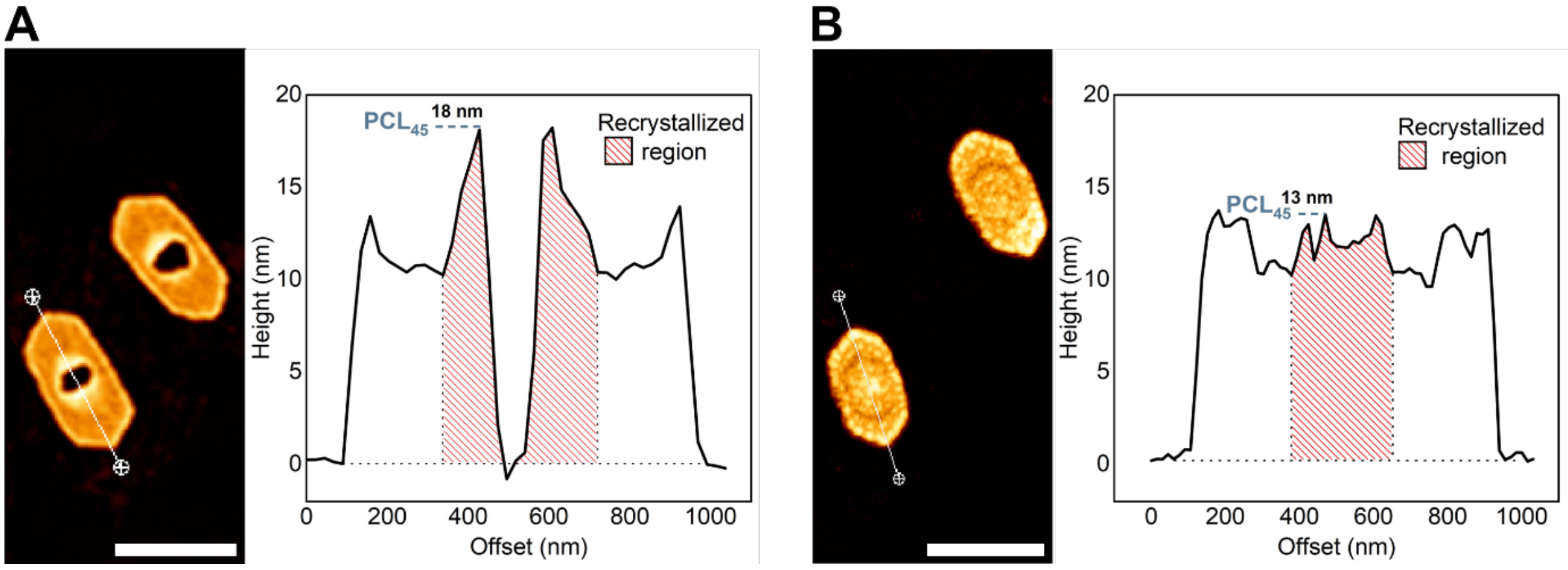

Supplementary Figure 35. AFM analysis of hollow platelet recrystallization at different temperatures. **(a)** AFM image of spatially confined hollow platelets recrystallized at 37 °C, with corresponding height profiles showing increased lamellar thickness. **(b)** AFM image of fully reformed platelets after recrystallization at -20 °C, showing reduced heights and the disappearance of the hollow structure. All scale bars are equivalent to 1 μm.

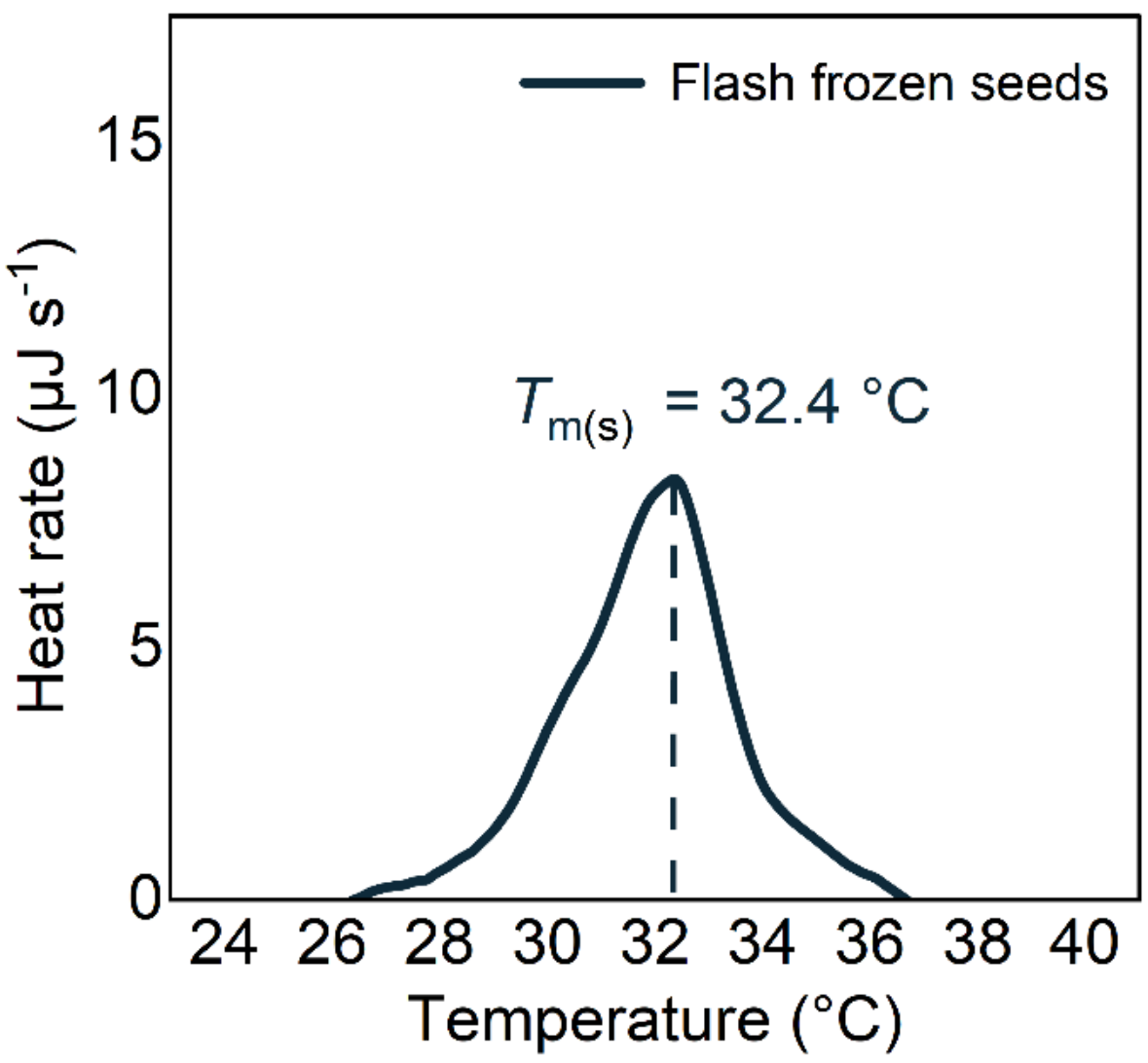

Supplementary Figure 36. Melting temperature ($T_{m(s)}$) of flash-frozen seeds. DSC heat flow trace of flash-frozen PCL_{50}-*b*-$PDMA_{200}$ seeds, prepared using a flow reactor setup. Owing to the flash-frozen nature of the seeds, together with their limited annealing history at 30 °C, the isolated seeds in solution display relatively lower melting temperatures than the platelets prepared in this study. As such, during the melting process, these seeds melt and contribute to the pool of PCL chains available for subsequent recrystallization. However, when an adjacent lamellar domain has been grown on the seeds (*i.e.*, in triple layer platelets, where the seeds are initially protected by a thick PCL_{90}/PCL_{90}-*b*-$PDMA_{150}$ crystalline domain), their apparent thermal stability is increased as the seeds are no longer directly exposed.

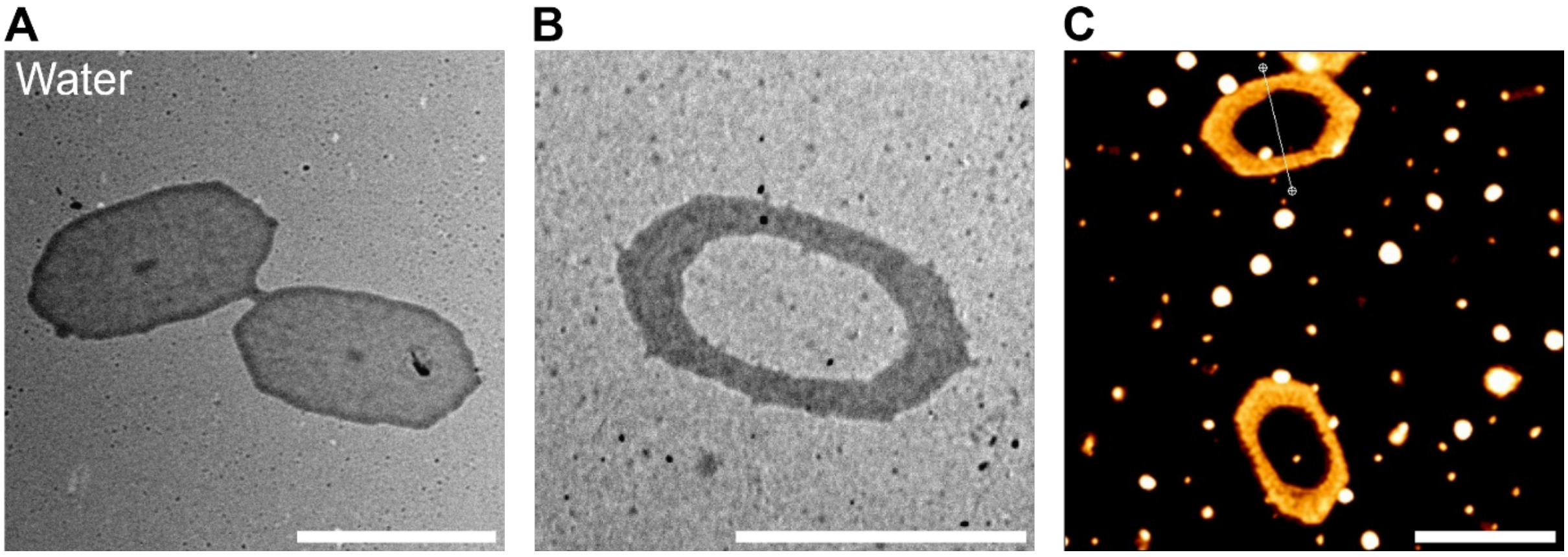

Supplementary Figure 37. Melting process of double layer platelets transferred to water. **(a)** TEM micrograph of double layer platelets after transfer to water by dialysis. The platelets retained their stability in water and were able to undergo selective melting of the inner layer at an elevated temperature (50 °C), resulting in the formation of hollow platelets, as characterized by **(b)** TEM, and **(c)** AFM. Interestingly, following melting in water, the released PCL chains rearranged into spherical particles that are visible in both the TEM and AFM images. All scale bars are equivalent to 1 μm.

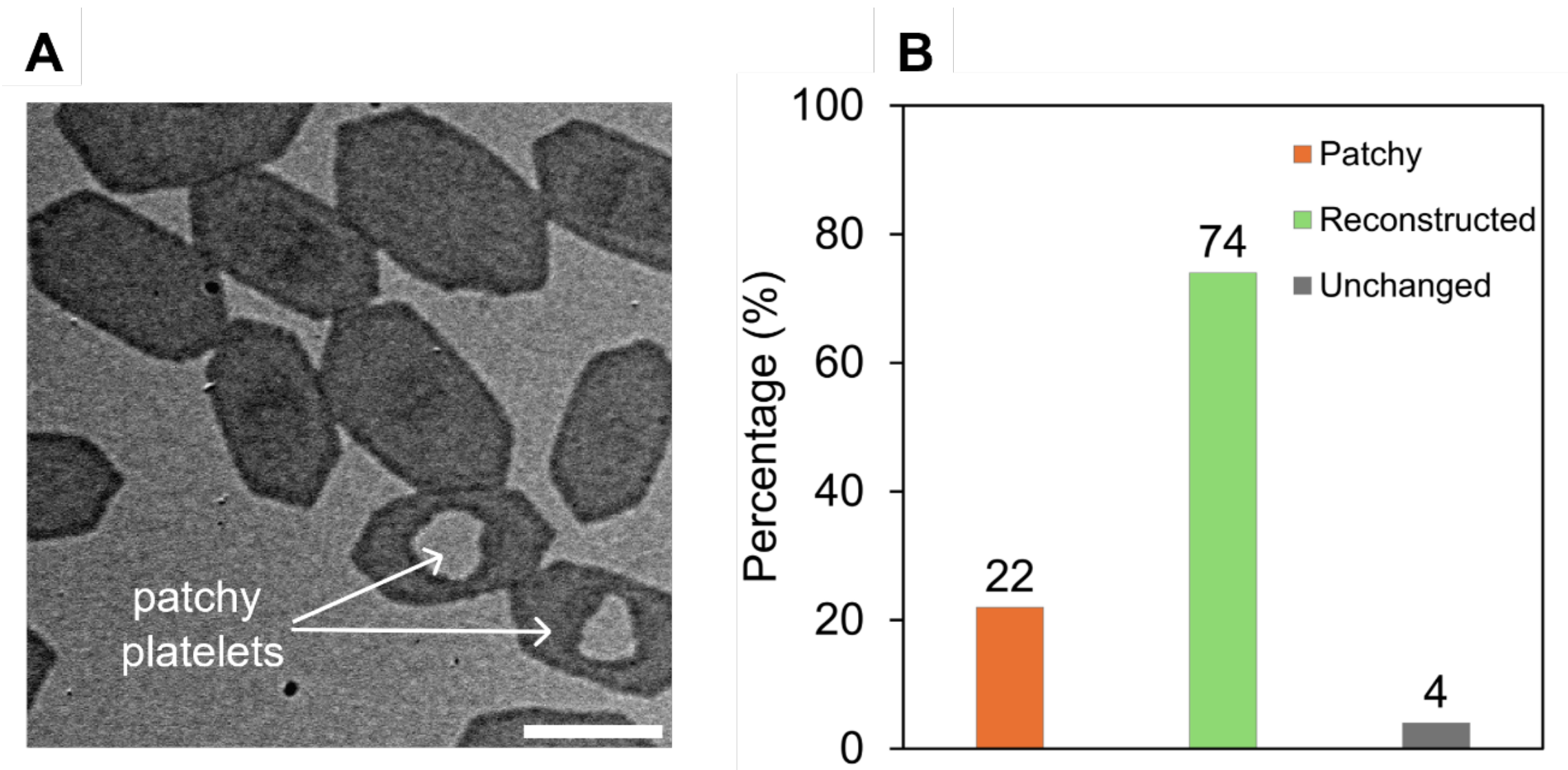

Supplementary Figure 38. (a) TEM micrograph showing the presence of patchy platelets alongside reconstructed platelets during recrystallization. Under static conditions, recrystallization at -20 °C promotes lateral growth of PCL chains within the hollow interior, effectively restoring the original platelet architecture. However, in some cases, insufficient chains are available to fully close the hollow interior, resulting in the formation of patchy platelets. All scale bars are equivalent to 1 μm. **(b)** Population distribution of patchy, reconstructed, and unchanged platelets (n = 100 platelets).

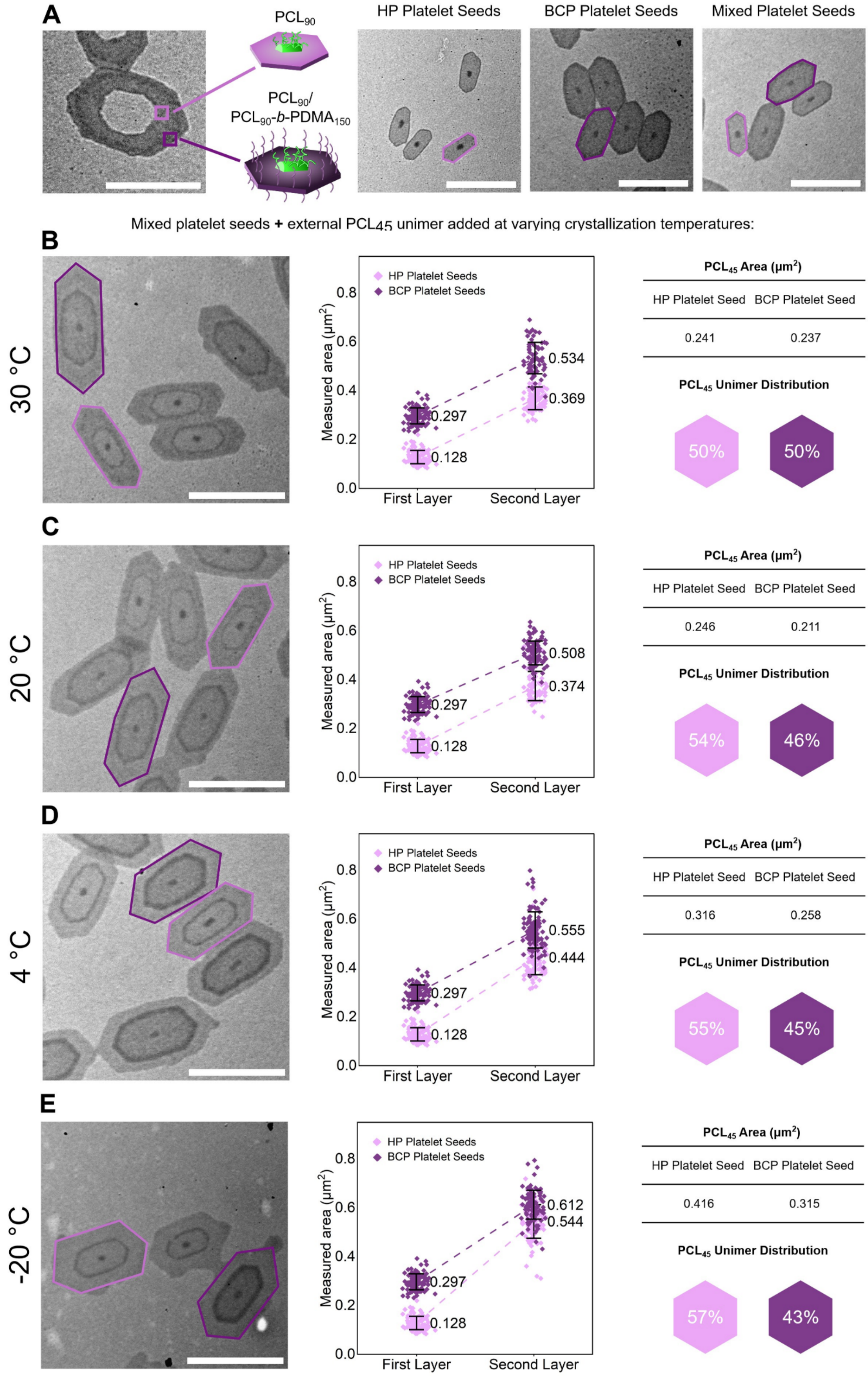
A
PCL90
PCL90/
PCL90-b-PDMA150
HP Platelet Seeds
BCP Platelet Seeds
Mixed Platelet Seeds
Mixed platelet seeds + external PCL45 unimer added at varying crystallization temperatures:
B
30 °C
HP Platelet Seeds
BCP Platelet Seeds
Measured area (μm2)
0.534
0.369
0.297
0.128
First Layer
Second Layer
PCL45 Area (μm2)
HP Platelet Seed
BCP Platelet Seed
0.241
0.237
PCL45 Unimer Distribution
50%
50%
C
20 °C
0.508
0.374
0.297
0.128
0.246
0.211
54%
46%
D
4 °C
0.555
0.444
0.297
0.128
0.316
0.258
55%
45%
E
-20 °C
0.612
0.544
0.297
0.128
0.416
0.315
57%
43%

Supplementary Figure 39. Competition study for crystallization. **(a)** To emulate the distinct environments of the interior and exterior of hollow platelets, two types of platelet seeds were synthesized. Owing to differences in crystallization kinetics between PCL_{90} homopolymer (HP) and PCL_{90}-*b*-$PDMA_{150}$ (BCP), growth of the second layer of double layer platelets at 30 °C, results in HP crystallizing more rapidly, yielding an interior predominantly composed of HP and an exterior enriched in BCP. A solution of PCL_{45} unimer was then added to a 1:1 mixture of HP and BCP platelet seeds and crystallized at **(b)** 30 °C, **(c)** 20 °C, **(d)** 4 °C, and **(e)** -20 °C. Platelet areas were measured using ImageJ and plotted as scatter plots. The difference in area between the resulting two-layer platelets and the original platelet seeds were used to determine the area generated by PCL_{45} crystallization. From these values, the distribution of PCL_{45} unimer between HP and BCP seeds was calculated to assess any preferential crystallization behavior. All scale bars are equivalent to 1 µm. Data are presented as mean values ± SD (n = 100).

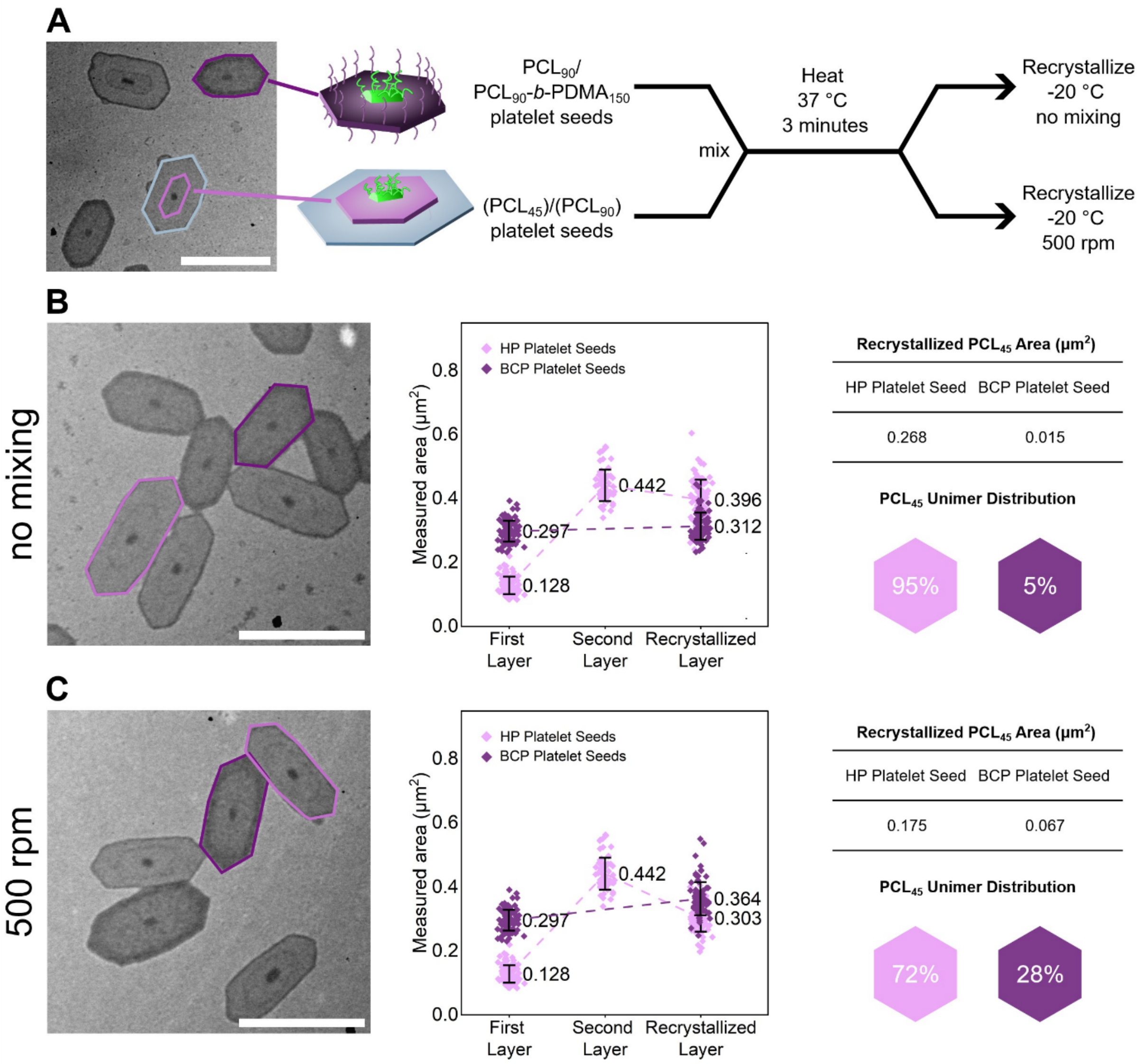

Supplementary Figure 40. Thermally labile outer layer recrystallization and competition study on HP platelet seeds. **(a)** Schematic and TEM micrograph of HP platelet seeds coated with a thermally labile PCL_{45} outer layer prior to heating, mixed with BCP platelet seeds. Samples were heated at 37 °C for 3 minutes to selectively melt the outer PCL_{45} layer and subsequently cooled to -20 °C to induce recrystallization. Recrystallization was performed under **(b)** static and **(c)** stirring at 500 rpm conditions to assess the effect of local chain enrichment on growth behavior. Scatter plots of platelet areas were used to quantify the formation of new recrystallized material, from which the distribution of PCL_{45} unimer between HP and BCP seeds was determined to assess preferential recrystallization. All scale bars are equivalent to 1 μm. Data are presented as mean values ± SD (n = 100).

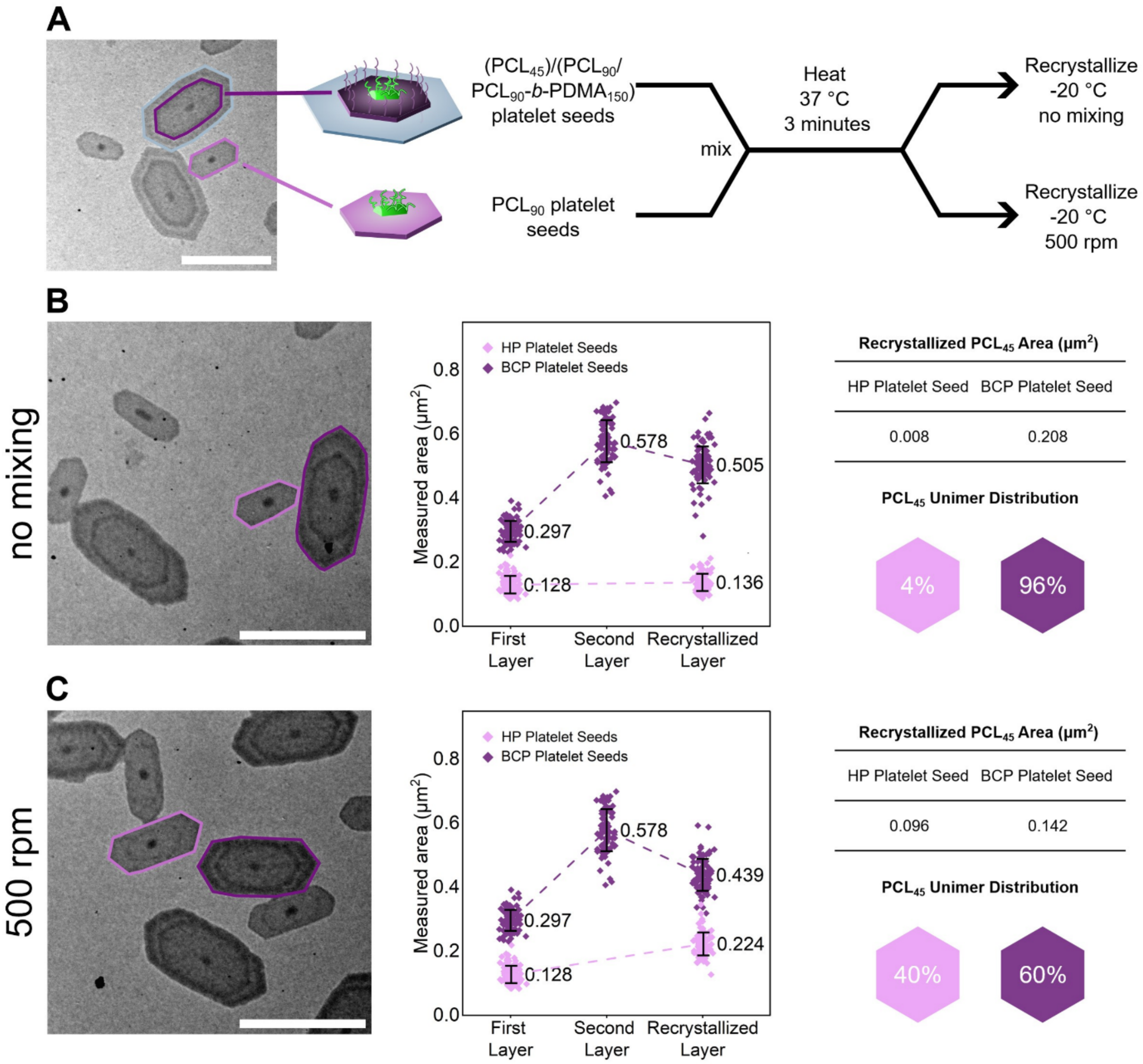

Supplementary Figure 41. Thermally labile outer layer recrystallization and competition study on BCP platelet seeds. **(a)** Schematic and TEM micrograph of BCP platelet seeds coated with a thermally labile PCL_{45} outer layer prior to heating, mixed with HP platelet seeds. Samples were heated at 37 °C for 3 minutes to selectively melt the outer PCL_{45} layer and subsequently cooled to -20 °C to induce recrystallization. Recrystallization was performed under **(b)** static and **(c)** stirring at 500 rpm conditions to assess the effect of local chain enrichment on growth behavior. Scatter plots of platelet areas were used to quantify the formation of new recrystallized material, from which the distribution of PCL_{45} unimer between HP and BCP seeds was determined to assess preferential recrystallization. All scale bars are equivalent to 1 μm. Data are presented as mean values ± SD (n = 100).

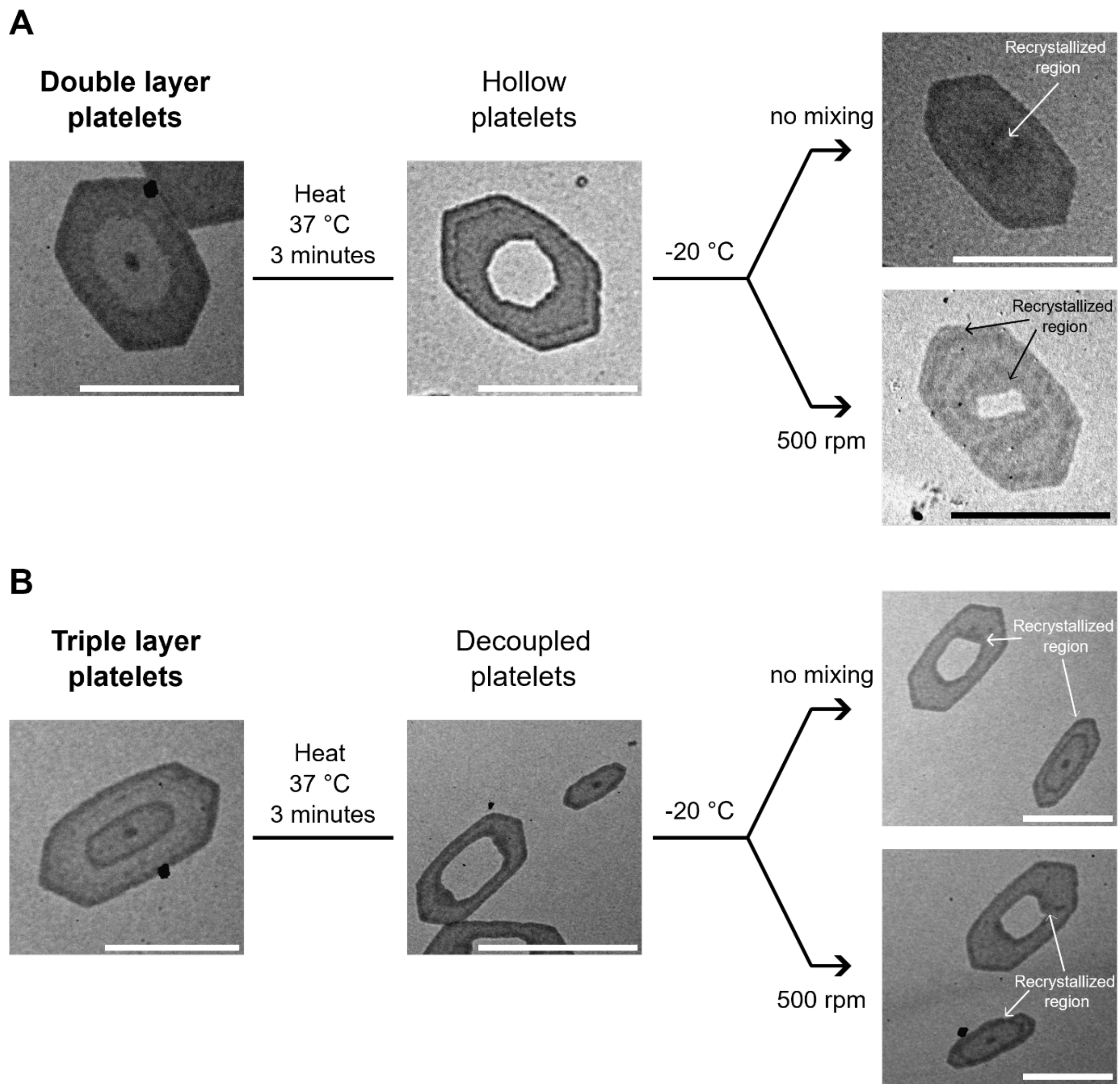

Supplementary Figure 42. Effect of stirring on recrystallization behavior of hollow and spontaneously decoupling platelets. **(a)** TEM micrographs showing double-layer platelets, their conversion to hollow platelets by selective melting, and subsequent recrystallization under static and stirred (500 rpm) conditions. Interestingly, stirring does in fact direct a proportion of recrystallization to the exterior, and the interior void is not completely filled. **(b)** TEM micrographs showing triple layer platelets, their conversion to decoupling platelets, and subsequent recrystallization under static and stirred (500 rpm) conditions. Notably, the unimer distribution in decoupling platelets differ from that of hollow platelets, as two entities contain surviving sub-critical nuclei with a templating influence; as such, recrystallization on the exterior of the hollow entity appears to be limited. All scale bars are equivalent to 1 μm.

Supplementary References

1. Arno MC, Inam M, Coe Z, Cambridge G, Macdougall LJ, Keogh R*, et al.* Precision epitaxy for aqueous 1D and 2D poly(ε-caprolactone) assemblies. *J Am Chem Soc* 2017, **139**(46)**:** 16980-16985.

2. Fernández-Tena A, Pérez-Camargo RA, Coulembier O, Sangroniz L, Aranburu N, Guerrica-Echevarria G*, et al.* Effect of molecular weight on the crystallization and melt memory of poly(ε-caprolactone) (PCL). *Macromolecules* 2023, **56**(12)**:** 4602-4620.

3. Bittiger H, Marchessault RH, Niegisch WD. Crystal structure of poly-[epsilon]-caprolactone. *Acta Cryst B* 1970, **26**(12)**:** 1923-1927.
