# Supplementary material for: Thermally programmed disassembly and reconstruction of 2D polymer platelets via melting-governed crystallization-driven self-assembly
Source: Nat Commun. 2026 Oct 3;17:11304. doi: 10.1038/s41467-026-77973-4 (PMC13724731; doi:10.1038/s41467-026-77973-4)
Supplement: Supplementary file 2 [file 41467_2026_77973_MOESM2_ESM.pdf]

nature portfolio

Peer Review File

Thermally programmed disassembly and reconstruction of 2D polymer platelets via melting-governed crystallization-driven self-assembly

Corresponding Author: Dr Maria Arno

This file contains all reviewer reports in order by version, followed by all author rebuttals in order by version.

Version 0:

Reviewer comments:

Reviewer #1

(Remarks to the Author)
This manuscript presents a sophisticated study on the thermal properties of 2D polymer platelets, effectively bridging the gap between classical polymer physics and solution-state crystallization-driven self-assembly (CDSA). The authors demonstrate that the melting temperature (Tm) of solution-grown crystals can be utilized as a precise design parameter to create reconfigurable and hierarchical nanostructures. The work is high-quality, conceptually novel, and supported by rigorous characterization. I recommend the manuscript for publication after the authors address the following major points to further strengthen the manuscript quality.
1. The nanoDSC experiments were conducted at a scan rate of 1 °C min-1. In classical polymer physics, slow heating can induce "annealing" or lamellar thickening during the scan itself, which may shift the observed to higher values. It would be beneficial if the authors could clarify whether they observed any scan-rate dependency.
2. The authors attribute the localized recrystallization in the hollowed interior to a "solution-phase melt-memory effect." While this is a persuasive hypothesis, I would appreciate further clarification on the physical nature of this memory. Does it stem from the survival of sub-critical crystalline nuclei that remain below the detection limit of microscopy, or is it related to a local concentration gradient of polymer chains near the seed interface?
3. The observed values in solution are significantly lower than those of bulk PCL. While the Gibbs-Thomson analysis focuses on lamellar thickness, the solvent-polymer interaction (solvent swelling or surface energy effects) also plays a critical role in depressing the melting point. A brief mention or comparison of how the ethanol environment specifically influences the (surface free energy) term compared to bulk values would be insightful.
4. The manuscript reports an impressive 89% yield for hollow platelets under optimized thermal conditions (for 3 minutes). It would be helpful to include a brief comment in the Results or Supporting Information regarding the morphology of the remaining 11%. Are these primarily unreacted seeds or fully disassembled fragments? This would help define the robustness of the "thermally programmed" disassembly.
5. The author should perform selected area electron diffraction (SAED) experiment to find out the degree of crystallization in the 2D sheets.
6. The author should compare the different melting behaviour (Tm) in ranging from 35.7 °C for PCL45 to 38.5 °C for PCL90 with crystallinity behaviour (degree of crystallinity) using Wide-Angle X-ray Scattering (WAXS).
7. Please ensure that all scale bars (nm or m) in the TEM images should be mentioned either in the figure caption or in the images.
8. Authors may consider to cite some following recent articles on CDSA:
https://doi.org/10.1021/acs.chemrev.5c00298; https://doi.org/10.1038/s41428-024-00931-8; https://doi.org/10.1002/macp.202400426; https://doi.org/10.1039/D6CS00030D.

Reviewer #2

(Remarks to the Author)
The study by Turan and Arno deals with the fabrication of 2D platelets from CDSA using PCL systems. Firstly, the study is of high quality, well written and nicely presented. The information provided will allow for the study to reproduced. The study itself is original, and reports on the characterisation and exploitation of melting temperatures to design responsive PCL

platelets. While the field is swamped with PCL platelets and their syntheses, this communication contributes a new angle and insight into CDSA materials. 1) It provides a systematic solution-state melting study if CSA platelets; 2) It validates and extends the Gibbs-Thomson theory to CDSA platelets, and 3) it demonstrates that CDSA platelets can be programmed to become hollow AND self-heal by temperature (melting-recrystallisation cycles). Thus, this work fills knowledge gaps and introduces an innovative concept to the field. Overall, the paper is already at a high standard for publication, and the fundamental insights and evaluations are expertly presented. Where the authors could improve on is on relevance. To the CDSA community, this paper is gold, but what about the broader polymer and nanoscience community. Often CDSA is tipped to solve issues on nanomedicine or other areas where shape and function of materials is important. Anchoring the findings in here to where this behaviour might be useful will broaden the scope of this work. The authors may consider the following comments:
- Recrystallisation into interior voids is interesting. What happens to the original seed that was in the centre of the platelet? I would assume that this should act as a seed again when the solution is cooled, and that there will be crystallisation inside the original platelet, but also new platelets forming. Can the authors comment?
- For the “spontaneous decoupling” that releases the inner part of the platelet in Figure 4, what would happen if PCL is recrystallised like in Figure 5? Would only the interior regrow or would also the released smaller platelet grow? If the interior regrows, would this only lead to partially closed platelet (as PCL is insufficient)?
- With the seed missing in the re-crystallisation step in Figure 5, how can the interior fully close? Shouldn’t there be insufficient PCL to close it completely?
- I am puzzled by the fact that polymers are not re-crystallising in the interior AND also onto the exterior. Can the authors elaborate on this further? How is the exterior different from the interior? Proximity seems a far-fetched reason since PCL should be dissolved after his has been “etched” out. The authors state that “dissolved chains remain spatially confined within the hollow interior”. Can they proof it? Confocal microscopy?
- The recrystallisation (self-healing) is fascinating but of course needs to happen in ethanol. What happens when these platelets are transferred into water? What happens when they are heated? I presume the PCL cannot dissolve out into solution, but can it rearrange itself to open the interior of the platelet?
- The final sentence: “This unique and controllable behavior of 2D platelets prepared through CDSA can be exploited for the fabrication of thermally switchable nanoreactors, recyclable crystalline frameworks, and dynamically reconfigurable polymer assemblies, where structural function can be programmed solely through temperature.” How would the reported concept aid in nanoreactor performance/design? What might dynamically reconfigurable assemblies do and be good for? I know this is meant to be a futures statement but I struggle to see the links between the fundamental study and the applications it will enable. An example of how it could overcome a shortcoming would be good.

Reviewer #3

(Remarks to the Author)
In the manuscript entitled "Thermally programmed disassembly and reconstruction of 2D polymer platelets via melting-governed crystallization-driven self-assembly", a thorough study of the structural reconfiguration of CDSA-derived 2D platelets in solution is performed. The validation of Gibbs-Thomson behavior in finite, solution-grown 2D crystals is provided by quantitative correlations between lamellar thickness and melting.
The manuscript subject is of interest to the Nature Communications Journal. For publication, I suggest the following Comments to be considered:

Comment 1

Expressing units. The expression of units in all figures should be unified according to the journal policy. The way units are expressed should be checked both in the Main Manuscript and in the Supplementary Information and the necessary corrections should be made.
I mention the following inaccuracies that should be corrected:
● It is not proper using different units for the same parameter: in Figure S14 the temperature is expressed in oC, but in Figure S15, the temperature is expressed in K.
● It is not proper using different formats for expressing units. Although generally in the manuscript and in Supporting material the correct expression, according to SI units is used (example: mg mL-1, mJ m-2 etc), however there are some Figures where the notation it is not presented in the correct format. Thus, the notation on the Y-axis in the figures: Fig. 1(d), Fig. 2 (e), Fig. S7, Fig. S11, Fig. S20, Fig. S21(e), Fig. S24 and Fig. S25 should be corrected.

Comment 2

Within the Section: Effect of lamellar thickness on melting temperature, on the pag. 6, the last paragraph.
Please check the consistency of the information in the first sentence of this paragraph and rephrase it.
The text is referring to “the platelet melting temperatures for the (PCL90-b-PDMAx) diblock copolymers”, and as references are indicated (Supplementary Fig. 17-20 and Supplementary Table 2). However, neither in the Figures S17-20 nor in Table S2 are there any data regarding the melting temperatures of the (PCL90-b-PDMAx) diblock copolymers (In the Table 2 are found only the melting temperatures obtained by DSC for the PCL without corona block).
In fact, only in the last sentence of this paragraph, when referring to Fig. S21, the melting temperatures obtained by nano-DSC of the (PCL90-b-PDMAx) diblock copolymers are presented.

Comment 3

Within the Supporting Information, Section Methods, in the paragraph corresponding to Differential Scanning Calorimetry (DSC) method (pag.3), in the last sentence presenting this method it is written: “The total enthalpy of melting (ΔHm) was obtained by integrating and normalizing all endothermic transitions observed in the thermograms.” However, regarding the DSC data (and it is the same for nanoDSC), neither the Manuscript nor the Supporting Information present enthalpy values ΔHm, and no discussion is made regarding these data. The Figure S7 is presenting only the differential scanning calorimetry traces of PCL (heat flow in W/g) as a function of temperature. I suggest either revising the information in the description of the method, or including new data or a new discussion regarding the total enthalpy of melting. (In fact, it would add a new value to this paper, specifying what the other parameters derived from these methods say and if these data should be used to correlate with the results of the Gibbs–Thomson approach).

Comment 4

Within the Section: Effect of lamellar thickness on melting temperature, on the pag. 5,
the discussion about the platelets formed at different crystallization temperatures is made.
Here it is written:
“Platelets formed at different crystallization temperatures were analyzed by nanoDSC to determine their melting temperatures (Figure 2e). As expected, platelets with thinner lamellae exhibited lower melting endotherms than those with thicker lamellae….”
When you are referring to "lower melting endotherms" this involves analyzing both the peaks and the area under the peaks. From figure 2e (and it is the same for figure 1d) it is clear that both the peaks and the peak areas (representing the enthalpies - obtained in this case from nanoDSC analysis) - vary. Because you refer to the melting endotherms, it would be necessary to also refer to the peak area and (as in the previous comment 3), the significance of the enthalpy thermodynamic parameter obtained by nanoDSC should be noted. Instead, if you want to keep the discussion only to the melting temperatures, rephrasing the sentence is necessary.

Comment 5

Supporting Information, Figure S15, Caption of Figure 15, the equations below the Figure S15.
Please check the consistency of the notations and information in this part of the Supporting Material.
I note the following:
The caption of Figure S15 talks about the dependence of the equilibrium melting temperature of a finite lamellar crystal on its thickness. Therefore, it is necessary to specify which melting temperature is represented in Figure 5 by using the notation Tm (because the notation for the equilibrium melting temperature should be *T*m0). In addition, in the equations written below the Figure S15, both the notations Tm (*l*) and Tm appear expressing practically the same mathematical relation. It is understandable that by expressing Tm (l) the intention is to emphasize the dependence of Tm on (l). However, since this is a work that is to be published, the final notation to be used should be mentioned.
As a general Comment: It is necessary to verify the consistency of the notations regarding the different melting temperatures (resulting from different experimental and theoretical approaches) in correlation with the information given throughout the paper and Supporting Material.

Version 1:

Reviewer comments:

Reviewer #1

(Remarks to the Author)
Authors have addressed all the comments raised by the reviewer. This manuscript can now be published it its current form.

Reviewer #2

(Remarks to the Author)
The authors have sufficiently addressed the reviewers' comments and made amendments to the text and discussion which also made the manuscript even stronger. I recommend publication.

Reviewer #3

(Remarks to the Author)
The subject addressed in this article is actual and important for the future development of thermally stimulated, functionally responsive crystalline nanostructures.

The authors answered the concerns addressed in my previous review and the corresponding changes were made in the

manuscript. The inconsistencies were corrected and the clarity of the text when referring to the different methods of characterization has been improved.

Open Access This Peer Review File is licensed under a Creative Commons Attribution 4.0 International License, which permits use, sharing, adaptation, distribution and reproduction in any medium or format, as long as you give appropriate credit to the original author(s) and the source, provide a link to the Creative Commons license, and indicate if changes were made.
In cases where reviewers are anonymous, credit should be given to 'Anonymous Referee' and the source.
The images or other third party material in this Peer Review File are included in the article's Creative Commons license, unless indicated otherwise in a credit line to the material. If material is not included in the article's Creative Commons license and your intended use is not permitted by statutory regulation or exceeds the permitted use, you will need to obtain permission directly from the copyright holder.
To view a copy of this license, visit https://creativecommons.org/licenses/by/4.0/

We thank the reviewers for their positive comments and suggestions. We have made changes in the manuscript to strengthen our work in response to the reviewers' concerns. Please find a point-by-point response to each comment below.

Reviewer 1:

This manuscript presents a sophisticated study on the thermal properties of 2D polymer platelets, effectively bridging the gap between classical polymer physics and solution-state crystallization-driven self-assembly (CDSA). The authors demonstrate that the melting temperature (Tm) of solution-grown crystals can be utilized as a precise design parameter to create reconfigurable and hierarchical nanostructures. The work is high-quality, conceptually novel, and supported by rigorous characterization. I recommend the manuscript for publication after the authors address the following major points to further strengthen the manuscript quality.

1. The nanoDSC experiments were conducted at a scan rate of 1 °C min^{-1}. In classical polymer physics, slow heating can induce "annealing" or lamellar thickening during the scan itself, which may shift the observed to higher values. It would be beneficial if the authors could clarify whether they observed any scan-rate dependency.

To assess the influence of scan rate on the observed melting temperatures, we conducted nanoDSC experiments at scan rates of 0.5 and 2 °C min^{-1} for PCL_{45} platelets crystallized at 20 °C. A scan rate of 0.5 °C min^{-1} produced a less well-defined melting transition, whereas a rate of 2 °C min^{-1} led to a broader peak with no major shift in the observed melting temperature. As such, a scan rate of 1 °C min^{-1} was found to be optimal and not affected by scan-induced annealing. This clarification has now been added to the manuscript, together with the additional nanoDSC data in Supplementary Figure 10C.

2. The authors attribute the localized recrystallization in the hollowed interior to a "solution-phase melt-memory effect." While this is a persuasive hypothesis, I would appreciate further clarification on the physical nature of this memory. Does it stem from the survival of sub-critical crystalline nuclei that remain below the detection limit of microscopy, or is it related to a local concentration gradient of polymer chains near the seed interface?

We thank the reviewer for this insightful comment. To further investigate the physical origin of the proposed localized recrystallization in the hollow interior, a series of competition experiments were performed to distinguish between two possible mechanisms: (i) local enrichment of polymer chains near the interior, and (ii) surviving sub-critical crystalline nuclei templating subsequent recrystallization. To emulate the distinct interior and exterior environments of hollow platelets, competition experiments were conducted using PCL_{90} homopolymer platelet seeds (HP seeds), and PCL_{90}/PCL_{90}-*b*-$PDMA_{150}$ platelet seeds (BCP seeds) (Supplementary Figure 37). A slight preference for crystallization onto HP seeds was observed at lower crystallization temperatures, suggesting that interfacial accessibility can influence chain deposition. To directly probe the recrystallization process, a thermally labile PCL_{45} outer layer was grown on HP seeds and selectively melted before recrystallization (Supplementary Figure 38). Under static conditions (*i.e.* no stirring), 95% of the recrystallized material deposited back onto the original HP seeds. When the experiment was repeated under

stirring conditions, preference for recrystallization onto the original HP seeds remained (72%). Similar behavior was observed in analogous experiments using double-layer BCP seeds (Supplementary Figure 39). These results suggest that local enrichment of polymer chains near the original interface contributes to the observed recrystallization behavior under static conditions but cannot fully account for the observed preference. The preferential recrystallization under stirring indicates that surviving sub-critical crystalline nuclei or partially disrupted crystalline domains likely contribute to templating subsequent crystal growth. We have now expanded the discussion in the manuscript to clarify this distinction and to emphasise that the observed behavior likely arises from contributions of both local chain enrichment and surviving sub-critical nuclei.

3. The observed values in solution are significantly lower than those of bulk PCL. While the Gibbs-Thomson analysis focuses on lamellar thickness, the solvent-polymer interaction (solvent swelling or surface energy effects) also plays a critical role in depressing the melting point. A brief mention or comparison of how the ethanol environment specifically influences the (surface free energy) term compared to bulk values would be insightful.

We agree that, in addition to the finite lamellar thickness, solvent-polymer interactions lower the melting point of our nanostructures compared to bulk PCL. To clarify this point, we have added a brief discussion noting that the experimentally determined $T^0_{m(s)}$ values implicitly incorporate the influence of the ethanol medium, which modifies the interfacial free energy contribution compared to bulk PCL.

4. The manuscript reports an impressive 89% yield for hollow platelets under optimized thermal conditions (for 3 minutes). It would be helpful to include a brief comment in the Results or Supporting Information regarding the morphology of the remaining 11%. Are these primarily unreacted seeds or fully disassembled fragments? This would help define the robustness of the "thermally programmed" disassembly.

The remaining 11% morphology consists of partially disassembled fragments with a minor proportion of unreacted platelets. This has now been clarified in the main text (and also shown in Supplementary Figure 31G).

5. The author should perform selected area electron diffraction (SAED) experiment to find out the degree of crystallization in the 2D sheets.

We thank the reviewer for this suggestion. SAED analysis and determination of the degree of crystallinity from the extracted radial intensity profiles proved to be a valuable addition to our work. This analysis demonstrates that platelet crystallinity increases with increasing PCL chain length (PCL_{25} to PCL_{90}), consistent with the crystallinity trends obtained by nanoDSC and WAXS analyses of the platelets. The higher degree of crystallinity observed for longer PCL chains explains their higher melting temperatures. A comparison of these complementary measurements has now been included in the revised manuscript and Supplementary Figure 13.

6. The author should compare the different melting behaviour (Tm) in ranging from 35.7 °C for PCL45 to 38.5 °C for PCL90 with crystallinity behaviour (degree of crystallinity) using Wide-Angle X-ray Scattering (WAXS).

To further investigate the relationship between crystallinity and thermal behavior, we conducted WAXS analysis of both bulk PCL samples and PCL platelets and compared the

crystallinity values with those determined using other methods such as nanoDSC, DSC, and SAED. This comparison demonstrates good agreement between these techniques and highlights the differences in crystallinity between the bulk polymers and self-assembled platelets. Across all platelet characterization methods, the trend is that crystallinity increases with increasing PCL chain length, whereas the opposite trend is observed for bulk polymers, where crystallinity decreases with increasing chain length. We have added a discussion around these contrasting trends in the revised manuscript, together with the corresponding WAXS data in Supplementary Figure 12.

7. Please ensure that all scale bars (nm or μm) in the TEM images should be mentioned either in the figure caption or in the images.

Scale bar values have now been added to all figure captions where appropriate.

8. Authors may consider to cite some following recent articles on CDSA: https://doi.org/10.1021/acs.chemrev.5c00298; https://doi.org/10.1038/s41428-024-00931-8;https://doi.org/10.1002/macp.202400426; https://doi.org/10.1039/D6CS00030D.

As suggested, the above references have now been added.

Reviewer 2:

The study by Turan and Arno deals with the fabrication of 2D platelets from CDSA using PCL systems. Firstly, the study is of high quality, well written and nicely presented. The information provided will allow for the study to reproduced. The study itself is original, and reports on the characterisation and exploitation of melting temperatures to design responsive PCL platelets. While the field is swamped with PCL platelets and their syntheses, this communication contributes a new angle and insight into CDSA materials. 1) It provides a systematic solution-state melting study if CSA platelets; 2) It validates and extends the Gibbs-Thomson theory to CDSA platelets, and 3) it demonstrates that CDSA platelets can be programmed to become hollow AND self-heal by temperature (melting-recrystallisation cycles). Thus, this work fills knowledge gaps and introduces an innovative concept to the field. Overall, the paper is already at a high standard for publication, and the fundamental insights and evaluations are expertly presented. Where the authors could improve on is on relevance. To the CDSA community, this paper is gold, but what about the broader polymer and nanoscience community. Often CDSA is tipped to solve issues on nanomedicine or other areas where shape and function of materials is important. Anchoring the findings in here to where this behaviour might be useful will broaden the scope of this work. The authors may consider the following comments:

1. Recrystallisation into interior voids is interesting. What happens to the original seed that was in the centre of the platelet? I would assume that this should act as a seed again when the solution is cooled, and that there will be crystallisation inside the original platelet, but also new platelets forming. Can the authors comment?

We thank the reviewer for this interesting point. Our flash-frozen PCL seeds are prepared using a flow reactor and differ from classically annealed PCL seeds. Owing to their reduced thermal annealing history, these seeds show a lower melting temperature ($T_{m(s)}$ = 32.4 °C, Supplementary Figure 34). As a result, seeds are fully melted after thermal treatment at 37 °C, and act as additional source of polymer chains available for subsequent recrystallization.

This is only observed when seeds are isolated in solution, whereas in triple-layer platelets the seeds remain protected by the surrounding PCL_{90}. This discussion has now been added to the manuscript.

2. For the "spontaneous decoupling" that releases the inner part of the platelet in Figure 4, what would happen if PCL is recrystallised like in Figure 5? Would only the interior regrow or would also the released smaller platelet grow? If the interior regrows, would this only lead to partially closed platelet (as PCL is insufficient)?

In the case of recrystallization following spontaneous decoupling, both the released inner entity and the interior of the hollow platelet are able to regrow (Supplementary Figure 40B). This is because both entities retain crystalline nuclei that can template subsequent chain deposition. As a result, the available PCL chain pool is distributed between the two competing nucleation sites, leading to the formation of partially closed platelets and double layer inner platelets.

3. With the seed missing in the re-crystallisation step in Figure 5, how can the interior fully close? Shouldn't there be insufficient PCL to close it completely?

The full closure of the interior during recrystallization occurs because of the presence of multiple available polymer reservoirs: (i) PCL_{45} originating from the thermally labile inner layer, (ii) PCL_{50}-*b*-$PDMA_{200}$ released from dissolved seeds, and (iii) PCL_{90}/PCL_{90}-*b*-$PDMA_{150}$ derived from platelets showing structural failure during the melting process. Together, these species provide sufficient PCL to cover the platelet interior completely. It is also noted that 22% of observed structures do not fully close and instead exhibit partially filled interiors (Supplementary Figure 36).

4. I am puzzled by the fact that polymers are not re-crystallising in the interior AND also onto the exterior. Can the authors elaborate on this further? How is the exterior different from the interior? Proximity seems a far-fetched reason since PCL should be dissolved after his has been "etched" out. The authors state that "dissolved chains remain spatially confined within the hollow interior". Can they proof it? Confocal microscopy?

We thank the reviewer for raising this point, which helps us clarifying this concept further. The preferential recrystallization observed in hollow platelet systems does not solely rely on physical confinement of dissolved chains within the interior. To prove this, we designed a series of competition experiments using HP and BCP platelet seeds (see response to Reviewer 1, point 2), and exploited stirring-controlled recrystallization (Supplementary Figures 37-39). Under stirring conditions, a significant preference for recrystallization onto the original HP seeds (72%) demonstrates that this effect cannot be explained by spatial confinement or proximity alone. Instead, these results are consistent with a templated recrystallization mechanism in which surviving sub-critical crystalline nuclei and partially disrupted domains act as the main sites for new crystal growth. We have therefore revised the manuscript to clarify that recrystallization is governed by a kinetically biased competition between available nucleation sites, with the interior environment providing a higher effective nucleation density following selective melting. Importantly, these experiments were necessary as the use of confocal microscopy, as suggested by this reviewer, did not allow to achieve a sufficient distinction of BODIPY-labelled unimer features from background noise.

5. The recrystallisation (self-healing) is fascinating but of course needs to happen in ethanol. What happens when these platelets are transferred into water? What happens when they are heated? I presume the PCL cannot dissolve out into solution, but can it rearrange itself to open the interior of the platelet?

After transfer to water through dialysis the double-layer platelets remain intact and still undergo selective melting of the inner layer to form hollow platelets, although at a higher temperature (50 °C, Supplementary Figure 35), compared to the observed melting in ethanol (37 °C). In water, however, the released PCL does not remain dispersed and instead forms small spherical aggregates, visible on TEM and AFM images.

6. The final sentence: “This unique and controllable behavior of 2D platelets prepared through CDSA can be exploited for the fabrication of thermally switchable nanoreactors, recyclable crystalline frameworks, and dynamically reconfigurable polymer assemblies, where structural function can be programmed solely through temperature.” How would the reported concept aid in nanoreactor performance/design? What might dynamically reconfigurable assemblies do and be good for? I know this is meant to be a futures statement but I struggle to see the links between the fundamental study and the applications it will enable. An example of how it could overcome a shortcoming would be good.

We agree that the original statement was overly broad and have revised the concluding discussion to provide a more specific example. In particular, the ability to selectively melt and subsequently recrystallize defined regions of a platelet offers a potential route to assemblies where functional species, such as catalysts, can be reversibly exposed and re-encapsulated through temperature alone. Such systems could address a common challenge in recovery and recycling of catalytic components after use. Furthermore, as melting temperature is an inherent property of semicrystalline polymers, we anticipate that the general framework presented here could be extended to other polymer systems and/or morphologies.

Reviewer 3:

In the manuscript entitled "Thermally programmed disassembly and reconstruction of 2D polymer platelets via melting-governed crystallization-driven self-assembly", a thorough study of the structural reconfiguration of CDSA-derived 2D platelets in solution is performed. The validation of Gibbs-Thomson behavior in finite, solution-grown 2D crystals is provided by quantitative correlations between lamellar thickness and melting.
The manuscript subject is of interest to the Nature Communications Journal. For publication, I suggest the following Comments to be considered:

1. Expressing units. The expression of units in all figures should be unified according to the journal policy. The way units are expressed should be checked both in the Main Manuscript and in the Supplementary Information and the necessary corrections should be made.
I mention the following inaccuracies that should be corrected:
● It is not proper using different units for the same parameter: in Figure S14 the temperature is expressed in oC, but in Figure S15, the temperature is expressed in K.
● It is not proper using different formats for expressing units. Although generally in the manuscript and in Supporting material the correct expression, according to SI units is used (example: mg mL-1, mJ m-2 etc), however there are some Figures where the notation it is not

presented in the correct format. Thus, the notation on the Y-axis in the figures: Fig. 1(d), Fig. 2 (e), Fig. S7, Fig. S11, Fig. S20, Fig. S21(e), Fig. S24 and Fig. S25 should be corrected.

We thank this reviewer for pointing out these inconsistencies. All temperature units have now been standardized, and the formatting of units has been corrected where necessary to ensure consistency with the journal's guidelines and Supplementary Information notation.

2. Within the Section: Effect of lamellar thickness on melting temperature, on the pag. 6, the last paragraph. Please check the consistency of the information in the first sentence of this paragraph and rephrase it. The text is referring to “the platelet melting temperatures for the (PCL90-b-PDMAx) diblock copolymers”, and as references are indicated (Supplementary Fig. 17-20 and Supplementary Table 2). However, neither in the Figures S17-20 nor in Table S2 are there any data regarding the melting temperatures of the (PCL90-b-PDMAx) diblock copolymers (In the Table 2 are found only the melting temperatures obtained by DSC for the PCL without corona block). In fact, only in the last sentence of this paragraph, when referring to Fig. S21, the melting temperatures obtained by nano-DSC of the (PCL90-b-PDMAx) diblock copolymers are presented.

We agree with this reviewer that the original wording could be misinterpreted. To improve clarity, we have revised the text to explicitly distinguish between the characterization of the diblock copolymers (now Supplementary Figures 20-23 and Supplementary Table 2) and the nanoDSC melting-temperature measurements (Supplementary Figure 24).

3. Within the Supporting Information, Section Methods, in the paragraph corresponding to Differential Scanning Calorimetry (DSC) method (pag.3), in the last sentence presenting this method it is written: “The total enthalpy of melting (ΔHm) was obtained by integrating and normalizing all endothermic transitions observed in the thermograms.” However, regarding the DSC data (and it is the same for nanoDSC), neither the Manuscript nor the Supporting Information present enthalpy values ΔHm, and no discussion is made regarding these data. The Figure S7 is presenting only the differential scanning calorimetry traces of PCL (heat flow in W/g) as a function of temperature. I suggest either revising the information in the description of the method, or including new data or a new discussion regarding the total enthalpy of melting. (In fact, it would add a new value to this paper, specifying what the other parameters derived from these methods say and if these data should be used to correlate with the results of the Gibbs–Thomson approach).

Both DSC and nanoDSC melting peaks were analysed by integrating all observed melting transitions to obtain the enthalpy of melting. These values were then normalised to sample mass (and scan rate in the case of nanoDSC peaks) and used to calculate the degree of crystallinity for both the bulk polymers and the self-assembled platelets. The resulting trends in crystallinity were directly compared with those obtained from SAED and WAXS analyses, providing good overall agreement between the different techniques. A consistent trend is observed for platelets, where the degree of crystallinity increases with increasing PCL chain length; we have since correlated this trend with the observed increase in melting temperatures. In contrast, the bulk polymers show a different behavior, with crystallinity decreasing with increasing chain length, consistent with previously reported trends for bulk PCL. These results are discussed in the revised manuscript along with the new data reported in Supplementary Figures 11-13.

4. Within the Section: Effect of lamellar thickness on melting temperature, on the pag. 5, the discussion about the platelets formed at different crystallization temperatures is made. Here it is written: “Platelets formed at different crystallization temperatures were analyzed by nanoDSC to determine their melting temperatures (Figure 2e). As expected, platelets with thinner lamellae exhibited lower melting endotherms than those with thicker lamellae….” When you are referring to "lower melting endotherms" this involves analyzing both the peaks and the area under the peaks. From figure 2e (and it is the same for figure 1d) it is clear that both the peaks and the peak areas (representing the enthalpies - obtained in this case from nanoDSC analysis) - vary. Because you refer to the melting endotherms, it would be necessary to also refer to the peak area and (as in the previous comment 3), the significance of the enthalpy thermodynamic parameter obtained by nanoDSC should be noted. Instead, if you want to keep the discussion only to the melting temperatures, rephrasing the sentence is necessary.

We thank the reviewer for this comment. We have revised the wording to avoid ambiguity between peak position and integrated peak area. In this section, our discussion focused on the melting temperature. The corresponding changes in peak area from nanoDSC integration have been reported in Supplementary Figure 18 and discussed alongside the crystallinity analysis.

5. Supporting Information, Figure S15, Caption of Figure 15, the equations below the Figure S15. Please check the consistency of the notations and information in this part of the Supporting Material. I note the following: The caption of Figure S15 talks about the dependence of the equilibrium melting temperature of a finite lamellar crystal on its thickness. Therefore, it is necessary to specify which melting temperature is represented in Figure 5 by using the notation Tm (because the notation for the equilibrium melting temperature should be Tm0). In addition, in the equations written below the Figure S15, both the notations Tm (l) and Tm appear expressing practically the same mathematical relation. It is understandable that by expressing Tm (l) the intention is to emphasize the dependence of Tm on (l). However, since this is a work that is to be published, the final notation to be used should be mentioned. As a general Comment: It is necessary to verify the consistency of the notations regarding the different melting temperatures (resulting from different experimental and theoretical approaches) in correlation with the information given throughout the paper and Supporting Material.

We have revised the notation throughout the Supporting Information and manuscript to ensure consistency. In particular, we now clearly distinguish between the solution melting temperature ($T_{m(s)}$) obtained from nanoDSC and the corresponding equilibrium melting temperature ($T_m{}^0{}_{(s)}$) extrapolated from these values. These corrections have been applied throughout the main manuscript and the Supporting Information.
